# Magnetic induction heating: where will the field go next?

**DOI:** 10.1039/d5cs01155h

**Published:** 2026-10-14

**Authors:** Annah J. Wilson, Ehsan Sadeghi, Niccolò Silvestri, Eleonora Giannotta, Diba Farmanbordar, Giuseppe Maruccio, Teresa Pellegrino

**Affiliations:** a Istituto Italiano di Tecnologia via Morego 30 16163 Genoa Italy Teresa.Pellegrino@iit.it; b University of Salento Via per Monteroni 73100 Lecce Italy

## Abstract

Under the broader concept of magnetic induction (MI), in which magnetic fields are used to induce a current in conductors, magnetic induction heating (MIH) is known in oncotherapy for tumour ablation in what is called magnetic hyperthermia. However, a multitude of wider applications of MIH are beginning to shape opportunities at the interface of materials fabrication. This includes organic and inorganic synthesis, a comprehensive spectrum of biomedical needs, sensing, diagnostics, industrial processing, sustainable technologies, power generation and locomotion. A comparative analysis of reported MI studies is given grouped by application type, providing insight into the different materials employed and temperatures achieved. While MIH is predominantly based on magnetic materials that dissipate energy through hysteresis and Néel/Brownian relaxation losses, electrically conductive materials can also generate heat *via* eddy-current-induced Joule heating, although they remain relatively underexplored in biomedical applications. We examine the field parameters, the set-ups used and experimental configurations to provide a clear overview of the current landscape. In chemistry these applications will interest researchers in areas such as materials synthesis, electrocatalysis and sustainable chemistry. In the biomedical landscape, we explore studies from neuropathology to endometriosis and ocular disease, and even dental applications. Thus, we enable both specialists and researchers from adjacent fields to contribute with fresh perspectives. This review examines MIH through a new lens, highlighting underrepresented and recent developments and identifying promising directions to accelerate progress across different areas of science.

## Abstract 2

This review explores the diverse emerging uses of magnetic induction heating across a broad range of fields, highlighting recent developments and potential future directions.

## Graphical abstract

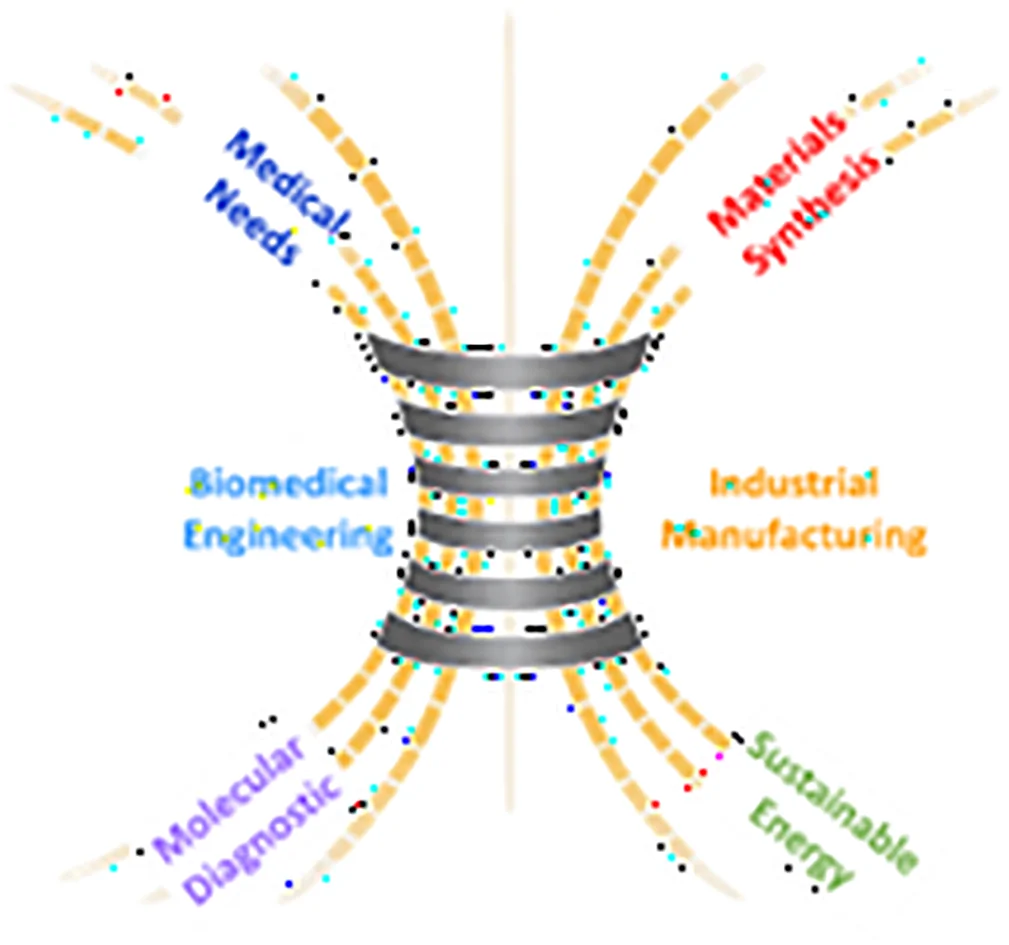

## 1. Introduction

Magnetic induction (MI) or electromagnetic induction is the process by which a time-varying magnetic field (MF) gives rise to an electric field (EF), a fundamental relationship detailed in Faraday's law of electromagnetism. This phenomenon drives the generation of electrical energy, such as in generators, and the induction of currents in conductors. MI is essential in a wide range of systems, such as power transmission and electronics. In recent decades, advances in materials science, nanotechnology, and biomedical engineering have expanded the scope of MI from macroscopic conductive systems to nanoscale and biological environments. Magnetic induction heating (MIH) sits under the wider MI concept. In this specific context, it refers to the mechanism used to generate heat when magnetic nanoparticles (MNPs) act as heat transducers under a time-varying magnetic field of kHz regime. MIH has a firm foothold in biomedicine, notably in the remote treatment of solid tumours with years of research progressing to pre-clinical and clinical studies.^1–3^ To date, glioblastoma multiforme (GBM) treated with iron oxide MNPs (NanoTherm® system) treated under a patient-safe alternating magnetic field (AMF), represents the first clinical regulatory approval of an MNP-mediated magnetic hyperthermia therapy in Europe. However, it is worth noting that there are emerging pre-clinical applications in other cancers such as prostate^4^ and pancreatic.^5^ In MIH, an AMF as a time-varying field in the kHz regime is applied to MNPs which dissipate electromagnetic energy as heat through magnetic relaxation and hysteresis losses (namely hysteresis and Néel/Brownian relaxation). This generates a temperature change where the MNPs are located. In the context of oncology, this offers a non-invasive and local method to achieve tissue penetration for deep cancer targeting, selectively heating the tumour. However, beyond this well-established research area, the unique ability of magnetic materials to generate tunable heating has opened the door to a new generation of biomedical and technological applications. Indeed, a number of comprehensive and insightful reviews can be found that offer a summary of progress in oncological applications of MIH to date, with some considering scope beyond oncology.^6–13^ However, in this review, based on recent literature, we aim to widely extend these perspectives, giving the spotlight to emerging studies and highlighting areas that may benefit from renewed focus. In compiling this review, we have uncovered a broad range of emerging applications of MI. Across the biomedical field, recent studies have looked to address areas of unmet medical needs (Fig. 1) such as inflammatory diseases including endometriosis, glaucoma, neurodegenerative diseases and spinal cord injury. Through these unique approaches there is clear demonstration of innovative thinking, with exploration of unconventional methodology for solving complex problems. Similarly, we see a drive towards novel approaches in biomedical engineering (Fig. 5) for MNPs internalization or genetic material delivery into the cells, for medical implants such as stent removal, for biofilms treatment including dental implant sterilization, and for molecular diagnostics, with exciting progress in MI sensing and heat mediated PCR applications (Fig. 8). In industrial and chemical contexts, MI emerges as a versatile alternative to conventional heating strategies that can have limitations in control and scalability. The localized, rapid nature of MI makes it particularly attractive for applications in organic and inorganic synthesis (Fig. 9) such as inorganic NPs, organic molecules, polymer molecules and hybrid organic/inorganic systems such as polymer shells on top of MNPs, but also for many sintering and curing processes (Fig. 10) of a variety of ceramics and polymer materials. Beyond this, manufacturing processes extend to drug amorphization and, even food pasteurization among others. In this field, the most heavily researched application is MI-mediated catalysis with several examples of reactions and products obtained through heat-induced MI (Fig. 12). These processes benefit not only from the thermal precision of MI but also from its scalability and compatibility with flow systems, making it a promising platform for sustainable and energy-efficient manufacturing. Considerable efforts have positioned MI as a tool for advancing sustainable technologies (Fig. 13), including wastewater treatment, water electrolysis, soil remediation, and CO_2_ capture. Less common, pioneering studies, still largely at the proof-of-concept stage, have explored the use of MI-generated heat to induce thermophoresis (Fig. 14), enabling locomotion or levitation effects and driving the movement of materials within liquid media. In each case, we provide a concise outline of the application and materials used, later drawing comparison with the materials used in established MIH avenues.

Across the studies analysed, a key consideration is the nature of the materials employed and the mechanisms underlying MI-induced heating. The majority of reports focus on magnetic materials, where heat generation is related to magnetization dynamics, including Néel and Brownian relaxation as well as hysteresis losses. The relative contribution of these mechanisms depends strongly on the magnetic regime of the particles, including their domain state and magnetic ordering (*e.g.*, superparamagnetic single domain, *versus* blocked single-domain or multi-domain and ferrimagnetic or ferromagnetic or antiferromagnetic ordering) as well as their physical state, including aggregation and the degree of rotational freedom. Comprehensive reviews have extensively covered the theoretical aspects of magnetic heating, and readers are referred to these works for a detailed discussion of relaxation and hysteresis mechanisms.^14–17^

In contrast, an emerging class of systems exploits Joule heating arising from eddy currents in electrically conductive media, including recent demonstrations utilizing liquid metals.

Therefore, distinguishing between magnetic-loss- and eddy-current-driven heating is essential for understanding MIH across different materials classes, dimensions, and operating conditions. For a brief summary, we here highlight the main heating concepts. For superparamagnetic MNPs, heat is generated through Néel and Brownian relaxation mechanisms, where energy is dissipated by the internal reorientation of magnetic moments or by the physical rotation of the entire nanoparticle under an AMF. In this regime, the linear response theory (LRT)^18^ correlates heat dissipation (*i.e.* opening of the hysteresis loop) due to dephasing of the magnetic moments with respect to the time varying field applied with nanoparticle parameters (*M*_s_, *K*, *etc.*) and field parameters (*f* and *H*). However, this theory is only valid for small superparamagnetic nanoparticles (<18 nm) under low field amplitudes, where the magnetization response remains linear. For ferromagnetic nanoparticles or particles near or larger to the superparamagnetic–ferromagnetic transition size, heat generation is better described by the Stoner–Wohlfarth model.^19^ In this case, heating arises from hysteresis losses associated with irreversible magnetization reversal, and the dissipated energy is proportional to the hysteresis loop area. Single-domain nanoparticles with uniaxial anisotropy and coercive fields close to, but lower than, the applied field exhibit the highest heating efficiency. Finally, in multidomain particles, magnetization reversal occurs through domain-wall motion, resulting in lower heat dissipation. While LRT and the Stoner–Wohlfarth model describe the superparamagnetic and ferromagnetic case limits, respectively, kinetic Monte Carlo models provide a more general framework by combining hysteresis and relaxation mechanisms across different magnetic regimes.^17^

Joule heating produced by eddy currents is comparatively less discussed in the context of nanoscale MIH systems but becomes particularly relevant in conductive media, especially at higher frequencies and in systems with larger characteristic length scales. Its theoretical foundations are well established in classical electrodynamics and induction heating literature.^20,21^ According to Faraday's law, a time-varying magnetic field induces electric fields that generate circulating currents in conductive regions, leading to power dissipation through Ohmic losses. Indeed, when electrons move through a conductor, they collide with atoms, defects, impurities, or phonons (lattice vibrations). These collisions convert part of the electrical energy into heat. The local volumetric power density (*P*) which provide information on how much electrical energy is converted into heat, can be expressed as *P* = *J*·*E* = *σE*^2^, where *σ* is the electrical conductivity, *E* the induced electric field and *J* the current density.

An order-of-magnitude estimate of the Ohmic losses can be obtained by the Faraday's law 
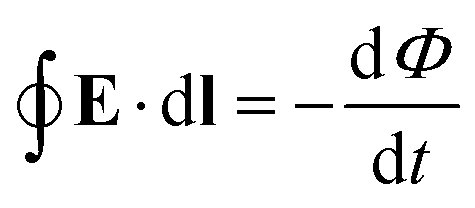
, with *Φ* the magnetic flux through the loop and assuming a uniform magnetic field *B*(*t*) = *B*_0_ sin(*ωt*) over a region of characteristic size *L*, quasi-static conditions (wavelength ≫ *L*), and negligible skin effect, the induced electric field scales as *E* ∼ *ωBL*, leading to a dissipated power density scaling as *P* ∝ *σ*(*ωB*)^2^*L*^2^, where *ω* is the angular frequency of the applied field. Thus, Joule heating due to eddy currents scale with the squared size of the material and is increasingly relevant at high frequencies and in systems with extended conductive domains and significant electrical conductivity.

Another relevant aspect of this mechanism is its non-locality strictly related to the macroscopic electrical properties of the medium. Therefore, the relative contribution of Joule and magnetic losses can vary significantly depending on the experimental conditions: Joule heating is typically negligible at the nanoscale but potentially dominant in bulk conductive matrices or when magnetic nanoparticles are embedded within conductive scaffolds. These characteristics make Joule heating normally more relevant in industrial and environmental applications, where volumetric heating or selective activation of conductive phases is desired. On the other hand, its limited spatial selectivity and the potential for non-specific bulk heating limit its application in biomedical contexts, except in the case of conductive tissues, implanted devices, or high-frequency operation.^22^ These considerations provide useful design and interpretation rules for optimizing material systems, accurately evaluating heating efficiency, and enable practical applications across different domains.^23^

Overall, our goal with this review is to broaden the scope of the field, drawing attention to recent and underexplored applications of MI, while extending the discussion beyond conventional magnetic materials to include emerging MIH approaches based on eddy-current heating in electrically conductive materials.^24^ Moreover, our systematic literature review reveals a critical lack of standardized reporting for magnetic field parameters in MIH/MH experiments. Beyond instrumental heterogeneity, we also draw attention on the significant inconsistency in disclosing essential methodological details, such as coil geometry, field homogeneity, and cooling efficiency.

We also focus on real-world applications, with translation as a key theme.^16,18,25,26^ The topics are presented in an accessible and structured manner to engage researchers across disciplines and broaden their perspectives on the capabilities of MI beyond their own fields of application. Indeed, by surveying these diverse use cases, from clot dissolution to catalyst activation, and integrating emerging concepts with established expertise, we aim to illuminate the untapped potential of MI as a cross-disciplinary modality and to stimulate further innovation in this expanding field. This allows us to look forwards and ultimately try to answer the question: where will the field go next?

## 2. Innovation across biomedicine

In recent years, MI has seen a wide range of developments outside of traditional oncological applications. In the following section, we will explore and analyse these advances that have been tackled so far in biomedicine, bioengineering and molecular diagnostics, giving insight and linking to future directions (Fig. 1).

**Fig. 1:**
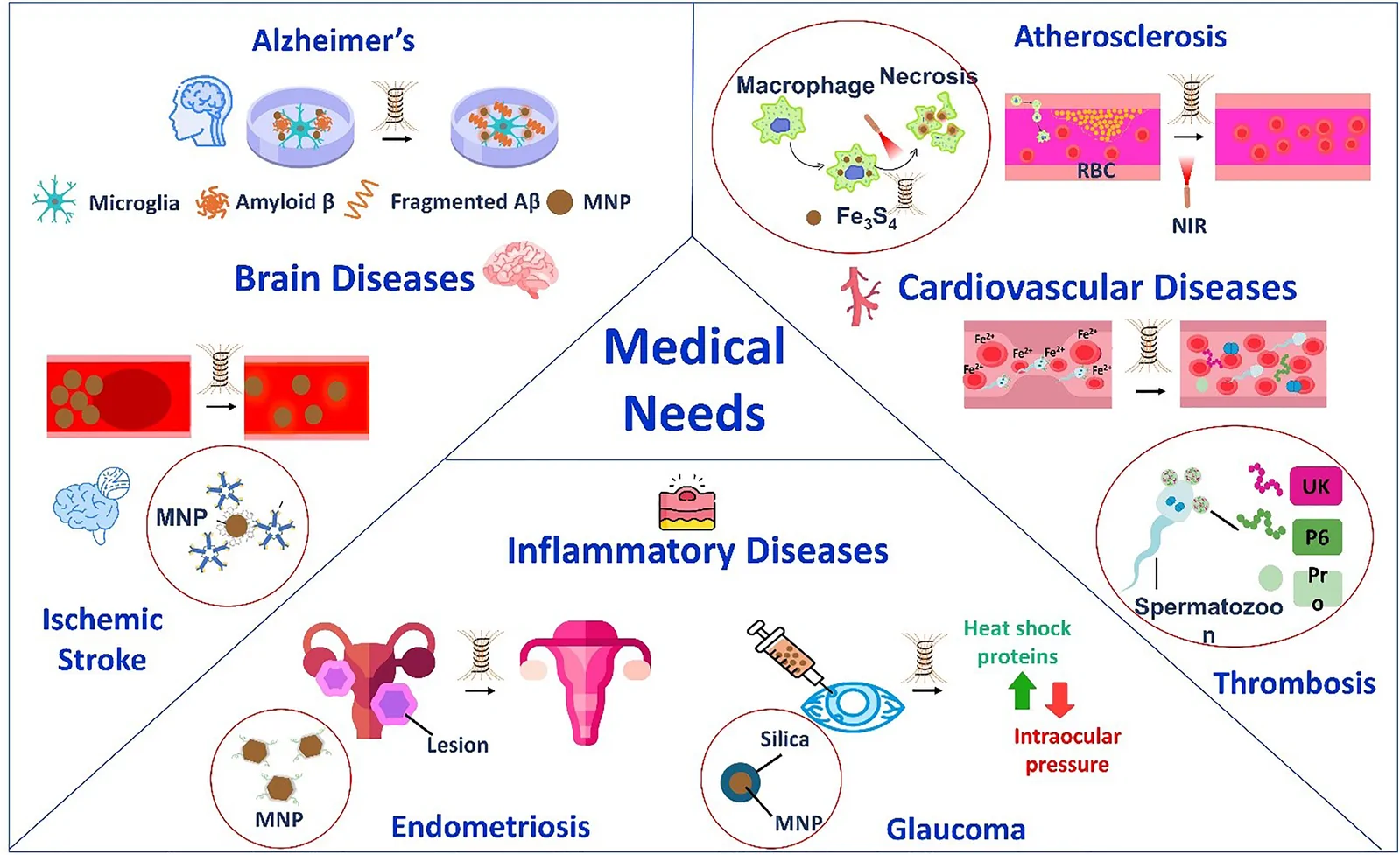
A schematic view to fully summarize MI for unmet medical needs. (left) Diseases of the brain including plaque disruption in Alzheimer's disease^27^ [adapted/reproduced from ref. 27 with permission from Elsevier,^27^ Copyright 2021] and clot dissolution in ischemic stroke [adapted/reproduced from ref. 28 under the Creative Commons CC BY 4.0 license,^28^ Copyright 2022].^28^ (right) cardiovascular disease applications so far explored such as atherosclerosis^29^ stroke [adapted/reproduced from ref. 29 under the Creative Commons CC BY 4.0 license,^29^ Copyright 2020] and thrombosis stroke [adapted/reproduced from ref. 30 under the Creative Commons CC BY 4.0 license,^30^ Copyright 2024].^30^ (bottom) Inflammatory condition treatment such as lesion targeting in endometriosis^31^ [adapted/reproduced from ref. 31 with permission from John Wiley and Sons,^31^ Copyright 2022] and induction of heat shock proteins for reducing intraocular pressure in glaucoma [adapted/reproduced from ref. 32 with permission from Elsevier,^32^ Copyright 2022].^32^

### 2.1 MI for unmet medical needs

#### 2.1.1 Diseases of the brain

While the primary focus of this review is to highlight new disease applications, it is perhaps interesting to begin at the core of medical MI research, highlighting relevant advances in GBM. For MIH, extensive pre-clinical studies are essential to the successful clinical translation of MNPs as novel imaging probes and therapeutic agents with few but significant clinical trials carried out on the treatment of patient solid tumours with MIH, and with established cell and animal models being the most common way to evaluate biocompatibility and efficacy.^33–35^ However, there has been a recent shift, encouraging a move away from traditional systems and reducing the number of animals used in global research. This has driven development of alternatives such as microfluidic systems, commonly referred to as 'tumour-on-a-chip' models which can more accurately, replicate the tumour microenvironment.^36^ Such models signify an interesting prospect for MIH studies, where observed outcomes are heavily influenced by the parameters of a model, such as cell density and tumour size.^37^ In 2020, Manami *et al.*^38^ explored the application of MIH in GBM using a tumour-on-a-chip model. This concise study utilises commercially available fluidMAG-Amine MNPs, which are comprised of a magnetite core with a biocompatible aminosilane coating, with a measured SAR of 115.4 ± 6.0 W g^−1^ (24 kA m^−1^ and 305 kHz). C6 rat glioma cells were cultured for 48 h in a 3D configuration in the central cavity of a chip, before the infusion of the MNP (at a relatively high concentration of 10 mg Fe per mL), with an AMF then applied to the whole chip for a period of 30 minutes. A live/dead assay performed at both 10 and 30 minute timepoints exhibited reductions in cell viability of 20% and 100% respectively. This is a small scale but nonetheless ambitious paper, with an interesting premise and plenty of scope for further experiments. While a viability assay provides important toxicity data, to compete with existing pre-clinical evaluation techniques, exploration into the compatibility of the model with more complex tumour microenvironment evaluation techniques such as immunohistochemistry and RNA sequencing is needed next. This aside, there is clear potential to incorporate MNPs that could enhance the impact on the cells while reducing the required high concentrations, which would otherwise be a barrier to translation. Microfluidic platforms offer a scalable route for optimising MNP design, dosing and MF parameters before progressing to complex and costly *in vivo* studies. Furthermore, the translation of these platforms to patient-derived tumour-on-a-chip models could enable the evaluation of patient-specific responses to hyperthermia, alone or in combination with other therapeutics, thereby supporting the development of more personalized and precise treatment strategies.

Given the extensive MIH research conducted in GBM, the progression to applications in other neurological disorders is perhaps a natural direction (Fig. 2).^10^ Here we can take advantage of the established research foundation to overcome limitations and potentially repurpose existing applicators and biocompatible MNPs for new research areas. Incidence rates of ischemic stroke are predicted to continue growing annually,^39^ and represent a key area of research owing to the time sensitive response required for good health outcomes, with an upper limit of 3–4.5 hours depending on the patient.^40^ Typical treatment requires thrombolysis, using tissue plasminogen activator (tPA), a serine protease which catalyzes the conversion of plasminogen to plasmin, the primary enzyme involved in dissolving blood clots (Fig. 2).^41^ In an insightful study, Cabrera *et al.*^28^ explored targeted MNPs with the aim to induce clot-specific MIH. Initially the study synthesizes and characterizes SPIONs *via* co-precipitation, before coating them with citric acid for biocompatibility, yielding particles with a hydrodynamic size of 154 nm (PDI 0.331). Subsequent labelling with a PAC-1 antibody shows an increase in the size to 180 nm (PDI 0.387) and allows the targeting of activated human platelets, which was successfully demonstrated using a microplate assay.

**Fig. 2:**
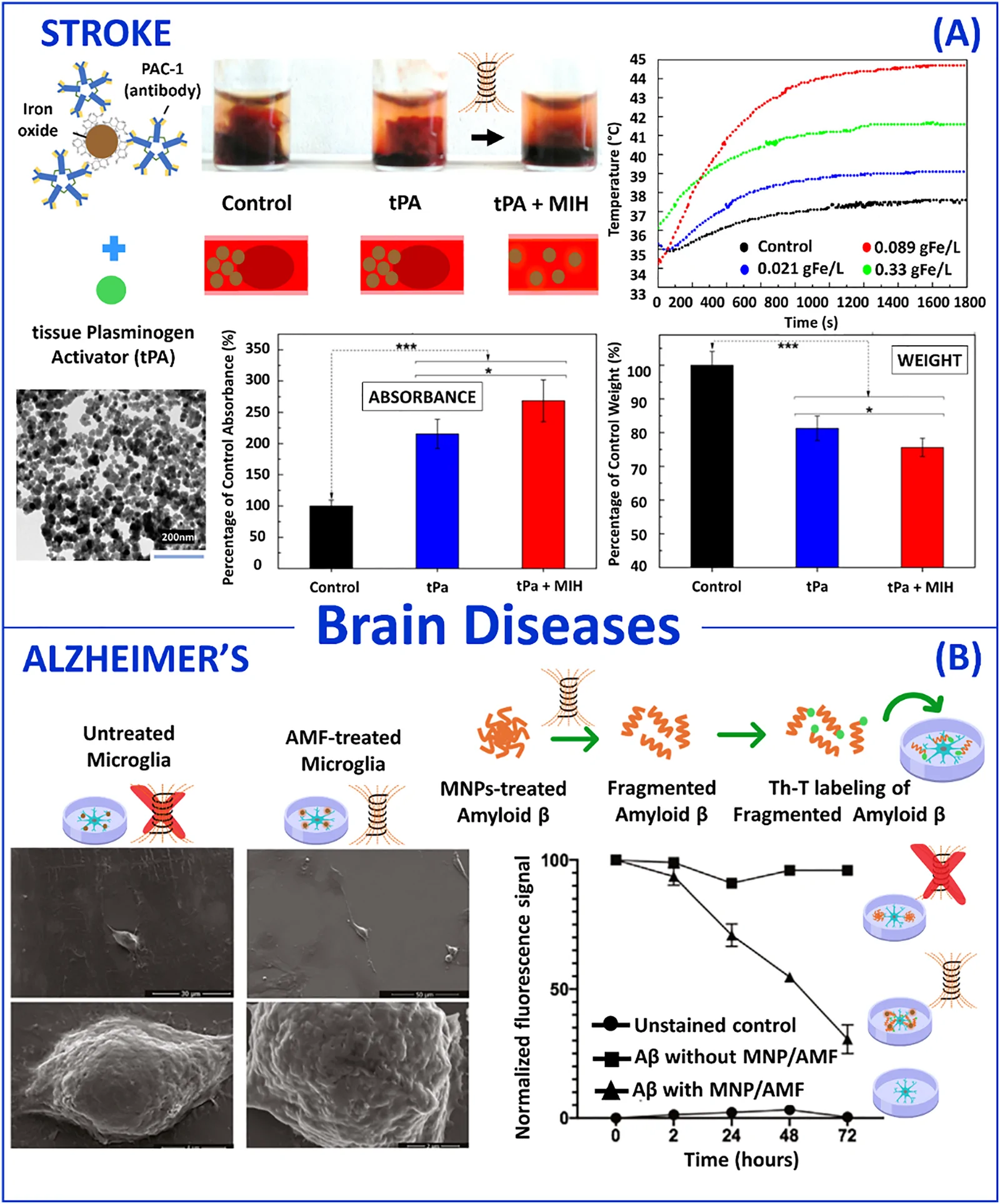
Selected examples of MI in diseases of the brain. (A) depicts clot dissolution in ischemic stroke [adapted/reproduced from ref. 28 under the Creative Commons CC BY 4.0 license,^28^ Copyright 2022].^28^ The study utilises iron oxide NPs targeted using a PAC-1 antibody. Combining these MNPs with an AMF and tPA as a thrombolytic drug showed a reduction in *in vitro* clot weight. Panel (B) shows plaque disruption in Alzheimer's disease [adapted/reproduced from ref. 27 with permission from Elsevier,^27^ Copyright 2021].^27^ Commercial iron oxide NP to induce MIH in a fluorescently labelled plaque model. As the plaques fragment, a reduction in fluorescent signal of ThT dye bound to the amyloid fibrils is observed correlating with plaque fragmentation.

The MNPs were then applied to an *ex vivo* clot model derived from donated human blood, to determine whether MIH could enhance the fibrinolytic activity of tPA, which has been demonstrated to be temperature dependent.^42^ Evidence of the enhanced dissolution of the clots in samples combining tPA with MIH *vs.* tPA alone were provided (Fig. 2A).

This was achieved by specific heating of the clot surface, disrupting red blood cells and thereby increasing the permeability of the clots to tPA, which was quantitatively measured by clot weight and absorbance (Fig. 2A). The authors note that further development of the size and composition of the NPs could take several paths in this area, including targeted release strategies to enhance tPA delivery while simultaneously acting as an MRI contrast agent. In summary, this paper presents MIH as an interesting approach to frontline stroke treatment. Complementing this trajectory, two studies from the Yoon group investigate acute stroke recovery using an *in vivo* rat model to examine magnetothermal brain stimulation.^43,44^ Notably, their findings indicate that focal MIH can activate neural circuits and promote restoration of motor function. When evaluated alongside the work of Cabrera *et al.*, these results suggest hyperthermic approaches under time varying fields with remote activation may be applicable across multiple stages of stroke intervention and merit further translational study.

Although therapeutic delivery to the brain can often be a challenge, MIH has been demonstrated to enhance the permeability of the blood-brain barrier,^45^ and emerging strategies such as nanorobots^46^ could provide a controlled and targeted approach.^47^ A 2024 paper^48^ published in *Small* further supports this idea, presenting an MI-driven strategy for intracranial aneurysm therapy using thermal-responsive magnetic nanorobot collectives. The nanorobots in this instance consist of solvothermally synthesized Fe_3_O_4_ NPs co-encapsulated with thrombin within a phase-change material shell (PCM), forming Fe_3_O_4_-Th@PCM particles (295 nm). Under dynamic torque–force hybrid MFs, the MNPs assemble into swarms and are magnetically navigated into aneurysm sacs. This was comprehensively demonstrated both *in vitro*, and in a rabbit carotid aneurysm model. Localized thrombin release is triggered by MIH, melting the PCM coating at ∼42.5 °C and rapid aneurysm embolization was induced. Collectively, these works suggest that MI strategies could be adapted across the continuum of acute and recovery-phase neurovascular care.

While stroke and aneurysm research represents acute applications of MI-based interventions, long-term neurodegenerative diseases such as Alzheimer's disease offer a very different but no less compelling translational landscape. An accumulation of amyloid plaques is characteristic of this type of dementia, with the main component, β-amyloid peptide, identified as an attractive therapeutic target.^49^ In 2022, Dyne *et al.*^27^ reported amyloid plaque disruption using mild MIH in an *in vitro* model (Fig. 2B). The study used commercially available dextran coated Fe_3_O_4_ NPs and applied an AMF for 15 minutes (frequency 375 kHz; field strength 68 kA m^−1^). Results demonstrated fragmentation of the amyloid plaques, which in turn allowed clearance facilitated by microglial cells (Fig. 2B). This is demonstrated by a fluorescent assay using ThT, (Thioflavin-T), a fluorescent dye which binds amyloid fibrils,^50^ with the results showing a decrease in the fluorescent signal over 72 h for samples treated with MNP/AMF (Fig. 2B). Notably, clearance was also positively correlated with expression of pro-inflammatory genes in microglial cells, including increased expression of heat-shock proteins (HSPs), a class of molecular chaperone associated with proteostasis.^51^ This links with recent research on MIH for deep brain stimulation, which was demonstrated to increase microglial activity and induce HSPs.^52^ Cell viability was also assessed, with no reported toxicity in control microglial cells following the treatment. This emphasises the importance of tailoring AMF application parameters and MNPs to the desired outcome, as previous studies with a focus on tumour-killing have previously reported damage to microglial cells following MIH.^53^*In vivo* investigations on the topic by Jeon and Kim this year delivered similar results, with a reported size decrease of amyloid plaques of 47.6% in a murine model.^54^ These findings also indicated changes in microglial activity and morphology, which complement the *in vitro* findings of the Dyne paper.^27^ Both papers agree that MIH applications in Alzheimer's Disease warrant further exploration. Logical next steps could involve exploration of alternative high-SAR MNP formulations and would require larger scale *in vivo* investigations to assess the longevity of any neuroprotective benefits.

#### 2.1.2 Cardiovascular disease

Not unlike the earlier clot disruption described in the ischaemic stroke research, Weng *et al.*^30^ report the development of spermatozoon-propelled cellular submarines (SPCSs) for targeted thrombolysis and ischemia mitigation (Fig. 3A). The research is aimed at dissolution of thrombi; in this instance, the mesenteric vein that supplies the large intestine was modelled but high applicability to clots in other regions is certainly a consideration. For assembly of the SPCSs, dipyridamole (DIP) was diffused into sperm cells, while a thrombolytic agent, urokinase (Uk), and a hirudin peptide (P6) that serves as an anti-coagulant, were co-assembled into protamine-based NPs (PRO) and electrostatically integrated onto the sperm surface. The resulting SPCS constructs used rheotaxis-driven motion and to achieve effective entry into fibrin clots. Both *in vitro* and *in vivo* mesenteric thrombosis models were used to assess clot penetration. An AMF was used to induce MIH within the clots, based on the inherent iron content of the red blood cells and was aimed at both enhancing the efficacy of the clot penetration and facilitate the release of the therapeutics from the SPCSs. The *in vivo* results reported thrombolytic efficiency of *ca.* 80% as well as increased HSP (HSP70) expression, which play a role in the reduction of vascular inflammation. A similar strategy was employed by Gao *et al.*,^55^ though using a fundamentally different MNP system. This is perhaps more reminiscent of the nanorobotic approach used in the earlier described paper on inter-cranial aneurysm. In this study, a dual frequency-regulated magnetothrombolysis (fRMT) approach was adopted, using nattokinase-loaded magnetic vortex nanorobots (NK-MNRs) with an approximate size of approximately 70 nm.

**Fig. 3:**
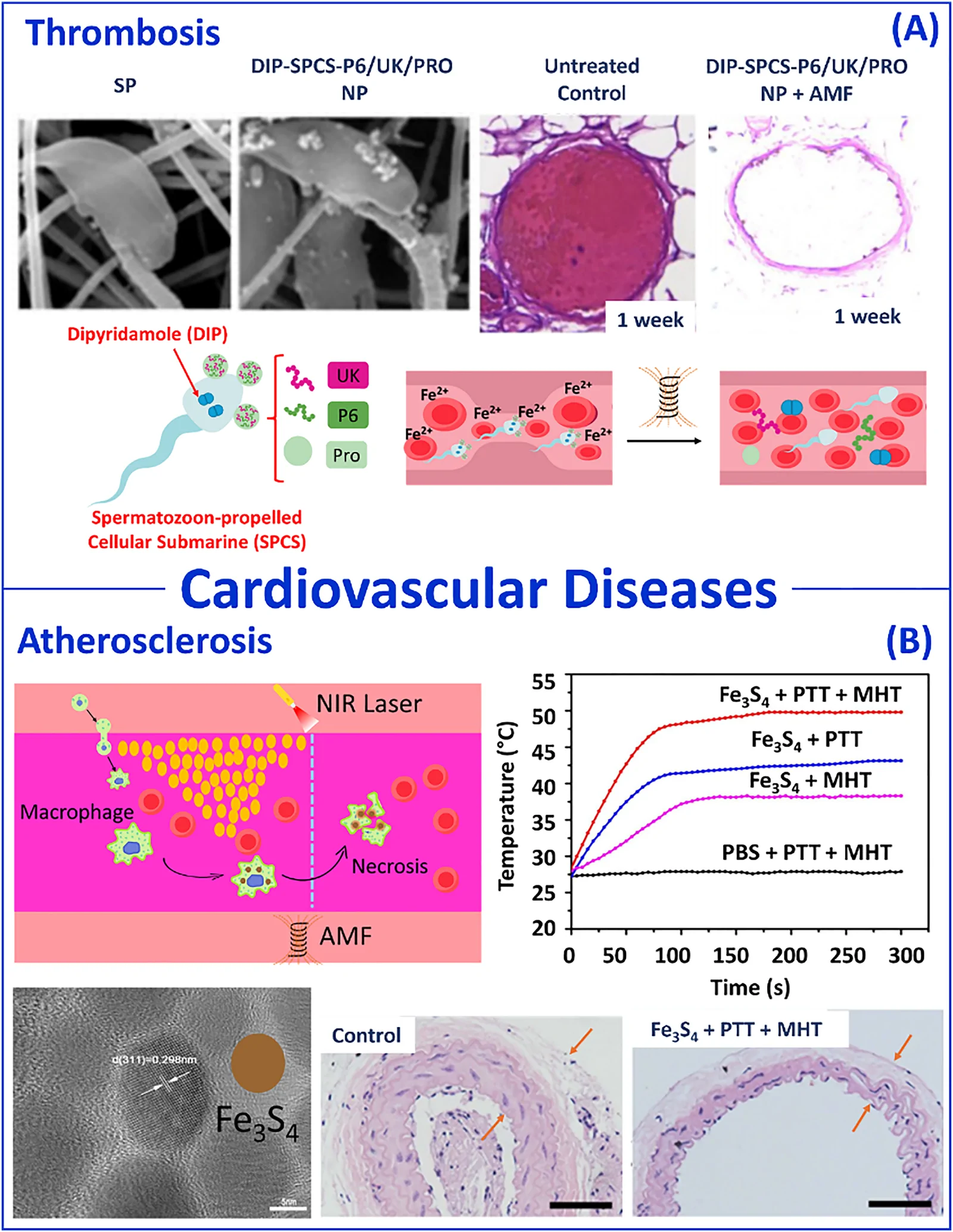
Selected examples of MI in cardiovascular disease. Panel (A) shows the application of spermatozoon-propelled cellular submarines (SPCSs) and MIH in thrombosis treatment [adapted/reproduced from ref. 30 under the Creative Commons CC BY 4.0 license,^30^ Copyright 2024].^30^ SPCSs deliver thrombolytic agents to the clot while MIH was applied to enhance clot permeability and was achieved by inherent iron content in the clot. Panel (B) depicts atherosclerosis, which is characterised by the formation of plaques in arterial walls [adapted/reproduced from ref. 29 under the Creative Commons CC BY 4.0 license,^29^ Copyright 2020].^29^ MIH is applied using Fe_3_S_4_ MNPs in combination with photo-thermal therapy (PTT), resulting in a measurable reduction in inflammation and carotid artery thickness *in vivo* when using PTT and MHT together.

To achieve this, the group first used hydrothermal synthesis to develop Fe_3_O_4_ nanorings (the nanorobots), which were subsequently functionalized with dopamine, and then covalently conjugated *via* glutaraldehyde cross-linking with nattokinase (an enzyme used as an alternative thrombolytic agent). First, a low-frequency rotating magnetic field (MF) of 15 Hz was applied, under which NK-MNRs assembled into chain-like structures, perfect for enhancing clot penetration. MIH (at frequency 375 kHz, and a field amplitude of 26 kA m^−1^) was then used to trigger release of the nattokinase and the enzymatic breakdown of fibrin (fibrinolysis) was observed. *In vivo*, a femoral vein thrombosis model achieved a vascular recanalization rate of 81.0%. Both groups effectively deploy MNP systems to enhance delivery of thrombolytic therapeutics, thereby dissolving clots. Combined with the earlier described stroke research, a real trajectory is emerging in this area, that would most certainly benefit from further exploration.

Pivoting towards disease prevention, induction or suppression of biological pathways using MIH is also seen in novel biomedical applications. In a 2020 paper, Liu *et al.*^29^ explored prevention of atherosclerosis, a cardiovascular disease that presents a significant global disease burden and involves the formation of plaques in the arterial walls.^56^ Macrophage activation has been identified as a key player in the pathogenesis of atherosclerosis, making their elimination a potential avenue to prevent or slow the onset of disease.^57^ The study uses a combination of photo-thermal therapy (PTT) and MIH in combination with Fe_3_S_4_ NPs.^29^ The MNPs were prepared by a hydrothermal synthesis method, reportedly yielding particles of about 13 nm, illustrated by TEM (Fig. 3B). *In vitro* the Fe_3_S_4_ MNPs showed high biocompatibility as well as strong T2-weighted MRI performance, which was confirmed *in vivo*. A combination of MIH and PTT showed efficient ablation of inflammatory macrophages using lower laser power and MF frequency than is typically required. The subsequent *in vivo* atherosclerosis modelling in ApoE knockout mice emphasised that combining PTT (808 nm, 0.3 W cm^−2^) with MIH (595 kHz, 14.5 kA m^−1^) effectively reduced carotid artery inflammation and thickness *vs.* control groups. This is depicted by haematoxylin and eosin-stained images of the carotid artery thickness in a murine model (Fig. 3B). It should be noted however, that this study utilised local delivery of MNPs, injecting directly into the carotid artery, and further experiments would be required to establish efficacy by more typical tail vein routes of administration.

#### 2.1.3 Inflammatory conditions

Expanding on these innovative biomedical uses, a new research direction is represented by the treatment of inflammatory conditions. Inflammatory conditions often have a complex or uncertain pathogenesis, which adds difficulty to treatment avenues available. Endometriosis is a systemic disease characterized by the growth of endometrial-like tissue outside of the uterus which can result in pelvic pain and infertility.^58^ Identifying endometriosis is a tricky business, and for diagnosis, surgery remains the only definitive route. Likewise, treatment options are limited and commonly involve cumbersome excision procedures. Despite affecting up to 10% of women globally, there is no cure.^58^ Difficult problems require creative solutions, and in 2022 a paper by Park *et al.*^31^ presented a comprehensive assessment of MIH for the treatment of endometriosis lesions (Fig. 4A). The team first synthesized hexagonal cobalt-doped iron oxide NPs, adding a PEG-PCL polymer coating and a targeting peptide for biocompatibility and bioavailability respectively. The hexagonal shape is shown to offer superior heating efficacy compared with more traditional, spherical NPs and subsequent *in vitro* assessment showed favourable cell uptake and good heating performance under AMF with increases in temperature of up to 20 °C over 30 minutes (Fig. 4A). The standout results in this paper, however, lie in the *in vivo* study which used endometrial grafts in an athymic nude mouse model. Following IV administration of the NPs (3 mg kg^−1^) a single 20 minute AMF exposure was applied (420 kHz, 26.9 kA m^−1^). Growth curves taken after exposure show complete eradication of the grafts, with a clear trajectory in volume reduction measured over a period of 30 days (Fig. 3A), which is supported by visual eradication of the graft. This is an exciting and comprehensive paper, made yet more appealing by the potential to combine with MRI diagnostics to provide an all-in-one theranostic approach to endometriosis. Translation in this area may prove somewhat more challenging than fields such as brain diseases, where applicators and nanoparticles have an established foundation. For example, localization to the pelvic area may require a similar MI applicator to those recently used in the first clinical trials of pancreatic tumour patients, with applicators produced by Resonant Circuits Limited. Alternatively, the development of endoscope-like applicators^59^ may help to overcome these more practical barriers.

**Fig. 4:**
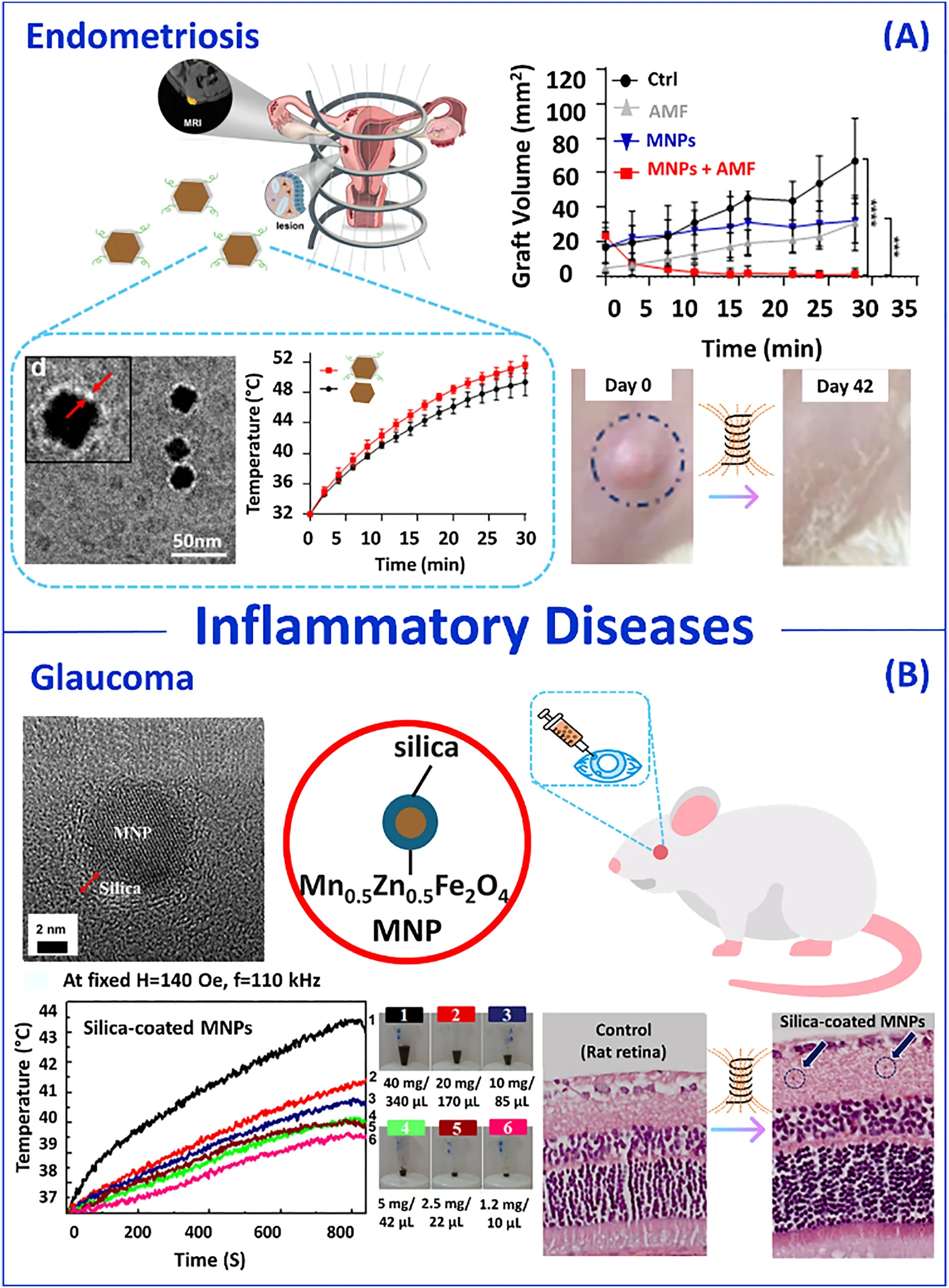
Selected examples of MI Inflammatory disease works. Panel (A) shows the application of hexagonal shaped mixed ferrite MNPs for the treatment of endometriosis lesions [adapted/reproduced from ref. 31 with permission from John Wiley and Sons,^31^ Copyright 2022]. Data depicted show the reduction of graft volume over a period of 30 days.^31^ Panel (B) explores intra-ocular delivery of MNPs for glaucoma, which aims to use MIH to induce heat-shock proteins for therapeutic purposes [adapted/reproduced from ref. 32 with permission from Elsevier,^32^ Copyright 2022].^32^ The MNPs are shown to be effective heat mediators that are able to accumulate in the retina.^32^

Along similar lines, glaucoma represents a further chronic condition in which MI is a promising emergent strategy. Glaucoma is one of the leading causes of blindness worldwide^60^ and has a complex pathogenesis that is not fully understood, with a strong association to inflammatory pathways.^61^ There are several subtypes, all linked to optic nerve damage induced by elevated intraocular pressure. Numerous therapeutic avenues have been explored including prostaglandin analogues, adrenergic agonists and the aforementioned thermal-related proteins, the HSPs. It is this final target that piqued the interest of a group at the National University of Singapore. In a 2011 paper, Jeun *et al.* sought to induce production of HSP using magnetic hyperthermia (Fig. 4).^32^

To successfully achieve the required *in vivo* temperature range of 39–41 °C, MNPs used for HSP induction need to exhibit a high SAR rate alongside high biocompatibility. HSPs hold controversial status in glaucoma research, with some papers indicating they are neuroprotective, while other results suggest quite the contrary. Setting aside efficacy of the chosen target, the group's approach involved first synthesising Fe_3_O_4_, NiFe_2_O_4_, Ni_0.55_Zn_0.5_Fe_2_O_4_ and Mn_0.5_Zn_0.5_Fe_2_O_4_ MNPs using a high temperature thermal decomposition method, with a goal to compare magnetic properties both with and without a silica coating. The spherical Mn_0.5_Zn_0.5_Fe_2_O_4_ NPs outperformed the other candidates, showing a SAR of 256.4 W g^−1^ in an agar solution. In addition, small mean particle size of only 5.5 nm was determined, which can be both advantageous for cell entry while also limiting the blood half-life of the NP. *In vivo*, effective delivery was enhanced by direct injection into the vitreous body, an unusual approach, but allowing diffusion of the Mn_0.5_Zn_0.5_Fe_2_O_4_ MNPs directly to the retina (Fig. 4B). A further paper by the group confirms direct induction of HSP72 in a cellular model.^62^ Future progress in this area is likely to depend on optimising high-SAR, biocompatible MNP formulations that can be safely delivered to the retina.

Overall, the studies in this section show how MI is driving innovation in disease settings where conventional approaches are limited or ineffective. Biological context strongly informs the therapeutic outcome, with each target imposing different constraints on delivery and heating efficacy. Reflecting this, we note the wide range of delivery strategies presented, with some truly innovative approaches. NP design is a key control variable and central area for progression in almost all cases, as size, composition, coating and shape influence both heating performance and biocompatibility. As these elements become more closely optimised for specific applications, it's clear there will be a shift from exploratory studies toward more focused translational applications.

### 2.2 Biomedical engineering

Continuing the theme of translational research, we now step into biomedical engineering. As an overview, in Fig. 5, with schematic cartoons, we show a broad spectrum of activities are encompassed within biomedical engineering processes. Applications range from nanowarming strategies for organ and oocyte thawing procedures,^63–70^ to implant-associated interventions such as in-stent restenosis treatment^71^ and prosthesis removal,^72^ and to advanced cell engineering approaches enabling cell's ion channel modulation^73^ or transient membrane permeabilization^74^ to enhance nanoparticle and siRNA delivery or pluripotent stem cell differentiation.^75^ A substantial domain further addresses biofilm eradication for infection control in bone regeneration, orthopaedic and dental implants, caries treatment, and the decontamination of diverse biomedical surfaces.

**Fig. 5:**
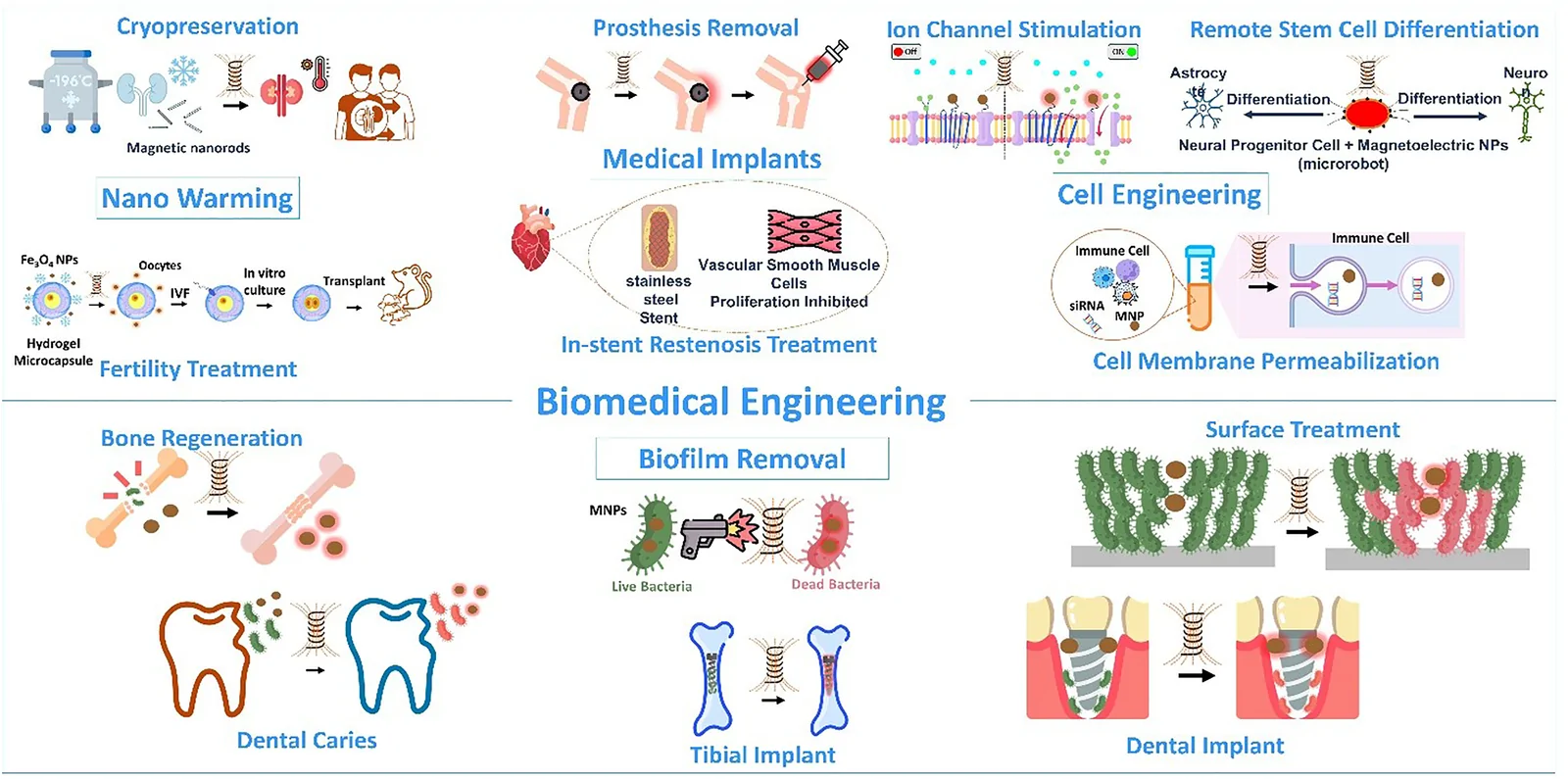
MI in biomedical engineering applications. Clockwise from top left: Nanowarming applications in organ preservation^68^ and fertility treatment [adapted/reproduced from ref. 70 under the Creative Commons CC BY 4.0 license,^70^ Copyright 2022].^70^ Medical implants such as liquid metal implants^72^ and stent hyperthermia.^71^ Cell engineering *via* membrane permeabilization^74^ ion channel stimulation^73^ and stem cell differentiation^75^ [adapted/reproduced from ref. 75 with permission from Springer Nature, Copyright 2026]. Biofilm removal with multiple applications across surface treatment, dental^76^ and bone implants,^77^ dental caries^78^ and bone regeneration.^79^ Selected icons and graphical elements used were obtained from Flaticon (https://www.flaticon.com) and adapted for use in this figure.

#### 2.2.1 Nanowarming

MI is emerging as a means to facilitate advances in areas such as surgery and transplantation.^63–70^ Transplantation remains one of the most effective ways to treat organ failure but is continually challenged by shortages in viable donor organs. Strategies in recent years therefore have aimed to improve preservation and rejuvenation, in a bid to make a greater number of viable organs for transplantation.^80^ Devitrification is a significant obstacle for cryopreserved organ samples, where ineffective thawing leads to the formation of ice crystals, which can disrupt tissue structure and even render the organ unsuitable altogether for transplantation.^81^ Nanowarming, which uses MNPs and an AMF, can offer a rapid and more uniform alternative to traditional thawing processes. In this strategy, MNPs are perfused throughout the organ during preservation processes. Thawing is achieved by subsequent exposure to an AMF, providing a more uniform dispersion of heat throughout the organ and hence limiting potential damage to the tissues. This area has perhaps a greater volume of studies than others we have introduced, with early work exploring iron oxide NPs in mesenchymal stem cells.^82^ Advancing this, a study published^83^ in *Science Translational Medicine* in 2017 demonstrated that cardiac tissue can be successfully cryopreserved and subsequently rewarmed by perfusing MNPs, enabling rapid and uniform thermal recovery. In the study, nanowarming was applied to porcine artery models (20 kA m^−1^, 360 kHz) using iron oxide NPs with a mesoporous silica coating.^83^ This demonstrated high potential for application to whole organs, and indeed this was achieved in a 2021 study investigating the nanowarming of whole hearts with a heating rate of 321 °C min^−1^.^63^ In an efficacious further expansion, Liu *et al.* recently built on this work using Fe_3_O_4_@SiO_2_ nanorods in porcine artery models.^84^ Here, the use of alternative MNP morphology proves advantageous, and alignment of the nanorods under a static magnetic field prior to AMF application ensured more uniform heating and safer rewarming of cryopreserved tissues.

Nanowarming has proven equally practical in other organ systems including pancreatic islets (50 °C min^−1^ heating rate),^64^ cartilage (76.8 °C min^−1^ heating rate),^65^ liver ( 61 °C min^−1^ heating rate),^66^ and kidneys (63.7 °C min^−1^ heating rate).^67,68^ Notably, Han *et al.* give an interesting insight into the potential of the technology in more long-term cryopreservation, reporting that vitrified kidneys could be cryogenically stored up to 100 days and subsequently recovered by nanowarming. An *in vivo* rat model delivered successful transplantation in addition to restored full renal function in nephrectomised recipients. To achieve this, silica-coated iron oxide NPs were perfused through the renal vasculature together with a cryoprotective agent (VMP). Kidneys were cooled and stored at −150 °C for up to 100 days before nanowarming at a mean rate of 63.7 °C min^−1^. Application of an AMF (63 kA m^−1^, 180 kHz), produced consistent internal heating that prevented problematic crystallization.

Outside of direct organ transplantation, nanowarming also has applicability in fertility treatment (Fig. 6A). A 2022 study in *Nature Communications* explored the cryopreservation of isolated early-stage ovarian follicles in a hydrogel, and subsequent nanowarming.^70^ The follicles were isolated from a mouse model, before encapsulation in 1% alginate hydrogel microcapsules and preserved using a penetrating cryoprotectant (1.5 M EG/PROH with trehalose). During rewarming, Fe_3_O_4_ MNPs and graphene oxide NPs located outside the hydrogel generated rapid heating *via* combined MIH and laser-induced application, achieving warming rates up to ∼93 °C s^−1^. The approach significantly improved follicle survival and preserved follicular structure. Not only that, but upon thawing, *in vitro* fertilisation and embryo transfer, vitrified follicles successfully produced healthy first- and second-generation offspring. Related work in 2023 explored ovarian tissue cryopreservation in a sheep model,^69^ exemplifying scalability to large animal models. The group used iron oxide MNPs added directly to the cryopreservation solution and an AMF (26 kA m^−1^, 400 kHz) applied for 1 minute to achieve subsequent thawing. Results indicated improved follicular development and gene expression compared to standard cryopreservation methods. From a translation perspective, these investigations could offer alternative cryopreservation strategies in some cancer patients, where fertility preservation is offered before a course of treatment. This adds another layer of appeal to the future of nanowarming, given that developed NPs or systems have the potential to be utilised across multiple organ systems and sets the stage for other aspects of biomedicine where cryopreservation is routinely used.

**Fig. 6:**
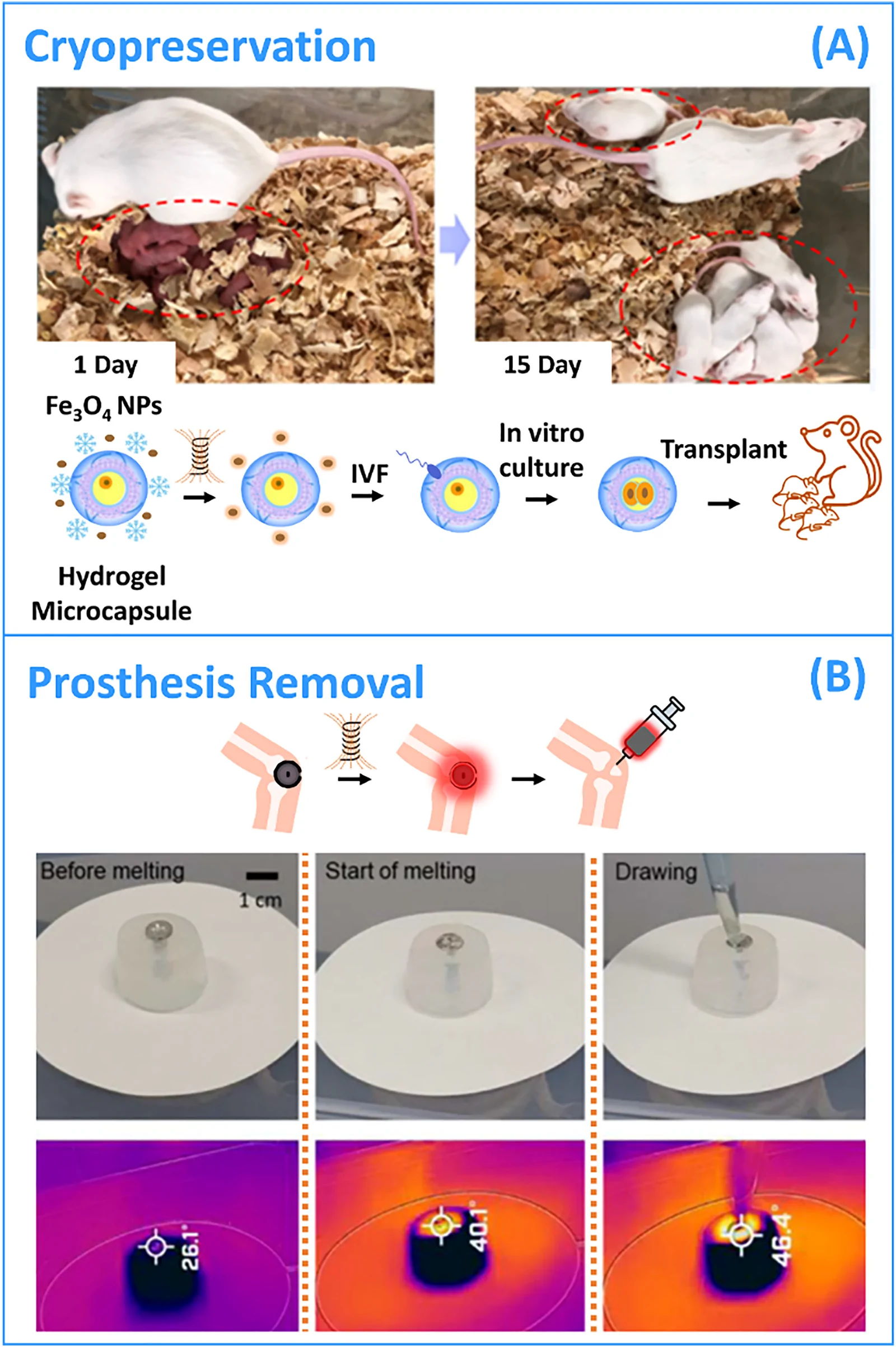
MI in cryopreservation and prosthesis removal. Panel (A) describes nano warming used for fertility treatment in a mouse model, producing healthy 1st and 2nd generation offspring [adapted/reproduced from ref. 70 under the Creative Commons CC BY 4.0 license,^70^ Copyright 2022].^70^ Panel (B) depicts prosthesis removal, whereby a liquid metal is used to form a medical implant [adapted/reproduced from ref. 72 with permission from Elsevier,^72^ Copyright 2022].^72^ At the time of removal, an AMF can be applied to melt the implant, allowing removal *via* syringe.

#### 2.2.2 Medical implants

Through the previous sections, we have encountered vascular applications of MIH, both in preventative and treatment avenues. The following study again follows this trend, targeting blockages or narrowing of the coronary artery following angioplasty procedures (termed restenosis), but with a markedly different approach. Li *et al.*^71^ evaluate the feasibility of a stent made of austenitic stainless steel under exposure to a MF in an *in vitro* rabbit model for restenosis. The root cause of restenosis is thought to be linked to abnormal proliferation and migration of vascular smooth muscle cells (VSMCs), which in turn causes narrowing of the coronary artery. To evaluate the efficacy of the approach, rabbit VSMCs were co-cultured with commercial 316L stainless steel stents and subjected to an AMF (300 kHz) for 10 min at defined temperatures. This stent material, rather than heat *via* magnetic hysteresis losses produced induction heating *via* eddy current generation dependent on field strength and stent orientation. When temperatures were achieved ≥47 °C, the results showed significantly inhibited cell proliferation and induction of apoptosis of the VSMCs, while temperatures around 43 °C produced a minimal effect.

Given the eddy current generation – harnessed for benefit in the previously outlined study – patients with pre-existing metallic medical implants can cause safety concerns when exposed to MFs. Collateral heating could feasibly damage healthy peripheral tissues or alter implant integrity. A computational study conducted by Rubia-Rodriguez *et al.* used *in silico* modeling to quantify temperature elevations across a range of implant geometries and materials during MIH therapy.^85^ The work identified key factors such as implant size, shape, orientation relative to the MF, and field frequency as critical determinants of thermal response. Their findings demonstrated that eddy current-induced heating is highly dependent on both the electrical conductivity and magnetic permeability of the implant material, with highly conductive metals posing a greater risk of excessive heating. Thus, thorough patient evaluation and detailed assessment of implanted devices are crucial prior to MIH with real-time thermal monitoring also offering a possible solution.

An altogether different way forwards however, is offered by Abbasi *et al.*, who suggest biomedical implants such as screws, could instead themselves be composed of low-melting liquid metal alloys.^72^ Liquid metals are gaining recognition for their diverse biomedical applications.^86,87^ In the Abbasi study, two eutectic alloys were investigated in the research; Field's metal (Bi–In–Sn) and a Zn-doped variant (Bi–In–Sn–Zn). The materials are designed to remain solid at body temperature, but exposure to an AMF would generate induction heating by eddy current, melting the implant and allowing the material to be removed without invasive surgery, using a local induction heater and a syringe (Fig. 6B). *In vitro* biocompatibility was assessed using mouse embryonic fibroblast (MEF) cells, with Field's metal exhibiting better survivability. More complex proof-of concept was achieved using 3% agar gel and swine tissue models, where the liquid metal implants were melted and removed by suction, with surface temperatures remaining below ∼60 °C and the induction coil temperature staying below 45 °C, shown by thermal images depicted in Fig. 6B. While modelled for tumour heating, it is also relevant to point out that this method has also been explored for implantable electrodes.^88^ Gallium–indium liquid metal was combined with Fe_3_O_4_ MNPs to create a composite whose mechanical modulus could be reversibly increased under an external MF. The electrode remained stiff during implantation and softened *in situ.* Under an AMF, the implanted electrode generated localized heat in a mouse tumour model for triggering therapeutic effect. To compete with pre-existing medical implants, exploration into longevity and long-term safety would unquestionably be required.

#### 2.2.3 Biofilm treatment

Unsurprisingly, there is significant overlap between medical implant and biofilm applications of MI, due to the risk of infection following surgical procedures. Much like the research explored in the medical implant literature, some works explore heating of the implant itself by eddy current rather than using MNPs. Some studies propose application of an intermittent AMF in conjunction with antibiotics as a proposed treatment strategy for eradicating biofilms on metallic implants.^89,90^ Shaikh *et al.*,^89^ modelled this approach *in vivo* whereby stainless-steel beads colonized with *S. aureus* or *P. aeruginosa* biofilms were implanted into mouse muscle. Exposure to repeated intermittent AMF heating cycles raised implant surface temperatures using brief pulses (5–300 s at 55 °C to 75 °C), delivered once or twice daily over four days. AMF combined with the antibiotics produced more than a one order of magnitude (>1-log) additional biofilm reduction compared with antibiotic or AMF alone, with efficacy increasing at higher target temperatures. Tissue damage was contained in an area approximately smaller than 1 mm from the implant surface, supporting the safety of this approach for treating implant-associated infections. Further computational research has confirmed this is an achievable margin,^91^ which is an important consideration in the progression of the field. Recent work in an ovine model corroborates these findings, showing reproducibility in larger animals.^77^ Titanium or cobalt-chrome plates were inoculated with *S. aureus* and implanted onto sheep tibia. As in the previous study, treatment comprised AMF exposure (9 kA m^−1^, 213.8 kHz, 20–30 s per exposure) with antibiotics, with a target temperature of 75 °C at the surface of the metal plate, with significant biofilm reduction seen in this combination group compared to the control. The novelty of these works lies in the idea of exploiting the heat dissipation of the implant material itself (likely eddy current mediated) to generate heat under magnetic induction (MI).

The concept of MIH for bacterial killing is not a new one.^92,93^ Naturally therefore, there is also a research pipeline devoted to developing MNPs for biofilm disruption, with a number of papers exploring different NP compositions such as ferrites.^94^ Liu *et al.*^95^ solvothermally synthesised spherical Fe_3_O_4_@PEI NPs (74.16 nm) before testing them in *S. aureus* and *E. coli* biofilm models with AMF application. The functionalisation with polyethyleneimine (PEI) was considered of particular importance by the group, as it gave the MNPs a positive surface charge which they theorise increases interaction with the biofilm. Thus, the local thermal and mechanical stress would be increased, which could also facilitate the generation of reactive oxygen species (ROS). Overall, the Fe_3_O_4_@PEI NPs exhibited excellent antibacterial and antibiofilm activity when exposed to an AMF, compared to without AMF. Further research could aim to explore not only high SAR MNP formulations, but also different cationic surface molecules to further enhance biofilm penetration. Unsurprisingly, other works have also explored iron oxide compositions but changed the NP surface coating. A 2023 paper^96^ published in *Small* looked at hyaluronic acid-coated ferrite (HA@MnFe_2_O_4_) with AMF (1.35 kA m^−1^ for 10 min) exposure for *S. aureus* biofilms. This particular coating was chosen, based on research demonstrating that some bacterial strains can degrade hyaluronic acid (HA) into anti-inflammatory disaccharides. The aim therefore would be to encourage healing through both the biofilm disruption and, anti-inflammatory regulation at the site of infection. In addition to this, the study explores the impact of MIH on quorum sensing, which is chemical signalling mechanism that informs bacterial gene expression based on population density, and is considered important in the formation of biofilms. Interestingly, the results reported not only the anti-biofilm effects seen in other systems, but also downregulation of the quorum sensing pathways, which can decrease bacterial virulence and metabolic activity. As with the biofilm implant investigations, some groups have explored the combined application of MNPs, AMFs and antibiotic therapy. One study, for example describes a controlled release strategy in *S. aureus* biofilms, seeking as with the other studies, to address the antibiotic resistance often commonly seen in this strain.^97^ Fe_3_O_4_@chitosan NPs were functionalized with antibiotic (vancomycin) *via* a temperature-sensitive azo linker (4,4′-azobis(4-cyano valeric acid)) to form a thermo-labile bond between the MNPs and the antibiotic molecule that can break by temperature elevation with approximately 40% drug release seen at 57 °C which increases to 90% at 80 °C. A static magnetic field (∼2000 Gauss) was used to drive entry into the model biofilm, which proves the ‘guided’ aspect of the delivery. AMF stimulation (*H*_f_ = 5.82 × 10^8^ A m^−1^ s^−1^, 10 min per cycle) was then used for triggered vancomycin release. Biofilms were shown to have been substantially disrupted forming an ‘antibacterial zone’ in the applied region, underlining the success of combining with therapeutics. Other studies have adopted alternative approaches to therapeutic integration, including an interesting paper exploring nanocomposites with chemotherapy in biofilms.^98^ Finally, a recent paper^99^ also explored the combination with therapeutics, designing a hydrogel-based dressing for diabetic wounds, which are prone to chronic infection and struggle to heal. The authors combined tannic acid thermoresponsive polymers and Fe_3_O_4_ MNPs as heating agents to form the basis of the hydrogel. Application of an AMF at differing intensities provided differing effects of first sterilization and subsequently wound closure. This area truly has a diverse range of approaches, which could facilitate combinations of the various techniques to enhance efficacy.

Just as in surgical procedures, biofilm formation often presents an issue in dental applications. In this discipline, MIH is emerging as a novel adjunctive approach, leveraging MNPs activated by AMF to generate localized heating for various therapeutic purposes such as root canal treatment and dental infections.^100^ In 2022, Dasgupta *et al.*^76^ demonstrated the therapeutic potential of hyperthermia-based magnetic nanobots, in which 15 nm pure iron particles embedded within a silica body conferred magnetic properties to the helical nanostructure. Their results confirmed that surface heating of the nanobots under an AMF (40 kA m^−1^, 15 min) was sufficient to eradicate *Enterococcus. faecalis*, a highly resistant bacterium and the primary cause of endodontic reinfection. Importantly, the study revealed that magnetically actuated nanobots could navigate the depths of dentinal tubules by periodically reversing the rotational direction of the MF (5 kHz, 2.4 kA m^−1^, every ∼180 s), a level of control not achievable with current clinical methods, highlighting the considerable potential of magnetic nanobots and MIH in dental applications. The ability to externally guide superparamagnetic agents to target sites and convert AMF into localized heat offers a promising adjunctive strategy for eradicating odontogenic biofilms and may further broaden the clinical potential of MIH across a range of dental treatments. In another study, Yanru Chen *et al.*^78^ showed strong magnetic-assisted antibacterial effects which holds potential for dental materials aimed at preventing caries. They developed and evaluated a novel antibacterial magnetic-based nanoplatform, designed to inhibit oral biofilms associated with dental caries. The MNPs consist of a magnetite (Fe_3_O_4_) core coated with silica (SiO_2_) shell and chemically grafted with the cationic antibacterial monomer dimethylaminododecyl methacrylate; resulting in uniform spherical particles with sizes of ∼200–300 nm. Characterization *via* XRD and VSM confirmed preservation of the Fe_3_O_4_ superparamagnetic crystal structure and a saturation magnetization of ∼36 emu g^−1^. Under exposure to an external MF (field parameters not specified), the nanomaterial significantly disrupted *Streptococcus* mutans biofilm, reduced lactic acid production, and modulated the microbial composition of saliva-derived biofilms, decreasing caries-associated genera such as *Streptococcus*, *Veillonella*, and *Neisseria*. Although the MF parameters are not numerically specified in the manuscript, the results showed that biofilm inhibition occurs only when the NPs are activated by the applied MF.

#### 2.2.4 Bone regeneration

Bone regeneration, particularly in the context of large or critical-sized defects, is another promising application area for MF stimulation, linked again intrinsically with biofilm disruption. Infectious bone defects are a major orthopedic challenge, driven by chronic infection and progressive bone loss that can result in limb impairment. Seeking to address both the challenges of infection control and impaired bone regeneration in this area, a paper published in 2024 looked at the potential of magneto-piezoelectric iron-doped barium titanate (BFTO) NPs.^79^ The BTFO NPs were synthesised and then loaded with curcumin (an anti-inflammatory agent); within a coating obtained with engineered mesenchymal stem cell membranes (EMM) modified with γ3 peptide. Exposure to an AMF (310 kHz and 5.3 kA m^−1^ for 120–180 s) demonstrated enhanced biofilm disruption in the presence of the BTFO NPs-based composites. However, the approach has an additional target, specifically the elucidation of immune responses in Icam1+ macrophages, which have been shown to have a key regulation role in bone defect repair. This ancillary effect is driven by exposure to low-intensity pulsed ultrasound. To demonstrate relevant application, BTFO NPs were integrated into quaternized chitosan and tricalcium phosphate to create a bioink for three-dimensional (3D) printing anti-infection bone repair scaffolds. Testing in a rat model showed that the scaffolds markedly enhanced repair of infected bone defects while avoiding damage to adjacent tissues. The results demonstrate that the material effectively supports bone healing under infectious conditions. Combined with the selective macrophage targeting, this showcases a dual-action approach to both suppress infection and stimulate bone regeneration.

More broadly, in 2022, Zhao *et al.* published a comprehensive review, examining how electromagnetic fields (EMFs) can enhance outcomes in bone tissue engineering strategies that typically rely on stem cells seeded onto scaffolds.^101^ The review discusses the use of both pulsed EMF and sinusoidal EMF, with the latter shown to improve cell adhesion, proliferation, and osteogenic differentiation, particularly in mesenchymal stem cells. EMF exposure was also associated with upregulation of pro-angiogenic factors such as vascular endothelial growth factor, supporting neovascularisation in regenerating tissue. Perhaps most compelling is the reported modulation of the immune microenvironment; EMFs promoted M2 macrophage polarization, which is considered beneficial for tissue repair. These effects echo the findings of the *ACS Nano* paper,^79^ and interestingly, work into tissue regeneration, where AMF and MNP application has been used to tune reactive oxygen species generation.^102^ While clinical use of pulsed EMFs is already FDA-approved for fracture healing and spinal fusion, the integration of EMF stimulation into complex bone tissue engineering systems remains at an early stage. The potential of conductive polymers as MI-responsive materials could be harnessed here,^103^ as well as emerging enhanced delivery strategies such as micro- and nanorobots that have been explored for other medical applications and specifically antibiotics.^104^

#### 2.2.5 Cell engineering

In many of the biomedical MIH applications we have analysed, there is a trend towards targeting the activation or indeed suppression of biological pathways. It is little wonder therefore that this translates to research on the cellular level.^105^ Specifically, remote activation of transient receptor potential vanilloid 1 (TRPV1) cation channels has been investigated, based on the temperature sensitive nature of the channel.^73^ The work reports a non-invasive method for thermal controlling neuronal activity deep within tissue, using superparamagnetic MnFe_2_O_4_ MNPs (∼6 nm). The TRPV1 target is a (Ca^2+^) channel is widely expressed in neuronal cells and implicated in perception of pain and heat. The synthesised ferrite MNPs were functionalized with streptavidin and targeted to specific proteins on the plasma membrane of cells expressing the TRPV1 ion channel. Exposure to an AMF (40 MHz, 0.7–1 kA m^−1^) induced highly localized heating at the plasma membrane, manipulating the ion channel to control calcium influx and henceforth neuronal stimulation. The chosen model was roundworm (*C elegans*) and had the interesting effect of inducing thermal avoidance behaviour. Targeting of the same TRPV1 ion channel in mice has been demonstrated previously by Chen *et al.* with the added prospect of longer-term stimulation, as the Fe_3_O_4_ MNPs they used showed persistent presence in the brain for up to a month post-administration.^106^ Further corroboration on TRPV1 stimulation for behavioral impact was again demonstrated in mice, with control of motor function.^107^

In addition to ion channel manipulation, approaches have also explored membrane disruption for cell delivery using AMF in conjunction with MNPs. A study by Idiago-López *et al.* introduces a membrane-localized MIH strategy for the delivery of cell-impermeant probes.^74^ Spherical iron oxide MNPs (∼13 nm) were functionalized with strained alkynes to enable a covalent interaction directly with the cell membrane. Upon exposure to an AMF (425 kHz, 16.9 kA m^−1^, 30 min), the membrane-bound MNPs act as nanoscale heat sources that disrupt the integrity of the plasma membrane. The team used this approach to enable transfection of cells with a small interfering RNA (siRNA), highlighting future potential as a novel intracellular delivery mechanism. This could have meaningful applications in drug and MNP delivery, or indeed hybrid compositions. In the same direction, functional repurposing of MIH is expanding toward immune cell engineering. In a recent work by the Pellegrino group, mild hyperthermia (below 41 °C) and clinically compatible AMF conditions are shown to enhance MNPs uptake by immune cells such as natural killer (NK) cells and T-cells without impairing their physiological functionality, including the ability of immune cells to recognize and kill tumour cells (Fig. 7A).^108^ The authors also showed that different MNP formulations, varying in composition (iron oxide or zinc ferrite nanoparticles) and surface coating (commercial or modified amphiphilic polymers),^109–111^ can be successfully employed, provided that the colloidal stability of the MNPs is maintained in the immune cell culture medium.

**Fig. 7:**
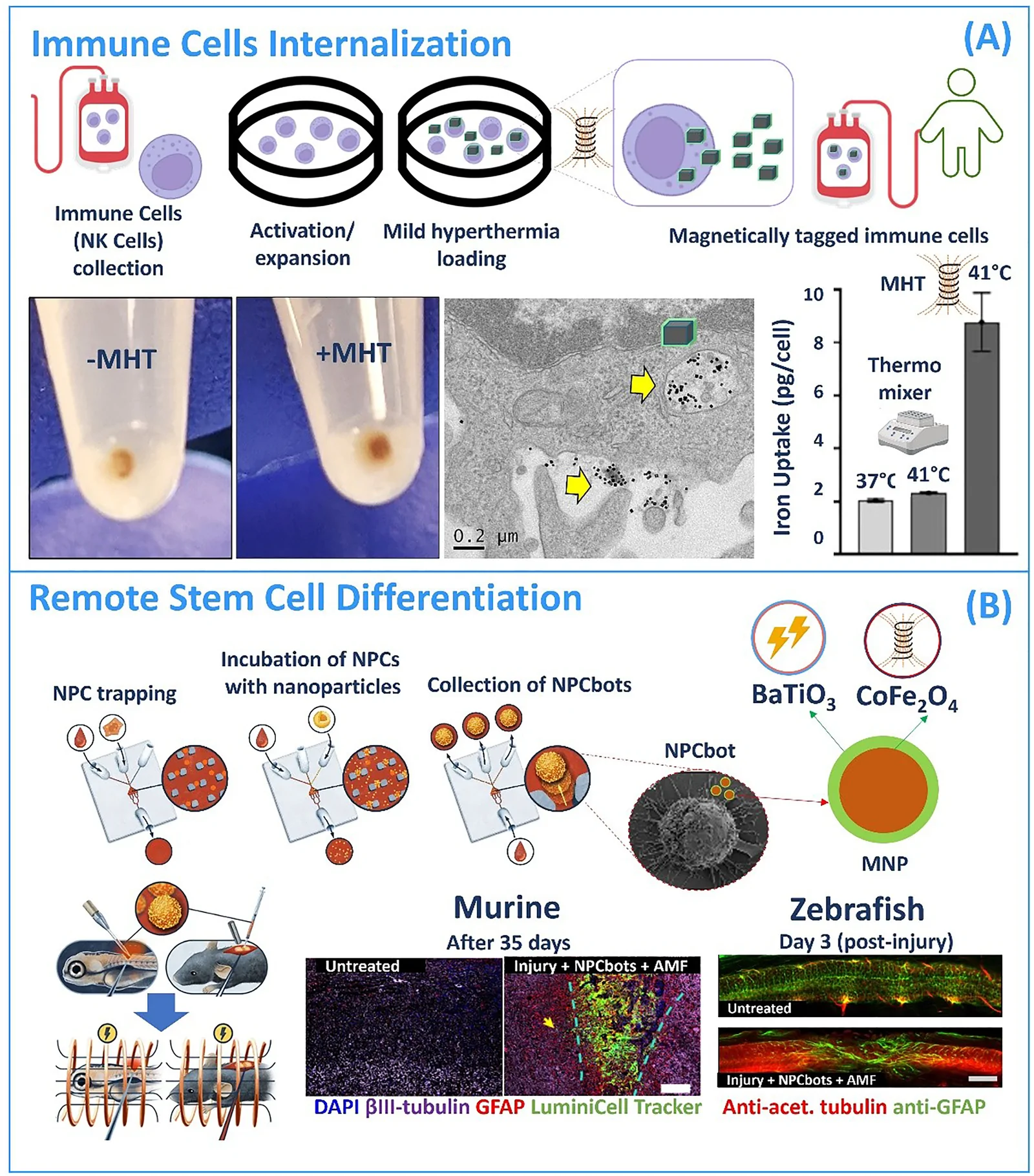
Uses of MIH in cell engineering. (A) Functional repurposing of AMF (at 41 °C) to enhance internalization of MNPs by immune cells as shown by brownish color of cell pellet and presence of MNPs in the endosomal vesicles of the cells in TEM image [adapted/reproduced from ref. 108 under the Creative Commons CC BY 4.0 license,^108^ Copyright 2026].^108^ (B) MIH for spinal cord repair using nanobots,^75^ a biohybrid microrobot composed of human iPSC-derived neural progenitor cells (NPCs) coated with magnetoelectric CoFe_2_O_4_–BaTiO_3_ (CFO–BTO) core–shell NPs, demonstrating AMF-induced stem-cell differentiation into neuronal and astrocytic lineages as indicated by βIII-tubulin and GFAP co-localization. In murine models, they enhanced integration with host astrocytes and reduced lesion size. In Zebrafish they integrate into neuronal (α-tubulin-positive) and astrocytic (GFAP-positive) cells producing significant improvements in motor function [adapted/reproduced from ref. 75 with permission from Springer Nature, Copyright 2026].^75^

The simplicity and speed of this MIH protocol was suggested as a key strength of the method, allowing ready integration into GMP-compliant adoptive cell therapy workflows to generate magnetically labelled immune cells for image-guided adoptive therapies.

This year, a landmark example of wireless MI induced stem cell differentiation was reported in the context of spinal cord injury repair.^75^ The study employed a scalable microrobots (NPCbots) system consisting of human induced pluripotent stem cell-derived neural progenitor cells integrated with magnetoelectric core-shell NPs, specifically made of cobalt ferrite core and barium titanate shell (Fig. 7B). These nanoparticles were employed to remotely deliver mechanical stimulation and promote regenerative cell responses *via* wireless magnetoelectric stimulation. Exposure to an AMF (16 kA m^−1^, 1.18 kHz) prompted magneto electric stimulation of magnetic-piezo electric NPs, which in turn, activate neuronal and astrocytic differentiation confirmed by increase in neuronal and astrocytic marker expression (Fig. 7B). In a zebrafish spinal cord injury model, injected NPCbots were stimulated by AMF over a period of five days and demonstrated enhanced graft integration, effective neural repair, and most interestingly, a near-complete recovery of swimming and exploratory behaviours. Combined with their potential ability to be magnetically navigated *in vivo*, these findings highlight the considerable potential of microrobot-assisted regenerative therapies.

### 2.3 Molecular diagnostics

#### 2.3.1 Sensing

In recent years, fueled in part by the COVID-19 pandemic, the need for fast and accurate *in vitro* diagnostic tools to identify antigens, pathogens or biomarkers has significantly increased. Within this broader push for rapid diagnostics, MNP–based sensing has emerged as a particularly powerful platform. In this scenario, the use of NPs and especially MNPs has increased, owing to their ability to produce quantifiable and readily deployable signals.^114^ Capitalising on this, researchers have focused on exploiting the inherent magnetic relaxation features of MNPs. To this aim, Brownian relaxation MNPs are the most suitable, since their relaxation dynamics significantly change in response to binding events when NPs are exposed to a time-varying MF, producing detectable signal shifts that can be rapidly measured using various techniques. Sanz-de Diego *et al.* for instance, used MNPs bio-conjugated with an engineered recognition peptide as a receptor and AC magnetometry to identify the presence of monomeric or dimeric verde fluorescent protein (VFP) as analyte in liquid (Fig. 8A).^115^ This methodology is based on the variation (reduction) of the AC magnetic hysteresis area obtained under AMF in bioconjugated MNPs upon specific interaction with their specific receptor. In a further interesting study, Rösch *et al.* developed an MNP-based assay for the detection of the entire SARS-CoV-2 genome without PCR amplification.^116^ The method employs a target-triggered magnetic signal amplification circuit (MAC) based on toehold-mediated DNA strand displacement. In this system, MNPs are initially assembled into clusters through DNA linkers, resulting in slow Brownian relaxation and a weak magnetic particle spectroscopy (MPS) signal. Upon target recognition, a strand displacement cascade is initiated, releasing DNA amplifier strands that progressively disrupt the DNA-mediated connections between the MNPs. As the clusters disassemble into individual MNPs, their Brownian relaxation becomes faster, leading to an increase in the MPS harmonic signal. The extent of declustering is quantified by monitoring the ratio between the 5th and 3rd harmonic amplitudes. This technique can be used instead of PCR and is based on the change of magnetic signal when MNPs are single or aggregated by measuring the closing of the magnetic hysteresis cycles with magnetic particle spectroscopy.

**Fig. 8:**
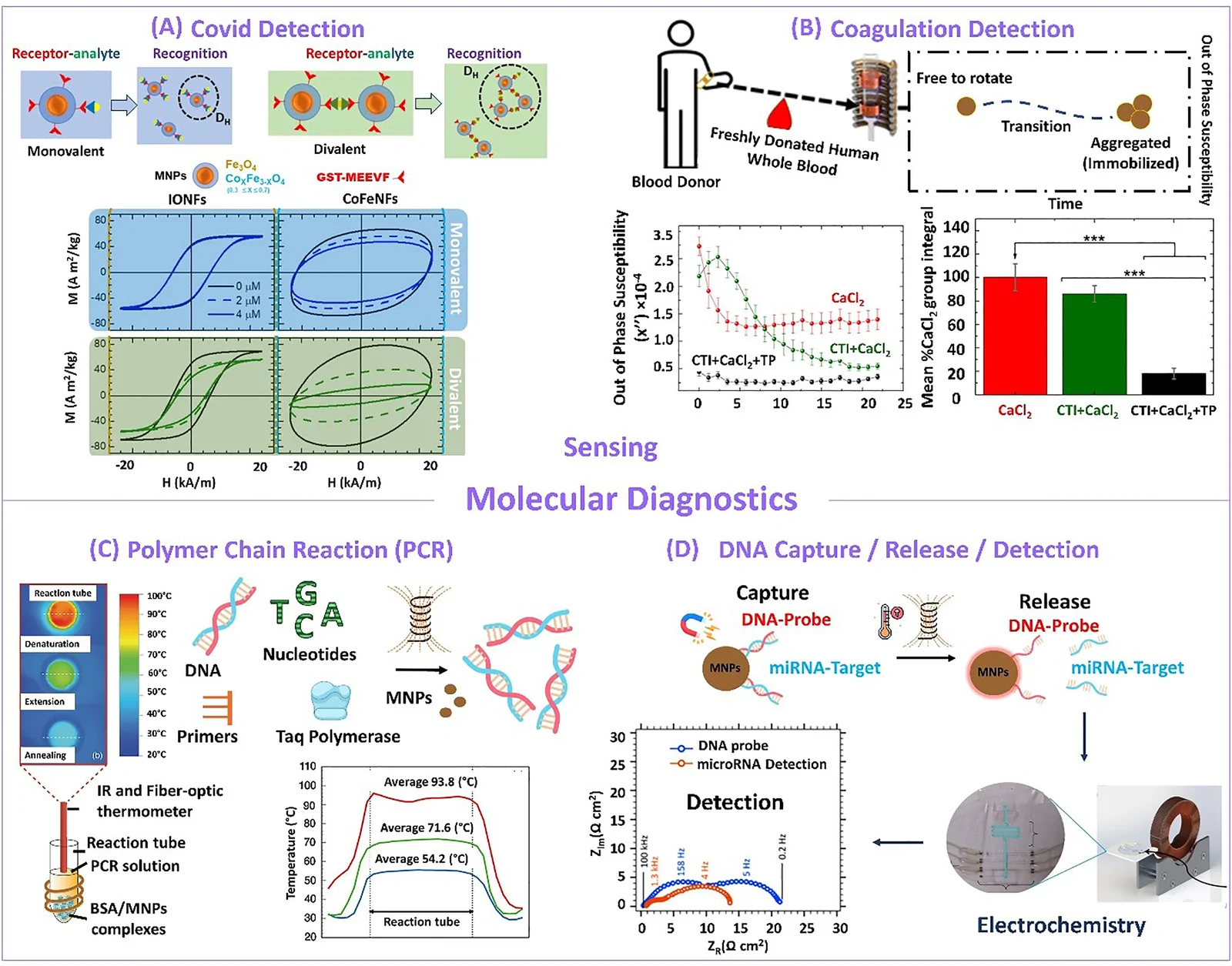
Examples of MI in molecular diagnostics including sensing and biotech applications. (A) Covid detection [adapted/reproduced from ref. 115 with permission from Royal Society of Chemistry,^115^ Copyright 2009].^115^ (B) Coagulation detection^112^ [adapted/reproduced from ref. 112 with permission from Royal Society of Chemistry,^112^ Copyright 2024] (C) PCR^121^ [adapted/reproduced from ref. 121 with permission from Elsevier,^121^ Copyright 2015].^121^ (D) DNA capture by magnetic field gradient and release *via* de-hybridization by AMF activation [adapted/reproduced from ref. 113 under the Creative Commons CC BY 4.0 license,^113^ Copyright 2021].^113^

Before this study, Dong *et al.* monitored the aggregation state of the iron-Au dimers to detect the aflatoxin B1.^117^ They used hydrogen peroxidase reaction to trigger the aggregation of the dimers. However, the binding state of MNPs in this case is monitored through the recording of the transverse relaxation time which in case of aggregation is suppressed. Together, these studies illustrate how target binding is translated into changes in MNP clustering or mobility, which in turn modulate their magnetic hysteresis or relaxation time signatures.

As well as detection of isolated molecular targets, the same AC magnetometer signal principles used by Sanz-de Diego *et al.* can be seen implemented to investigate dynamic processes in complex biological matrices.^115^ Cabrera *et al.* present a magnetic coagulometry platform capable of real-time monitoring of coagulation dynamics in human whole blood.^112^ The study employs sucrose-coated iron oxide MNPs, synthesized *via* co-precipitation, which remain colloidally stable under physiological conditions. Upon incorporation into fresh whole blood samples, the group tracked the out-of-phase component (*χ*″) of the AC magnetic susceptibility signal over time, using it to assess MNP mobility during fibrin clot formation (Fig. 8B). A marked decrease in *χ*″ component was observed as coagulation progressed, attributed to rapid immobilisation and compaction of the MNPs within the forming clot. Notably, when blood was pre-treated with direct oral anticoagulants (DOACs), the *χ*″ signal declined more gradually, revealing clotting delays not detectable by standard clinical assays such as PT and aPTT. The authors suggest that further integration of this technique into point-of-care devices could significantly improve the clinical management of bleeding and thrombotic disorders.

Finally, on-chip systems have been targeted in electrochemistry such as in mRNA detection (Fig. 8D).^113^ A study by MC Horny *et al.* developed a microfluidic lab-on-chip that aimed to combine MIH and electrochemical detection to release and detect microRNA targets avoiding global heating of the sample. DNA duplexes containing a miRNA-122 mimic target were immobilized on DNA-functionalized core–shell nanoparticles composed of γ-Fe_2_O_3_@SiO_2_ PEG/NH_2_ (cores approximately *ca.* 60 nm and core–shell structures approximately 98 nm). AMF was applied (180 kHz, 29 kA m^−1^) while MNPs were flowing through the microchannel on the chip at two different flow rates. At 0.11 µL s^−1^ the time under the AMF was 3 s, whereas at 0.01 µL s^−1^ it was 12 s. The 0.01 µL s^−1^ condition produced DNA release comparable to both off-chip MIH and global heating at 95 °C for 20 min, while the microchannel temperature remained around 28 °C. After release, the targets were captured on DNA-modified amorphous carbon nitride microelectrodes. Optimising the chip geometry enabled a reduction in the total time to 30 minutes, allowing a rapid turnaround of target release and detection on the same device. Studies such as these create a foundation for further MNP-based magnetic monitoring in whole blood and other biological fluids, with the potential to harness tailored MNP architectures relevant to the application type.

#### 2.3.2 PCR

PCR (polymerase chain reaction) is arguably one of the most widely used tools in molecular diagnostics, used for the amplification of specific DNA segments for a variety of different applications. Seeking improvements in specificity and efficiency, incorporation of NPs (termed nanoPCR) has driven recent progress in the field, with positive results using quantum dots, carbon nanotubes and gold NPs.^118^ Relevant to this review, emerging efforts explore MNPs and MIH as a driver for PCR thermal cycles (Fig. 8C). Higashi *et al.*^119^ were among the first to study and experiment with the effect of MNPs on PCR efficiency. The group employed commercially available MNPs (Fe_3_O_4_/Fe_2_O_3_, 10 nm) chemically modified on the surface with anionic and cationic surfactants, before dispersing in different PCR reagents without application of a MF. Thermal cycling, provided by a standard thermocycler, induced surface denaturation and a reduction in zeta potential leading to aggregation in the form of clusters, in whose cavities the Taq polymerase was retained. The sequestration of the enzyme reduces its availability within the reaction solution resulting in a PCR inhibition at increasing MNPs concentrations. Further research by the group demonstrated that a static magnetic field (DC) induced chain-like MNP aggregates that sequestered Taq polymerase, increasing the frequency of a rotating MF (0–20 Hz) modulated cluster structure and progressively reduced PCR efficiency, likely due to NP-driven nano-stirring effects.^120^ These early inhibitory effects drove efforts to better control MNP–enzyme interactions. The same authors proposed a corrective approach using albumin (BSA)–functionalised MNPs to reduce non-specific adsorption of Taq polymerase enzyme within the NP clusters, which, when subjected to an AMF (395 kHz), both improved PCR efficiency and generated sufficient heating (up to 94 °C) to enable thermal cycling. This was interestingly comparable in duration and performance to standard PCR, representing good progress for nanoPCR platforms (Fig. 8C).^121^ Seeking to miniaturise the PCR systems, Kim *et al.* developed a micro-heater system in which MNPs embedded within a PDMS matrix in the sample chamber enabled efficient DNA amplification (>90%) under an AMF, though limitations in nanoparticle distribution and the need for high-frequency excitation (492 kHz) restrict portability.^122^ Building on this concept, Li *et al.* developed a portable qPCR using Fe_3_O_4_@gelatin nanoclusters as both magnetic probes and NIR-activated heat sources, offering rapid yet stable heating up to 90 °C in 15 s with greater thermal stability than traditional plasmonic systems.^123^ In parallel, Chen *et al.* developed a complementary-metal-oxide-semiconductor-based multilayer micro-coil chip integrated on printed circuit boards, where localized Joule heating enabled magnetic bead transport and high-efficiency thermal cycling, achieving 95 °C with just 42.6 mW and heating/cooling rates of ∼19.7 °C s^−1^ and ∼11.8 °C s^−1^, offering promise for single-cell PCR miniaturisation despite limited adoption in current experimental literature.^124^

In contrast, the high-sensitivity immune-PCR system developed by Qiu *et al.*^125^ combines magnetic and plasmonic functionalities using antibody- and primer-functionalised Fe_3_O_4_@Au NPs, where standard heating techniques are complemented by magnetic target isolation and optical signal enhancement to accelerate and simplify interleukin-6 (IL-6) detection in a single-tube format, suggesting a potential application of MIH. Like many of the applications we have discussed, exploration of NP formulations is key to advancing applications. Luckily in nanoPCR, this began early. A 2016 study^126^ made a comparison of gold NPs, silver NPs, and iron oxide MNPs, showing an increase in PCR yield of up to 190% despite their inherently low thermal conductivity. This enhancement is attributed to improved specific adsorption of reagents and bidirectional cluster alignment, which together facilitate more efficient heat transfer. Moreover, the ease of magnetic removal by accumulation to magnetic gradients makes Fe_3_O_4_ MNPs particularly convenient and sustainable, confirming their suitability as promising candidates for nano-PCR applications. These developments suggest a future of smaller, faster and more portable PCR systems powered by MNPs, with potential reductions in power consumption and therefore a lower environmental footprint.

## 3. MI for the synthesis of materials and industrial manufacturing

Beyond biomedical applications, MI has also branched into material synthesis and manufacturing, where the advantages of tight temperature control can be effectively harnessed for reaching a much broader temperature range. In the following section, we will look specifically at applications in materials synthesis (Fig. 9), polymer curing (Fig. 10) and beyond for helping different manufacturing processes (Fig. 11).

### 3.1 Synthesis & phase transformation

Bulk heating is central to many common NP synthesis techniques. However, uncontrolled heterogeneous heating can result in NP products with low quality and poor properties (wide particle size distributions and loss of shape control). MIH in NP synthesis seeks to replace these slow and often energy-intensive approaches with smart, targeted heating generated directly by NPs. As the heat originates at the nanoscale surface of the NPs rather than requiring the bulk solution transfer from external sources into the solution, it means that reactions can proceed within shorter time scales, with far lower overall reactor temperatures. Benefits include improved control over particle nucleation and growth, allowing precise tuning of size, shape, and crystallinity. Moreover, the ability to confine heating to flowing regions containing magnetic seeds enables continuous, in-flow NP synthesis, providing a pathway for scalable, reproducible production.

Recent studies demonstrate these effects across diverse material systems of inorganic NPs. To give an example, carbon-based nanocomposites are typically produced through pyrolysis and hydrothermal methods, which are often time-consuming, taking from several hours to, in some cases, days, and can be challenging to produce in non-equilibrium phases. Lu *et al.*^127^ reported the first instance of MIH-quenching for the rapid production of carbon-FeNi spinel oxide nanocomposites, with a reaction time of mere seconds. In this study, a mixture of NiCl_2_·6H_2_O, FeCl_3_, and urea in water was applied dropwise onto carbon paper, which was then dried at room temperature and placed between two iron sheets (2.5 cm × 2.5 cm × 0.1 cm). This setup was positioned at the center of a four-turn induction coil with a 5 cm diameter (solenoid current of 200 A and *f* = 30 kHz), where MIH was performed at *f* = 30 kHz, reaching temperatures of 200–300 °C in 4 seconds (Fig. 9A). After heating, the sample was quickly quenched by dropping it into an ethanol-dry ice solution (−78 °C) located near the induction coil. The resulting nanocomposite demonstrated exceptional performance in the oxygen evolution reaction (OER), achieving a remarkably low overpotential of just +260 mV to reach a high current density of 100 mA cm^−2^. This demonstrates how rapid heat-quenching enables precise structural design of functional nanocomposites for electrocatalysis. In another work, graphene growth by chemical vapour deposition using RF MIH was reported.^128^

**Fig. 9:**
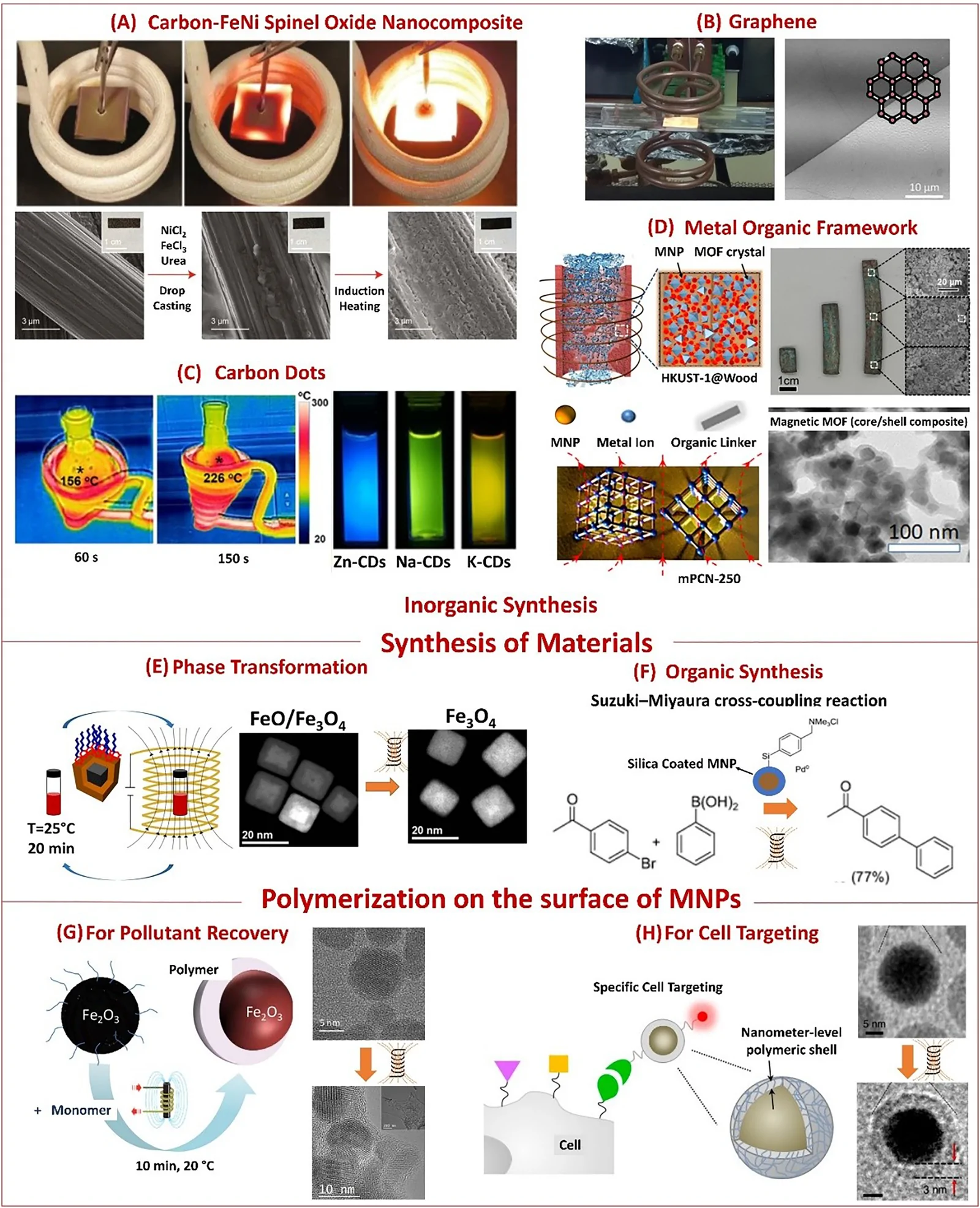
MIH application for the synthesis of materials. Inorganic synthesis: (A) ultrafast preparation of nonequilibrium synthesis of carbon-FeNi spinel oxide nanocomposites [adapted/reproduced from ref. 127 under the Creative Commons CC BY 4.0 license,^127^ Copyright 2022] (B) potential large scale and rapid manufacturing of graphene [adapted/reproduced from ref. 128 with permission from American Chemical Society,^128^ Copyright 2013] (C) large-scale production of multi-fluorescence carbon dots [adapted/reproduced from ref. 129 with permission from John Wiley and Sons,^129^ Copyright 2019] (D) continuous in-flow synthesis of a HKUST-1@wood MOF-based composite (top) [adapted/reproduced from ref. 130 with permission from American Chemical Society,^130^ Copyright 2024] and solvent-free synthesis and regeneration of mPCN-250 magnetic framework composites by MIH (bottom) [adapted/reproduced from ref. 131 with permission from American Chemical Society,^131^ Copyright 2019] (E) phase transformation: by oxidation of core-shell FeO/Fe_3_O_4_ nanocubes to Fe_3_O_4_ phase [adapted/reproduced from ref. 132 under the Creative Commons CC BY 4.0 license,^132^ Copyright 2016]. Organic synthesis examples by MIH: (F) Suzuki-Miyaura and Heck coupling reactions using MNPs functionalized with Pd(0) under flow conditions [adapted/reproduced from ref. 133 with permission from John Wiley and Sons,^133^ Copyright 2008]. Polymerization on the surface of MNPs: (G) thin shell growth of acrylamide by thermopolymerization around the MNPs for pollutant recovery [adapted/reproduced from ref. 134 with permission from American Chemical Society,^134^ Copyright 2018] (H) polymerization of polyvinylpyrrolidone (PVP) on the surface of MNPs, used as platform for further post-surface functionalization for cell targeting applications [adapted/reproduced from ref. 135 with permission from American Chemical Society,^135^ Copyright 2021].

Hot-wall furnaces scale poorly for large-area graphene because they heat an entire reactor volume to grow graphene on a very thin metal foil. By contrast, RF induction deposits energy directly into the conductive Cu foil *via* eddy currents, enabling a cold-wall reactor (reactor walls remain comparatively cool) with fast thermal ramps (Fig. 9B). The authors grow high-quality monolayer graphene on 125 µm oxygen-free high-conductivity (OFHC) copper foil, reaching 1035 °C in ∼2 min and cooling at ∼30 °C s^−1^, while the quartz wall temperature stays <300 °C throughout growth; the resulting graphene shows high quality by Raman and field-effect transistor (FET) mobilities up to ∼14 000 cm^2^ V^−1^ s^−1^.

These next two examples of inorganic NP synthesis work using a similar mechanism, presenting MNPs as the ‘heaters’ rather than eddy current illustrated above. Zhijie Zhu *et al.* took a different approach, instead harnessing the metal substrate itself for the rapid and large-scale synthesis of multi-fluorescent carbon dots (CDs) under AMF (Fig. 9C).^129^ They achieved up to 85 grams in just an hour with a yield of approximately 60%, challenging the traditionally low yield of CD synthesis that has limited their widespread application. The MIH method enhances the reaction by facilitating better energy transfer through its effective heating mechanism. CDs with luminescence in blue, green, and yellow were produced through a hydrothermal method using carbamide and citrate, combined with three distinct cations (Zn^2+^, Na^+^, K^+^), respectively. The reactions were conducted by rapidly heating citrate salt and carbamide precursors to 230 °C within 3 minutes by exposing SPIONs (*ca.* 20 nm, Aladdin-reagent, Shanghai, China) to a robust EMF at 450 kHz. These CDs exhibit strong fluorescence under UV light and are characterized by excellent monodispersity and water solubility. A bar coating technique was employed to create large-area emissive polymer/CDs films, which have the potential to enhance wound healing efficiency.

MIH can also be extended to the synthesis of complex materials such as metal–organic frameworks (MOFs), which are porous crystalline networks formed by coordinating metal ions or clusters with organic linkers and have uses in gas storage, catalysis, sensing, and drug delivery (Fig. 9D). Traditional solvothermal and hydrothermal MOF synthesis methods often require extended reaction times and high temperatures, which can limit scalability and uniformity.^136^ A recent study by Wang *et al.*^130^ demonstrated the continuous in-flow synthesis of MOFs in wood microchannels using MIH for CO_2_ capture. Fe_3_O_4_ (24 nm, hydrothermal synthesis) embedded in the microchannels act as nano-heaters filler under an AMF (24 kA m^−1^), rapidly raising the temperature to 130.5 °C within 5 minutes (Fig. 9D – top). Consistent nucleation and continuous growth of MOF crystals was reported, reducing synthesis time and enhancing product homogeneity. Notably, MIH allows for precise temperature control, which is crucial for tuning MOF properties such as porosity and defect density.

Typical MOF synthesis protocols often require large solvent volumes and long reaction times. To counter this, Tao *et al.*^131^ developed a solvent-free MI framework synthesis approach in which Fe_3_O_4_ MNPs dispersed within powder precursors act as localized nanoheaters under an AMF, directly initiating MOF crystallization through localized induction heating rather than bulk thermal conduction (Fig. 9D – bottom). In this method, commercial or synthetic Fe_3_O_4_ MNPs are physically mixed with MOF precursors and exposed to an AC MF, where increasing MF strength, MNP loading, and reaction time enhances framework yield. Beyond rapid solvent-free synthesis, the resulting magnetic MOFs exhibited excellent results for gas capture and separation applications.

Beyond synthesis, MIH also enables controlled structural transformations, driving crystalline restructuring or shell formation. This overcomes inefficiency and control issues we have previously discussed associated with bulk heating. For instance, Koslovskiy *et al.* demonstrated that IONPs doped with rare-earth elements such as gadolinium undergo phase transformation during conventional thermal annealing.^137^ This study established phase transformations of Fe_2_O_3_ → Fe_2_O_3_/GdFeO_3_ → GdFeO_3_ based on annealing temperature. The predominance of the GdFeO_3_ phase increased nanoparticle size from 15 to 40 nm. These changes in phase composition and size were shown to increase NP heating rates. In contrast, Lak *et al.* used MIH to induce the oxidation of Fe_1−*x*_O/Fe_3−*δ*_O_4_ core-shell nanocubes into the Fe_3−*δ*_O_4_ phase, with the goal of enhancing their magnetic properties for potential biomedical and catalytic applications (Fig. 9E). The PEGylated core-shell nanocubes (17 ± 2 nm, thermal decomposition synthesis) were exposed to 25 cycles of an AMF (330 kHz and 14 kA m^−1^), which triggered phase transformation from poor magnetic wüstite (FeO) phase of the core to highly magnetic Fe_3−*δ*_O_4_ phase of the shell. Large, coherent Fe_3−*δ*_O_4_ domains were formed, resulting in high saturation magnetization and superparamagnetic behaviour at room temperature. The SAR of the final monophase MNPs, after core phase conversion doubled, showing improved heating performance after transformation. Unlike traditional thermal annealing methods, MIH-based annealing facilitates oxidation at a macroscopic temperature below 37 °C.^132^

Such magnetothermal energy of MNPs could also be used in organic synthesis. Ceylan *et al.*^133^ pioneered the use of SPIONs as heat-mediating agents in various organic chemical reactions within continuous in-flow microreactor setups (Fig. 9F). They demonstrated the feasibility of conducting Suzuki–Miyaura and Heck coupling reactions under continuous flow conditions by utilizing the localized heat generated by SiO_2_-coated Fe_3_O_4_/Fe_2_O_3_ NPs (diameter < 100 nm), which were functionalized with catalytically active palladium. When exposed to an AMF (25 kHz), the catalytic MNPs-based platform, the reaction temperature swiftly rose to 200 °C within minutes, whereas a traditional batch reactor requires approximately 70 minutes. MIH not only enhances reaction efficiency but also offers adjustable heating profiles by modifying AMF parameters, making it a promising tool for advanced organic synthesis.^133^ In addition to inorganic and organic chemical reactions, Lim, *et al.*^135^ reported an unconventional application of MIH to form nanoscale polymerization on MNPs (Fig. 9G). The authors developed a magnetothermally activated polymerization (MAP) strategy using 15 nm CoFe_2_O_4_@MnFe_2_O core-shell NPs exposed to an AMF of 32–40 kA m^−1^ at 500 kHz, generating nanosecond localized heat pulses confined within 1–3 nm from the nanoparticle surface without significant bulk heating. The transient surface temperature reached 187 °C within 0.3 ns, while cooling occurred within 10 ns, enabling radical polymerization of monomers such as *N*-vinylpyrrolidone (PVP). The shell thickness could be precisely controlled from 1 to 3 nm by adjusting the AMF intensity. The MAP method enabled modular grafting of various functional polymers containing amine, carboxyl, epoxide, benzyl chloride, and thiol groups. The original magnetic saturation (120 emu g^−1^) was preserved, and the paper reports a compact hydrodynamic size (23 nm). Additionally, copolymerization allowed fine tuning of NP surface charge from +20 to −25 mV, where zwitterionic near-neutral NPs showed 3.8-fold higher folate-receptor targeting specificity toward HeLa cells compared with charged NPs. This study demonstrates that MNPs with desired surface charge can be produces for cell-specific targeting applications.

Similarly, Griffete *et al.*^134^ exposed Fe_2_O_3_ (10 nm) to an AMF to generate localized heat sufficient to initiate thermoactivated radical polymerization directly at the nanoparticle surface without increasing the bulk solution temperature (Fig. 9H).^134^ First, they a polyacrylamide shell was grown around MNPs and then a second functional shell, a molecularly imprinted polymer (MIP), was then thermo-polymerized around the polyacrylamide-coated MNPs. This enabled multilayer surface grafting for pollutant removal applications.

### 3.2 Thermal curing

As mentioned in the previous section, polymerization can be initiated by heat. MIH can be exploited not only for surface polymerization but also for producing high-performance polymers, coatings, composites, and adhesives where precise curing control is required. This localized heating by MNPs embedded within polymeric matrices avoids temperature gradients and overheating which can degrade sensitive monomers.

This concept is illustrated nicely by a study exploring MIH-initiated acrylamide polymerization. Vassallo *et al.*^138^ used spherical CoFe_2_O_4_ NPs (13.7 ± 1.2 nm, coprecipitation synthesis) embedded within the monomer solution and exposed to an AMF (100 kHz and 40 kA m^−1^ for 10 minutes) reached a SAR value of 60 W g^−1^ and heated to 60 °C within 5.8 minutes (Fig. 10A). This heat triggers initiator to decompose, forming free radicals which, in turn, start the polymer chain growth with faster kinetics and improved control over molecular weight distribution. Polymerization also occurred when MNPs were concentrated at a single reactor corner under AMF exposure. Controlled MIH curing has also been demonstrated in Fe–poly(dimethylsiloxane) (PDMS) composites. A study by Darvish *et al.*^141^ in 2015 reported preparation of Fe–PDMS materials using MIH rather than oven heating, enabling faster formation without a catalyst and improved thermal stability. Homogeneous mixtures were obtained by adding 2 and 3 wt% Fe_3_O_4_ (5–9 µm) to PDMS, producing 20% and 30% Fe–PDMS composites. Curing during AMF exposure (142 kHz, 1 kW) yielded SAR values of 36.78 W g_Fe_^−1^ for 20 wt% Fe and 31.62 W g_Fe_^−1^ for 30 wt% Fe.

**Fig. 10:**
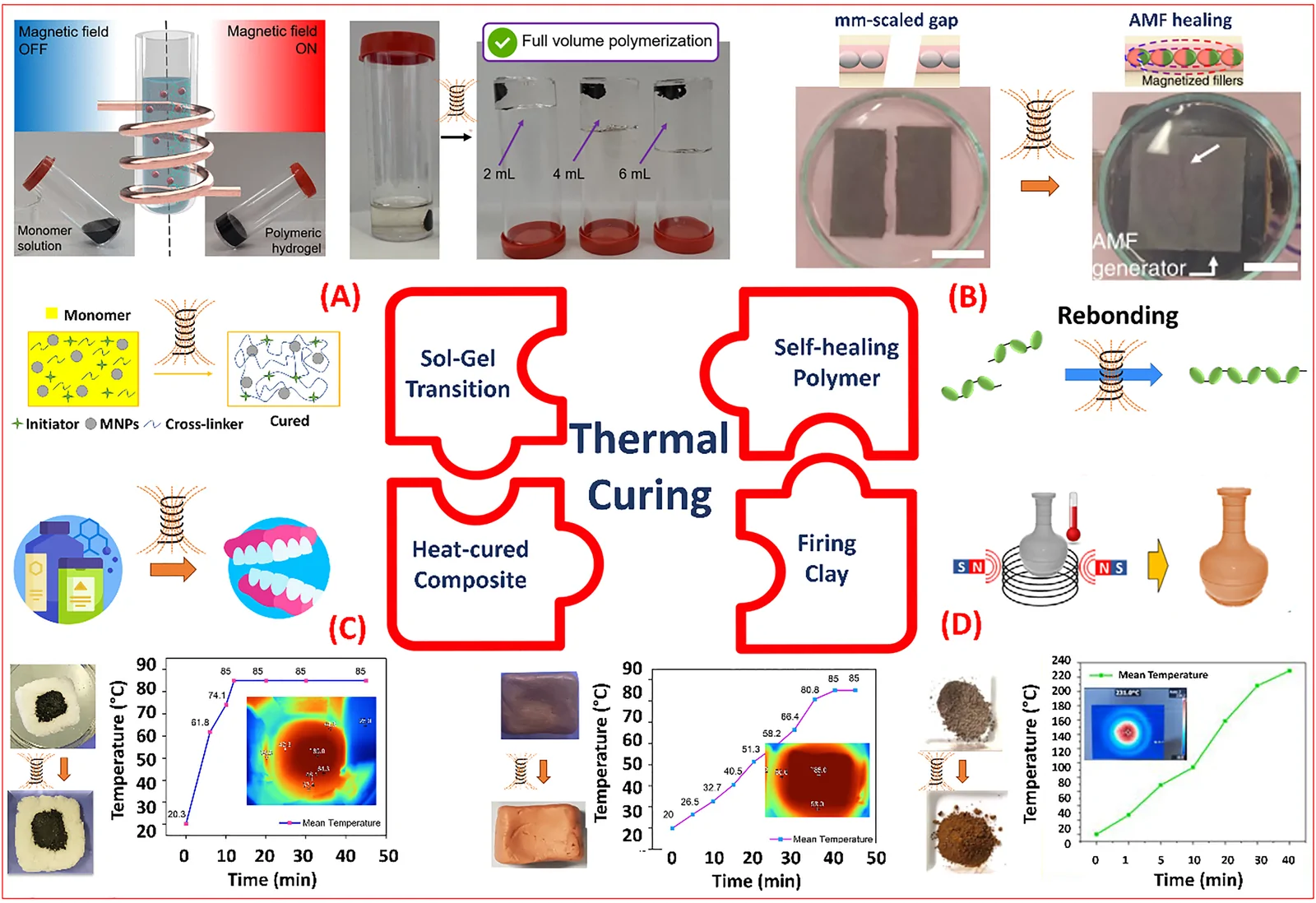
MIH application for thermal curing process. (A) Liquid solution transition to solid gel for MNPs-doped polyacrylamide hydrogel [adapted/reproduced from ref. 138 with permission from American Chemical Society,^138^ Copyright 2024]. (B) Magnetic microparticles-doped PDMS for self-healing of percolation networks in magnetoresistive sensor applications [adapted from ref. 139 under the Creative Commons CC BY 4.0 license,^139^ Copyright 2022]. (C) Curing denture-based materials [adapted/reproduced from ref. 140 under the Creative Commons CC BY 4.0 license,^140^ Copyright 2026] (D) sintering the clay for art pottery application in wet paste (left) and dry powder (right) states [adapted/reproduced from ref. 140 under the Creative Commons CC BY 4.0 license,^140^ Copyright 2026].

These thermally responsive systems further enable self-healing polymers. Self-healing polymer systems typically require heating to increase chain mobility above the glass transition temperature *T*_g_ at damaged interfaces, but conventional heating is intrinsically non-selective. It heats the entire component, risks thermal degradation, and is impractical for buried or inaccessible cracks in composites. MIH addresses this limitation by embedding SPIONs as remote nanoheaters within the polymer network; upon exposure to an AMF, heat is generated locally at the particle-polymer interfacial regions, enabling controlled interdiffusion across crack faces without globally heating the structure. This concept is highlighted in the self-healing polymers literature as a compelling pathway for on-demand, spatially confined repair, especially in situations where direct thermal or photonic stimulation is not feasible.^142^

Several studies have demonstrated magnetically triggered self-repair in diverse polymers. Xu *et al.*,^139^ reported self-healable, printable magnetoresistive sensors in which an AMF actively drives magnetic fillers to reform percolation networks, enabling rapid electrical self-healing (Fig. 10B).^139^ The functional fillers are commercial soft-magnetic Ni_81_Fe_19_ microparticles embedded in a double-network binder composed of crosslinked PDMS and supramolecular polyborosiloxane. During fabrication and repair, the authors used an AMF (50 Hz, <103 kA m^−1^) to generate two coupled effects including magnetic attraction that pulls separated microparticles into contact across cracks, and field oscillation that mechanically agitates the particles helping to “mill off” thin insulating binder layers and thereby restore electrical pathways. As a result, damaged conductive traces recover 100% electrical performance in seconds, and remained repeatably healable over multiple cycles. The nonselective bulk heating can deform insulation and accelerate thermal degradation, while insufficient heat at the damage site limits interfacial reptation and welding.

In another work, Adzima *et al.*^143^ demonstrated an externally triggered self-healing thermoset based on a thermoreversible Diels–Alder (DA) covalent adaptable network (PPTF/DPBM) loaded with magnetically susceptible particles that enable *in situ* repair *via* self-limited hysteresis heating, avoiding the drawbacks of conventional oven heating (nonselective heating, slow thermal diffusion, and risk of irreversible side reactions). The heating agent is commercial ferromagnetic chromium(iv) oxide (CrO_2_; Magtrieve, Sigma-Aldrich) dispersed into the DA network at 0.1–10 wt%, where AMF exposure drives a reversible gel/sol transition (retro-DA) to allow viscous flow and crack closure, followed by regelation upon cooling. Magnetic activation was achieved using a 7.5 kW RF generator with a 3-turn solenoid coil, applying an AMF at 390 kHz; reshaping was demonstrated under 12 kA m^−1^ for 150 s, while fracture healing was performed at 11 kA m^−1^ for 300 s. They showed that the material shows liquidlike behaviour (viscosity 3 Pa s) at 110 °C, which is the practical “sol state” used for reshaping and crack closure under AMF. When the AMF is turned off, the sample is allowed to cool and as temperature drops below the gel-point (92 °C) the equilibrium shifts back toward Diels–Alder bond formation, restoring the crosslinked network (gel state). The key mechanistic advantage is that CrO_2_ has a Curie temperature near 113 °C, providing a self-limiting heating ceiling that mitigates thermal overshoot and suppresses irreversible degradation; correspondingly, the material recovered its flexural modulus and ultimate strength over ten fracture-heal cycles with negligible performance loss, highlighting MIH as a route to rapid, repeatable repair in thick or opaque composites where optical heating is ineffective.

Corten, *et al.*^144^ reported a repeatable, non-contact self-repair strategy for thermoplastic methacrylate films enabled by MIH, addressing the key limitation of conventional thermal healing (bulk oven heating causes slow, heterogenous heat transfer and can distort thin films before sufficient chain mobility is achieved at the crack interface). They fabricated p-MMA/nBA/HDFMA films containing uniformly dispersed SPIONs (12 nm) and demonstrated that physically separated cut surfaces can be rejoined and mechanically restored under an oscillating MF without adding any chemical healing agent. The repaired films retained stress and strain essentially unchanged over multiple (up to six) damage-repair cycles, indicating minimal nanoparticle aggregation and preserved heating performance.

Similarly, Yang Y, *et al.*^145^ showed a self-healing dielectric strategy in which SPIONs migrate to electrically damaged “tree” channels in polypropylene and then act as localized nanoheaters under an AMF, enabling restoration of insulation performance that cannot be recovered by conventional additives. Healing was triggered by a uniform AMF at 968 kHz, so that the sample reached 117 ± 1 °C after 15 min (bulk), enabling localized fusion and sealing of hollow channels. The study further indicated that resistivity and dielectric strength can be restored over repeated ageing-healing cycles without requiring high magnetic filler fractions.

Ahmed and Ramanujan developed self-healing MNPs-polymer composites for wire-insulation repair.^146^ Thermoplastic polyethylene vinyl acetate (EVA) films containing 10–16 wt% MNPs were fabricated by solution casting, and self-healing was triggered through MIH at (475 kHz, 4 kA m^−1^), yielding temperatures in the range of 150–220 °C which promotes interfacial chain diffusion. Under these conditions, MNP-filled films achieved complete healing (up to 100%) within 20 minutes, while maintaining electrical insulation behavior and showing no measurable thermal degradation. This outcome demonstrated how induction heating can localize, regulate, and repeatably deliver welding temperatures more safely than conventional external heating for polymer insulation repair.

A first attempt to prove the feasibility of curing dental prostheses (polymer-based composites) by MIH was reported by the Pellegrino group (Fig. 10C).^140^ Iron oxide nanocubes (IONCs, 16 nm, 4.2 mg) synthesized by solvothermal method were selected for their magnetothermal conversion efficiency and were embedded in the acrylic denture-based materials, a mixture of MMA/PMMA (liquid/powder), which exhibited rapid heating under AMF (15 kA m^−1^, 264 kHz), reaching 85 °C within 40 minutes. This temperature rise was sufficient to trigger the polymerization of MMA in the presence of the initiator benzoyl peroxide as observed by macroscopic consolidation and changes in color of the composites. Nevertheless, the polymerization kinetics require further investigation. Moreover, the same group suggests that the original brownish/orange colour of the MNPs (of Fe_3_O_4_ composition) may be further exploited to reproduce the natural colour of the teeth for the acrylic dentures or as mentioned in the previous section as sterilizing tool to eliminate biofilms.^76,78^ As further basic proof of principle, they implemented the same sintering concept on clay materials. They reported different heating profiles for IONCs-incorporated clay composite into dry (Fig. 10D – left) and wet phase (Fig. 10D – right) when exposed to an AMF. The choice of art clay was based on the evident colour change from grey to red after firing. In dry clay, incorporating only 2.5% in weight of IONCs (21 nm) produced a temperature increase to 231 °C within 40 minutes (30 kA m^−1^ and 910 kHz). For wet clay, at even lower MNP weight percentage (0.37) the temperature of the composite rose to 85 °C when exposed to 15 kA m^−1^ at 264 kHz. This application opens to a new spread of semi-finished pottery products to fire clay materials without using traditional kilns.

### 3.3 Fluid viscosity control

As the temperature rises, the viscosity of liquids decreases. In industrial settings (oil, gas, pharmaceuticals *etc.*) high viscosity can add to costs, increasing pressure and reducing flow rates.^151^ It is advantageous therefore, in operations of this scale, to consider opportunities to reduce this, thereby reducing costs. In 2021, Brollo *et al.* reported an innovative experimental flowloop setup to investigate the behaviour of commercially available oleic acid-capped iron oxide NPs (8 nm) and subjected to an AMF of 16 kA m^−1^ and 431 kHz (Fig. 11A). Notably, the flowloop designed for this study was considerably larger than those typically used MIH experiments, accommodating over 50 g of MNPs. They found that, among the solvents tested for MNP dispersion, diesel and toluene exhibited the highest SAR values of 55 W g_Fe_^−1^ and highest Δ*T* equal to 8 °C. Importantly, they observed no temperature increase due to eddy currents under any conditions. This compelling proof of concept demonstrated the potential to alter a liquid's properties through MIH, enabling on-demand viscosity reduction.^147^

**Fig. 11:**
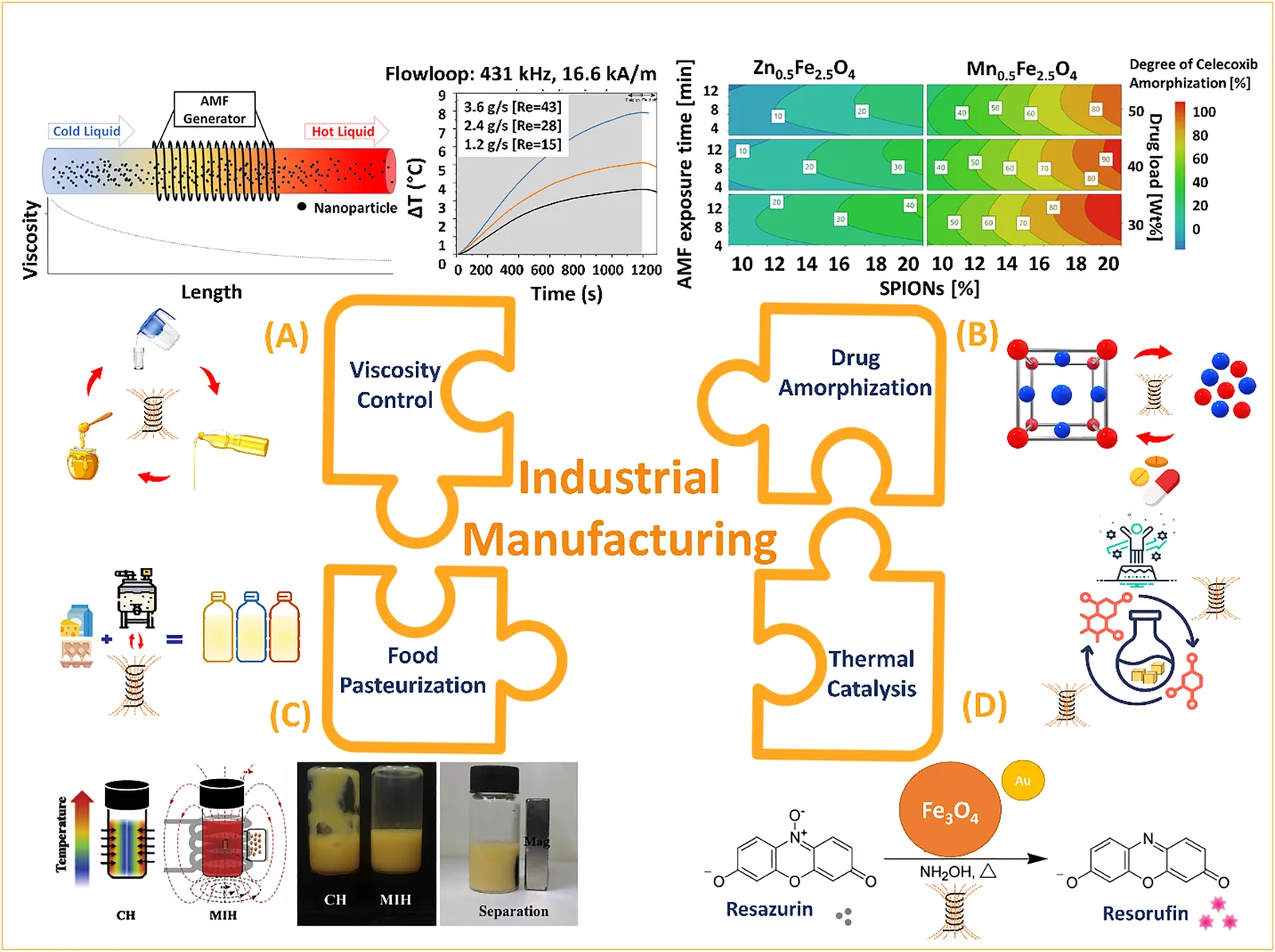
MIH application for industrial manufacturing. (A) Addition of magnetic particles to viscous liquid (toluene and diesel) in the reactor to tune the viscosity through AMF application [adapted/reproduced from ref. 147 with permission from American Chemical Society,^147^ Copyright 2021]. (B) Drug amorphization using MNPs-doped celecoxib [adapted/reproduced from ref. 148 under the Creative Commons CC BY 4.0 license,^148^ Copyright 2022]. (C) Magnetic microbeads used for pasteurization of liquid whole eggs [adapted/reproduced from ref. 149 with permission from Elsevier,^149^ Copyright 2020]. (D) Conversion of resazurin into the fluorescent resorufin through localized catalysis driven by the induction heating of MNPs [adapted/reproduced from ref. 150 with permission from Royal Society of Chemistry,^150^ Copyright 2020].

In 2023, the same team published a further work on MIH-controlled viscosity reduction in flowing heavy crude oil, overcoming the aforementioned limitations for conventional strategies to achieve uniform viscosity in long pipelines such as external convective heating or chemical additives.^152^ The SPIONs were exposed to an AMF generated by a NanoScale Biomagnetics G3 system, with frequencies up to 533 kHz and field intensities up to 14 kA m^−1^, the flowing heavy-oil loop exhibited a maximum temperature difference of 16.3 °C after 50 min at 1 wt% nanoparticle loading and 1.2 g s^−1^ flow rate, while control samples without MNPs showed negligible heating, confirming that heat arises from Néel/Brownian magnetic relaxation rather than eddy currents. This magnetically induced internal heating translates directly into a nearly 45% reduction in crude-oil viscosity, based on independently measured viscosity-temperature correlations, thereby enabling remote, volumetric, and spatially uniform thermal tuning of fluid rheology without bulk external heaters or addition of chemical flow improvers. The study thus established MIH as a non-contact alternative for real-time viscosity control and pipeline flow assurance in viscous industrial fluids.

In addition, Pinheiro *et al*.^153^ systematically investigated MIH for viscosity control in petroleum-based fluids, targeting a long-standing challenge in the oil and gas industry where high-viscosity crude oils require costly external heating, chemical additives, or mechanical interventions to maintain flow assurance. The commercial SPIONs (Fe_3_O_4_) supplied by NanoGap, were delivered as oleic-acid-coated particles with a mean core diameter of 9 nm m in diesel. Colloids containing 0.5–1.0 wt% NPs were dispersed in diesel, heavy crude oil (*η* ≈ 200 mPa s), and waxy crude oil (*η* ≈ 1000 mPa s) to form stable nanofluids. MIH experiments were performed using an AMF at 770 kHz with field amplitudes up to 28 kA m^−1^. Under these conditions, 1.0 wt% NPs in diesel reached temperature rises up to 95 °C, while 0.5 wt% NPs in heavy and waxy crude oils achieved Δ*T* = 57 °C and 43 °C, respectively, demonstrating effective internal heating even in highly viscous media. The resulting temperature increase translates directly into substantial viscosity reduction, confirming that Néel-relaxation-driven nanoscale heat dissipation can volumetrically warm viscous hydrocarbons, enabling remote, contactless, and spatially uniform thermal tuning of rheology without external heaters or chemical flow improvers.

Another study by Almeida *et al.* revealed that the viscosity of clarified butter oil and paraffin was reduced through localized heat generated by Zn_*x*_Fe_3−*x*_O_4_ MNPs (average size of 11–25 nm, produced by thermal decomposition) under AMF exposure (16 kA m^−1^, 570 kHz), confirming the feasibility of thermodynamic control over fluid properties without bulk heating.^154^

Similarly, Aristizábal-Fontal *et al.* found that applying MIH to fluids laden with MNPs leads to significant, reversible reductions in the viscosity of extra-heavy crude oil, with the effect being adjustable by modifying AMF parameters such as frequency and field strength. In this study, iron oxide NPs with an average size of 40 nm, synthesized *via* the coprecipitation method, were mixed with extra-heavy crude oil in various dosages ranging from 0 to 50 000 mg L^−1^ and exposed to an AMF to generate localized heat, thereby reducing viscosity. The findings indicated that the viscosity and shear stress of extra-heavy crude oil could be decreased by up to 81% and 78%, respectively, when ferrofluid was present. Overall, the results highlighted the significant potential of this approach in reducing the viscosity of heavy crude oil in pipelines or reservoirs, facilitating transport, and could lead to lower demands of energy needs.^155^

Conventional oil production methods typically recover only 15–35% of the original oil in place, leaving the majority of hydrocarbons trapped in porous reservoir rocks due to capillary forces, unfavourable wettability, and high oil/water interfacial tension. Improving extraction efficiency from mature reservoirs has become an essential technological and economic objective as global energy demand continues to rise. Enhanced oil recovery (EOR) techniques including chemical flooding with surfactants, polymers, and NPs aim to mobilize residual oil by reducing interfacial tension, altering rock wettability, and improving sweep efficiency. However, these methods face persistent challenges such as surfactant adsorption losses on reservoir minerals, high-temperature and high-salinity instability, heterogeneous pore structures, and the extreme viscosity of heavy crude oils, all of which limit displacement efficiency. Consequently, there is strong motivation to develop alternative physical stimulation strategies capable of *in situ* viscosity reduction to improve fluid mobility, particularly in reservoirs where conventional thermal or chemical EOR approaches become inefficient or uneconomical.^156^

Song Y *et al.*^157^ showed viscous crude oil recovery (33.05 g h^−1^ cm^−2^) with high separation efficiency, establishing MIH heating as a safer, non-contact alternative to Joule heating for practical heavy-oil recovery. They engineered a ferrimagnetic sponge that remotely lowers oil viscosity and enables continuous oil recovery under an AMF. In this work, OA-capped iron oxide nanocubes (size of 25 nm) were incorporated into a commercial melamine sponge by a dip-coating process. Under AMF exposure at *f* = 274 kHz and *H* = 30 kA m^−1^, the sponge heated extremely rapidly such that surface average temperature rose to 120 °C within 10 s. Importantly, internal temperatures could exceed the surface temperature due to confined heat transport in pores (145 °C at 10 mm depth when surface temperature was 105 °C), eliminating the deep temperature penetration limitation. The outcome showed that MIH-based heating sharply decreased crude-oil viscosity and accelerated uptake; specifically, when integrated with a self-priming pump.

### 3.4 Drug amorphisation

One of the major challenges facing the pharmaceutical industry is the poor water solubility of many drugs. Polymer-based amorphous solid dispersions are considered a major advancement in overcoming challenges related to poor water solubility and oral absorption. This technique facilitates amorphization by dispersing drug molecules within a polymeric matrix inside a tablet, thereby avoiding the physical instability often encountered during the production and storage of traditional amorphous solid dispersions. However, this method currently faces limitations such as low drug loading, excipient toxicity, and drug degradation.^158^

A study by Ansari *et al.*^148^ introduced a novel application of MIH to induce *in situ* amorphization of celecoxib, a poorly water-soluble drug, for oral administration (Fig. 11B). They explored the feasibility of using SPIONs embedded within tablets to induce amorphization upon exposure to an AMF. SPIONs doped with zinc (Zn_0.5_Fe_2.5_O_4_) and manganese (Mn_0.5_Fe_2.5_O_4_) with sizes ranging from 12–20 nm were synthesized *via* flame spray pyrolysis. These MNPs were then compacted with polyvinylpyrrolidone (PVP) and celecoxib to form tablets. The tablets were exposed to an AMF (588.5 kHz and 11 kA m^−1^) for varying durations from 3 to 15 minutes. A design of experiments approach was employed to assess the effects of MNP composition (Zn_0.5_Fe_2.5_O_4_ and Mn_0.5_Fe_2.5_O_4_), doped SPION content (10–20 wt%), drug load content (30–50 wt%), and exposure time of AMF (3–15 min) on the degree of drug amorphization. The amorphized celecoxib showed a fivefold increase in solubility in biorelevant intestinal fluid. Importantly, this approach allows amorphization to be activated on demand immediately before administration, offering a route to improved therapeutic performance with reduced processing stress.

### 3.5 Food pasteurization

In a market with high demand for convenience foods, preservation techniques are central to maintaining both the safety and integrity of products. Finding the balance between maintaining nutritional value and taste while inactivating potentially harmful microorganisms can be a challenge and, with this in mind, it is beneficial to both manufacturer and consumer that methods used are as efficient as possible. Conventional pasteurization relies on convective or conductive heat transfer, where food is heated externally in heat exchangers, water baths, or steam chambers typically operating between 60 and 90 °C. In viscous foods, slow heat penetration and temperature gradients can produce cold spots, requiring overheating to ensure microbial safety and often reducing final product quality. MIH is used in other applications for efficiency and uniformity, and there have been exploratory studies in harnessing these advantages for food pasteurisation, with MNPs dispersed at safe, trace levels.

Interestingly, this technique has been applied to pasteurization of viscous foods such as liquid whole eggs (Fig. 11C).^149^ Magnetic microbeads (50 µm) exposed to an AMF (400 kHz, 600 s) generated heat through hysteresis losses, reaching a specific absorption rate of 3108.4 ± 30.6 W g_Fe_^−1^. Uniform bead dispersion enabled even heating throughout the viscous matrix. A 7.6-log reduction of *S. Enteritidis* was achieved at 68 °C within 60 s, outperforming conventional convective heating, which required 210 s at 60 °C for a 6-log reduction. The narrow temperature–time window also prevented protein coagulation, preserving the fresh functional properties of the eggs. These results highlight MIH as a rapid and quality-preserving alternative to traditional pasteurization.

In 2022, a study published in *Foods* introduced an innovative electrode-free, continuous-flow induction heating system specifically designed for sterilization and bacterial inactivation of apple juice.^159^ Wu and co-workers developed a non-conventional induction heat treatment *via* an induced electric field rather than heating reactor walls. The system used a design that combines multiple secondary coils connected in parallel with a variable diameter connection to amplify induced current in a narrow link (heating) tube, enabling short treatment times of ∼20 s in flow without electrodes. A frequency sterilization condition of 40 kHz at 1800 V achieved complete microbial inactivation with a *T*_max_ of 62.5 °C while maintaining physicochemical quality metrics. This system combines a magnetic secondary coil plate with a vibration dielectric coupling structure, achieving rapid and uniform heating in just 20 seconds without introducing magnetic particles. By adjusting design and electrical parameters such as voltage, frequency, and coil geometry, the researchers demonstrated precise control over the process, ensuring the production of safe juice without compromising quality.

Instead, Bañobre-López *et al.*,^92^ used PAA-coated SPIONs (∼10 nm) to inactivate the food-spoilage bacterium *P. fluorescens via* MIH. Heat was generated under an AMF of 8 kA m^−1^ at 873 kHz, with exposure times adjusted to reach different *T*_max_ = 35–55 °C. The optimized formulation (55 °C reached in ∼8 min) resulted in complete eradication, whereas direct heating did not achieve complete kill even at substantially higher bulk temperatures (up to 65 °C). The outcome suggests that MIH can deliver enhanced lethality at lower temperatures, consistent with localized nanoscale heat dissipation near bacteria, supporting a pathway to microbial control while better preserving nutritional quality than conventional thermal treatments.

### 3.6 Thermal catalysis

Magnetically induced catalysis (MIC) is emerging as a route to decouple local temperature from bulk temperature, enabling fast thermal transients, improved energy efficiency, and potentially to mitigate classical heat-transfer bottlenecks such as thermal inertia, nonuniform heating, and heat losses. For instance, Yassine *et al.*^150^ used MIC to convert resazurin, a blue, non-fluorescent dye, into resorufin, a highly red-fluorescent molecule when reduced. This conversion, often used in cell viability assays (*e.g.*, Alamar Blue), occurs *via* dehydrogenase enzymes in the cytoplasm or mitochondria. In this work, citrate-capped iron oxide MNPs (33 ± 8 nm) act as nanoheaters and gold NPs (24 ± 6 nm) provide catalytic activity. These types of NPs were electrostatically co-assembled using the cationic polyelectrolyte PDDA. MI was applied (at 110 kHz) under controlled macroscopic temperature conditions (*e.g.*, bulk temperature regulated to 45 °C) to allow direct comparison with conventional water-bath heating. Under AMF (110 kHz) exposure at 45 °C only, Fe_3_O_4_ nanoheaters efficiently drive Au-catalyzed resazurin reduction efficiently. Notably, catalysis occurs both in solution and within hydrogel matrices, in the latter case demonstrating spatial confinement of reaction zones, a capability that is difficult to achieve with external convective heating (Fig. 11D).

MIH can push catalytic processes closer to intrinsic kinetic limits, known as “cold catalysis” concept referring to non-uniform thermal distributions, where the bulk (macroscopic) temperature remains relatively low, while localized nanoscale regions (*e.g.*, at catalyst surfaces or MNPs) experience significantly high temperatures.^160^ The high surface area, functionalization flexibility, easy magnetic recovery can provide the materials foundation upon which MI heating-based catalysis strategies can be built and compared against conventional thermal catalysis. The main categories of catalytic reactions already under study cover Fischer–Tropsch syntheses, CO_2_ methanation, steam methane reforming, CO oxidation, tandem reaction (ethene dimerization and olefin metathesis), hydrogenation and hydrodeoxygenation described in the following (Fig. 12).^161–166^

**Fig. 12:**
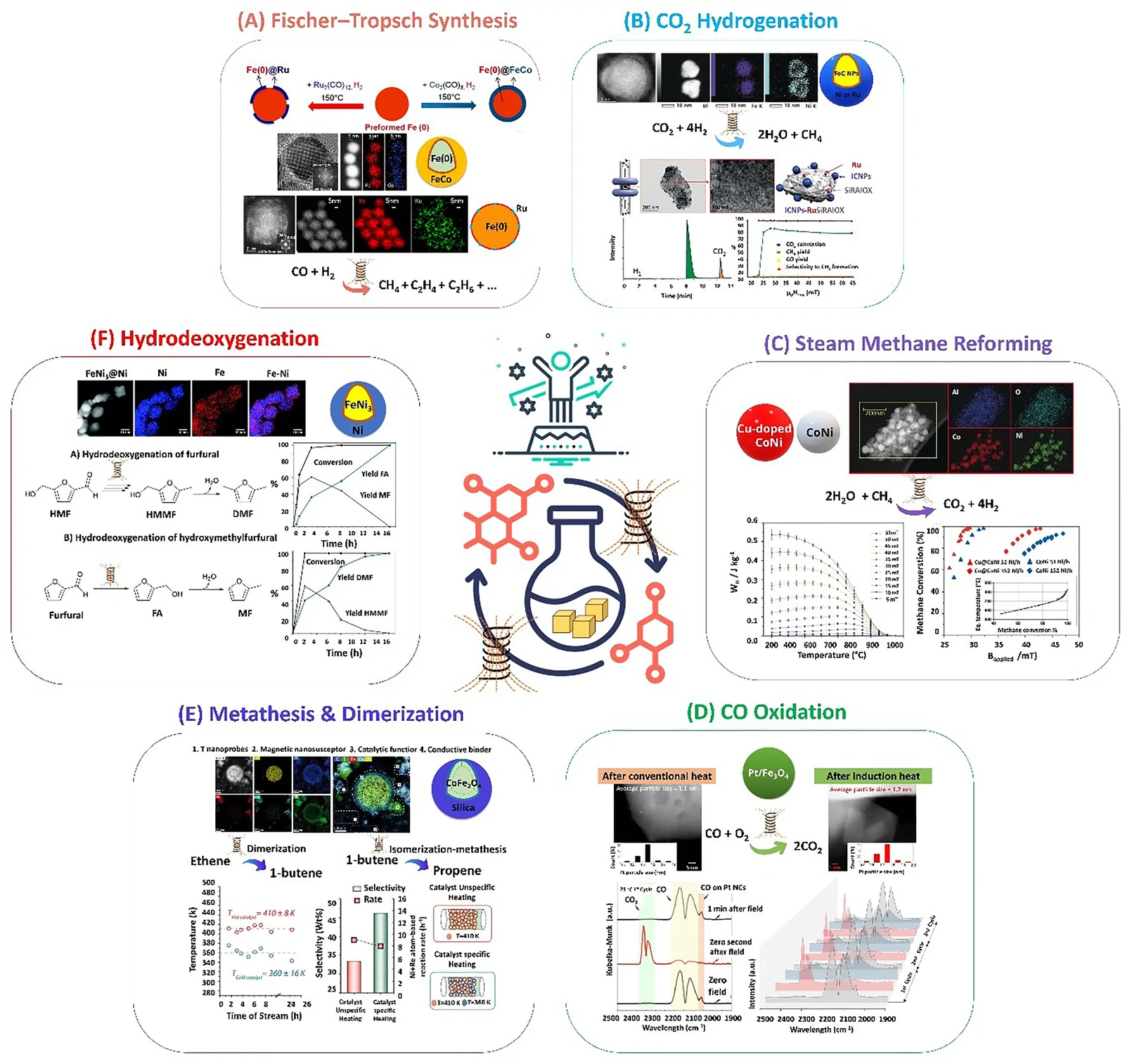
Selected examples of MIH application for catalytic reactions. Six main types of reactions using MIH have been identified: (A) Fischer–Tropsch synthesis: generated heat by monodisperse core-shell Fe(0)@FeCo NPs and a thin discontinuous Ru(0) layer supported on the initial Fe(0) NPs under an AMF used for Fischer–Tropsch Syntheses [adapted/reproduced from ref. 160 with permission from American Chemical Society,^160^ Copyright 2015]. (B) CO_2_ hydrogenation: Iron Carbide NPs and Ru NPs used to generate heat under an AMF in the continuous flow hydrogenation of CO_2_ leading to methane formation [adapted/reproduced from ref. 167 with permission from John Wiley and Sons,^167^ Copyright 2016]. (C) Steam methane reforming using CoNi NPs and Cu-doped CoNi NPs used for methane conversion to hydrogen gas [adapted/reproduced from ref. 168 with permission from John Wiley and Sons,^168^ Copyright 2018]. (D) CO oxidation using Pt/Fe_3_O_4_ MNPs catalysts to generating heat under AMF for CO oxidation [adapted/reproduced from ref. 169 with permission from American Chemical Society,^169^ Copyright 2024]. (E) Metathesis and dimerization used silica-coated CoFe_2_O_4_ as heat-mediated agent under an AMF to perform ethene dimerization and olefin metathesis [adapted/reproduced from ref. 170 under the Creative Commons CC BY 3.0 license,^170^ Copyright 2023]. (F) Hydrodeoxygenation using FeNi_3_@Ni MNPs for hydrodeoxygenation of hydroxymethylfurfural (HMF) and cleavage of lignocellulose-derived products in solution using MI [adapted/reproduced from ref. 171 with permission from Royal Society of Chemistry,^171^ Copyright 2021].

Fischer Tropsch synthesis is a catalytic process developed in the 1920s that converts syngas (carbon monoxide and hydrogen) into high-quality liquid hydrocarbons, such as synthetic diesel, naphtha, and waxes. Meffre and colleagues^160^ synthesized a novel core-shell nanosystem to demonstrate the feasibility of conducting Fischer–Tropsch syntheses by heating catalytic NPs with an external AMF. They produced monodisperse core-shell Fe@FeCo MNPs (12.2 ± 0.9 nm) and a thin, discontinuous Ru(0) layer supported on the initial Fe(0) MNPs (Fe@Ru) (12.4 ± 0.6 nm), which exhibited high SAR values of 284 and 190 W g_Fe_^−1^, respectively, under an AMF of 40 kA m^−1^ at a frequency of 54 kHz (Fig. 12A). The Fe@Ru MNPs showed slightly lower values than Fe@FeCo NPs at a similar particle size due to the reduced surface magnetization resulting from the Fe/Ru interface. The Fe@Ru MNPs achieves complete CO conversion with preferential formation of saturated hydrocarbons when exposed to H_2_/CO mixtures (4 bar, solvent-free), while Fe@FeCo MNPs favoured olefinic products. Importantly, the reaction started instantaneously upon field application and stops immediately when the field is switched off, illustrating the ultrafast thermal response and precise temperature control inherent to MI heating. This work established the conceptual foundation of “cold magnetic catalysis”, proving that induction heating can selectively activate high-temperature catalytic chemistry at the nanoscale while bypassing macroscopic heat-transfer limitations, and it directly inspired subsequent developments in induction-heated CO_2_ hydrogenation,^172–176^ steam methane reforming,^177,178^ and tandem catalysis.

CO_2_ methanation, or the Sabatier reaction converts captured CO_2_ and green hydrogen into methane and water. This exothermic process stores renewable energy in existing gas infrastructure and reduces emissions. Bordet and his team^167^ demonstrated the effectiveness of magnetically induced heterogeneous catalysis for CO_2_ methanation, presenting a strategically important method for intermittent energy storage and CO_2_ recovery (Fig. 12B). They developed 15 nm iron carbide MNPs (Fe_2.2_C) with exceptional heating capabilities, achieving SAR values up to 3000 W g^−1^ at 100 kHz and 37 kA m^−1^. Under an AMF, these iron carbide MNPs generated local heat, rapidly raising catalyst bed temperature from room temperature to 350 °C within 30 s, as monitored by infrared thermography, while the surrounding reactor remained comparatively cool. These iron carbide MNPs were utilized in the continuous flow hydrogenation of CO_2_ at atmospheric pressure, yielding CO and methane as the primary products. The team observed a significant enhancement in both activity and selectivity, with methane yields reaching 93% and selectivity surpassing 99% (22 kA m^−1^, 300 kHz) by anchoring iron carbide MNPs onto silica–alumina and using them as heat sources for small Ru NPs. This high level of performance was maintained for 150 hours of continuous operation without any loss in heating power or catalytic efficiency. They showed that MIH can be considered as a cornerstone technology for future power-to-gas and CO_2_ valorization process.

Steam methane reforming is the dominant, cost-effective industrial process for producing hydrogen by reacting high-temperature steam with methane (natural gas) over a catalyst. It converts methane into hydrogen and carbon monoxide, which in turn, react with more steam to increase hydrogen yield and carbon dioxide. Vinum and colleagues^168^ employed cobalt-nickel MNPs (CoNi) of approximately 35 nm, which were heated *via* MI, for steam methane reforming (Fig. 12C). These Co_0.5_Ni_0.5_ MNPs supported on γ-Al_2_O_3_ were heated to around 800 °C and showed activity in hydrogen production. Critically, magnetic characterization revealed exceptionally high Curie temperatures (*T*_c_ = 892 °C for CoNi and 875 °C for Cu-doped CoNi), enabling sustained magnetic hysteresis heating even above 800 °C, a regime inaccessible to iron-based susceptors. Under an AMF at 69 kHz with amplitudes up to 33 kA m^−1^, the catalyst bed rapidly reached reforming temperatures (715–750 °C equivalent equilibrium temperature), achieving 90–95% methane conversion at atmospheric pressure with stable operation for more than 300 h and no detectable catalyst degradation or carbon deposition. In this endothermic process, the rate at which the catalysts were heated using induction was limited by their chemical reactivity, whereas those heated through conventional methods were constrained by the efficiency of heat transfer.

CO oxidation is a critical catalytic process used to convert toxic carbon monoxide into carbon dioxide, essential for automotive catalytic converters, gas sensors, and air purification. Adogwa *et al.*^169^ demonstrated that the controlled localized heat generated by Fe_3_O_4_ (80–120 nm, synthesized *via* coprecipitation) under an AMF (field and frequency: 19 kA m^−1^, 205 kHz) effectively minimized unwanted side reactions and improved catalyst stability, even in harsh or dynamic operating conditions (Fig. 12D). They examined CO oxidation over Pt/Fe_3_O_4_ MNPs catalysts using both conventional thermal heating and MIH. As a result, MIH increased catalytic reactivity by more than 25 times compared to standard thermal heating. Kinetic analyses revealed distinct reaction orders and lower apparent activation energy in the MIH mode, while extensive post-reaction structural characterization (XRD, HAADF-STEM, XAS, XPS) confirms that the catalyst structure remains unchanged. *In situ* diffuse reflectance infrared Fourier transmission spectroscopy and isotope-exchange experiments further show that MIH selectively promotes the rate-determining *CO + *O → CO_2_ step by dynamically enhancing Pt–FeO_*x*_ interfacial synergy, enabling transient reaction pathways inaccessible under steady thermal conditions. This work suggests that magnetically induced catalysis constitutes a new catalytic operating mode, where AMFs can dynamically modulate surface electronic states and reaction energetics, thereby surpassing classical Arrhenius and Sabatier limitations and redefining the role of MIH concepts in heterogeneous thermal catalysis.

The conversion of ethylene directly into propylene through the two-step approach of ethylene dimerization into butylene followed by butylene reaction with ethylene *via* olefin metathesis to produce propylene represents a cutting-edge method for producing propylene. A variety of catalysts have been proposed to facilitate this reaction. Farpón *et al.*^170^ detailed the development of a multicomponent catalytic system that includes MNPs (25 nm CoFe_2_O_4_ NPs coated with SiO_2_, acting as a heating agent), thermometry functionalities (Tb- and Eu-doped Y_2_O_3_ NPs, approximately 300 nm, embedded in SiO_2_), an ethene dimerization catalyst (Ni NPs on aluminosilicate), and an olefin metathesis catalyst (Re NPs on Y-zeolite) (Fig. 12E). The temperature gradient produced by the locally heated MNPs was used to activate the nearby Ni and Re catalysts at distinct temperatures, facilitating the tandem dimerization (Ni catalyst at 137 °C) and metathesis (Re catalyst at 87 °C) of ethene into propene. Typically, these two reaction steps are conducted separately due to their incompatible temperature requirements. However, this work elevated MIH-based catalysis from an heating alternative to a tool for reaction orchestration, enabling tandem and multifunctional catalytic processes that are otherwise inaccessible using conventional thermal strategies.

Hydrogenation is a catalytic chemical process typically used to convert double or triple bonds of organic compounds into single bonds. Using metal catalysts like nickel, palladium, or platinum, it increases saturation, improving stability and altering textures in food production, petrochemicals, and pharmaceuticals. Hydrodeoxygenation is a critical catalytic upgrading process that removes oxygen from biomass-derived compounds (bio-oil) using hydrogen, typically at high temperature and pressure. MIH has emerged as a powerful strategy to overcome heat and mass transfer limitations inherent to conventional thermal catalysis, particularly for energetically demanding given transformations such as hydrogenation and hydrodeoxygenation.^179–186^ Mustieles Marin and colleagues^171^ synthesized bimetallic FeNi MNPs (of composition FeNi_3_ and FeNi_3_@Ni, approximately 16 nm) and employed them for magnetically induced hydrodeoxygenation and hydrogenolysis of biomass-model substrates in liquid phase (Fig. 12F). Reactions such as furfural and hydroxymethylfurfural hydrodeoxygenation, lignin-model ether cleavage, and cyclopentanone oligomerization proceeded efficiently under AMF excitation (300 kHz, 39 kA m^−1^), at low hydrogen pressures (3 bar) and without noble metals. Compared to conventional oil-bath heating at similar bulk temperatures, MIH delivered substantially higher conversion (97% instead of 60%) and better selectivity (37% instead of 8%), highlighting the critical role of nanoscale thermal gradients and catalyst surface overheating in activating high-bond dissociation energy pathways.

MIH-assisted catalytic reactions are not confined to the aforementioned categories. For instance, Chaudhuri *et al.*^187^ used MAGSILICA®, superparamagnetic core-shell NPs (Fe_3_O_4_@SiO_2_) onto which Au NPs were immobilized. When a fixed-bed PEEK flow reactor placed inside an induction coil, the magnetic core heated under the oscillating field (25 kHz), generating localized temperatures up to 120 °C at the catalyst surface. This magnetically induced heating enabled efficient oxidation of allylic and benzylic alcohols to aldehydes and ketones using molecular oxygen or air as the sole oxidant, achieving conversion in a single step with retention times of 30–60 min and minimal overoxidation. Compared with conventional oil-bath heating, the inductively heated system offered faster thermal response, safer handling of O_2_, improved heat-transfer efficiency, and straightforward scale-up.

In another work, Moura *et al.*^188^ showed that in induction-heated thermal catalysis, catalytic performance is governed not only by magnetic heating efficiency but also by interfacial ligand chemistry, which can critically regulate heat transfer from MNPs to reactants. The authors employed synthetically OA-capped Fe_3_O_4_ nanospheres (17–18 nm) produced by thermal decomposition. These NPs generated localized heat directly at the catalyst surface when exposed to an AMF (343 kHz, up to 48 kA m^−1^), enabling the oxidation of 1-octanol to octanal at lower bulk temperatures than required under conventional furnace heating. Selective oleic acid ligand removal using pyridine exchange, in contrast to ineffective TMAOH exchange, reduced surface ligand density by 2.5-fold, increased accessible surface area by more than 4 times, and enhanced SLP by a factor of 2.3 across all applied fields, resulted in a 2.5-fold increase in aldehyde yield under induction heating compared with untreated NPs. This work proved that optimizing surface chemistry is just as important as other parameters, such as magnetic anisotropy or particle size, for translating induction heating into effective thermal catalytic performance.

Beyond the representative case studies highlighted here, MIH has been successfully applied to a wide range of industrial catalytic transformations such as generating biodiesel, catalyzing and polyester depolymerisation.,^189–198^ indicating that magnetothermal activation constitutes a broadly applicable and scalable paradigm for thermal catalysis rather than a niche or reaction-specific approach.

## 4. Sustainable futures

Sustainable futures initiatives are rapidly gaining traction across all areas of science, offering new applications of existing technologies and importantly, creating disciplinary intersection in ways we haven’t seen previously. In MI, there are multiple use-cases for environmental science that we will explore in the following section.

### 4.1 Water electrolysis

Integrating MIH into water electrolysis presents a promising strategy to improve hydrogen production efficiency. Much like many of the other industrial applications we have discussed, electrolysis systems usually rely on bulk heating. These systems often see significant energy loss and slow reaction kinetics, especially for the oxygen evolution reaction, which is a significant bottleneck in water splitting. MIH instead leverages the locally generated heat offered by MNPs when exposed to an AMF to increase the temperature at the catalyst's active sites, reducing overpotentials and accelerating reaction rates without the need to heat the entire electrolyte volume.

Li *et al.*^203^ demonstrated induction-assisted alkaline water electrolysis using a thin ferromagnetic layer integrated at the electrode surface. The physical sputtering of a pure Fe film onto commercial Ni foam for 30 minutes yielded a continuous iron layer (thickness: 730 nm) that fully covered the Ni substrate serving as the inductively heated element. A DC-powered MOSFET circuit generated a high-frequency AMF (1.74 µT at the electrode), enabling rapid heating of Ni foam and Ni foam/Fe electrodes to ∼100 °C within 300 s and stabilization near 200 °C, with intermittent 60 s on/off cycling allowing controlled temperature modulation. This localized magnetothermal activation reduced hydrogen evolution reaction and oxygen evolution reaction overpotentials by 150 mV and 60 mV at 20 mA cm^−2^, respectively, increased hydrogen evolution reaction current six-fold at −1.4 V, and enhanced overall water-splitting current by 540% under intermittent induction. Density-functional calculations attributed the performance gain to lattice expansion of Fe and Ni under induction heating, which optimizes absorbed hydrogen adsorption/desorption energetics.

On the other hand, localized nanoelectrocatalyst heating provides an alternative to electrode-scale induction. FeC–Ni core-shell MNPs have been used by Niether C, *et al.*^199^ to exhibit significant improvements in oxygen evolution reaction activity under AMF, with enhanced current densities and reduced energy input compared to conventional heating approaches (Fig. 13A). They used 15 nm FeC@Ni core–hell NPs, synthesized by carbidization of Fe(0) MNPs under syngas to form crystalline Fe_2.2_C cores, followed by chemical growth of a Ni shell *via* thermal decomposition of Ni(acac)_2_ in the presence of palmitic acid. The FeC core provided exceptionally high magnetic heating power, while the Ni shell served as the electrocatalytic surface for hydrogen and oxygen evolution. Under an AMF generated at 300 kHz, with field amplitude tunable from 8.5 to 60 kA m^−1^, the FeC–Ni MNPs exhibited specific absorption rates up to 2 kW g^−1^ at 37 kA m^−1^, among the highest reported for metallic carbide nanoheaters. Despite only a minor bulk electrolyte temperature rise of 1.5–3.5 °C, the local NP surface temperature was estimated to reach ∼200 °C, close to the Curie temperature of the Fe_2.2_C core. This nanoscale heating lowered OER overpotential by 200 mV at 20 mA cm^−2^ and reduced full-cell voltage by 100 mV at 70 mA under 38 kA m^−1^ AMF. These results show that MIH can selectively heat catalytic nanostructures to accelerate water splitting while maintaining low bulk temperature and limiting corrosion. Note that, in spite of various efforts by different groups, prediction of the surface temperature of a MNP as a function of the energy spent is an extreme challenge and has not been clearly achieved yet.^199^

**Fig. 13:**
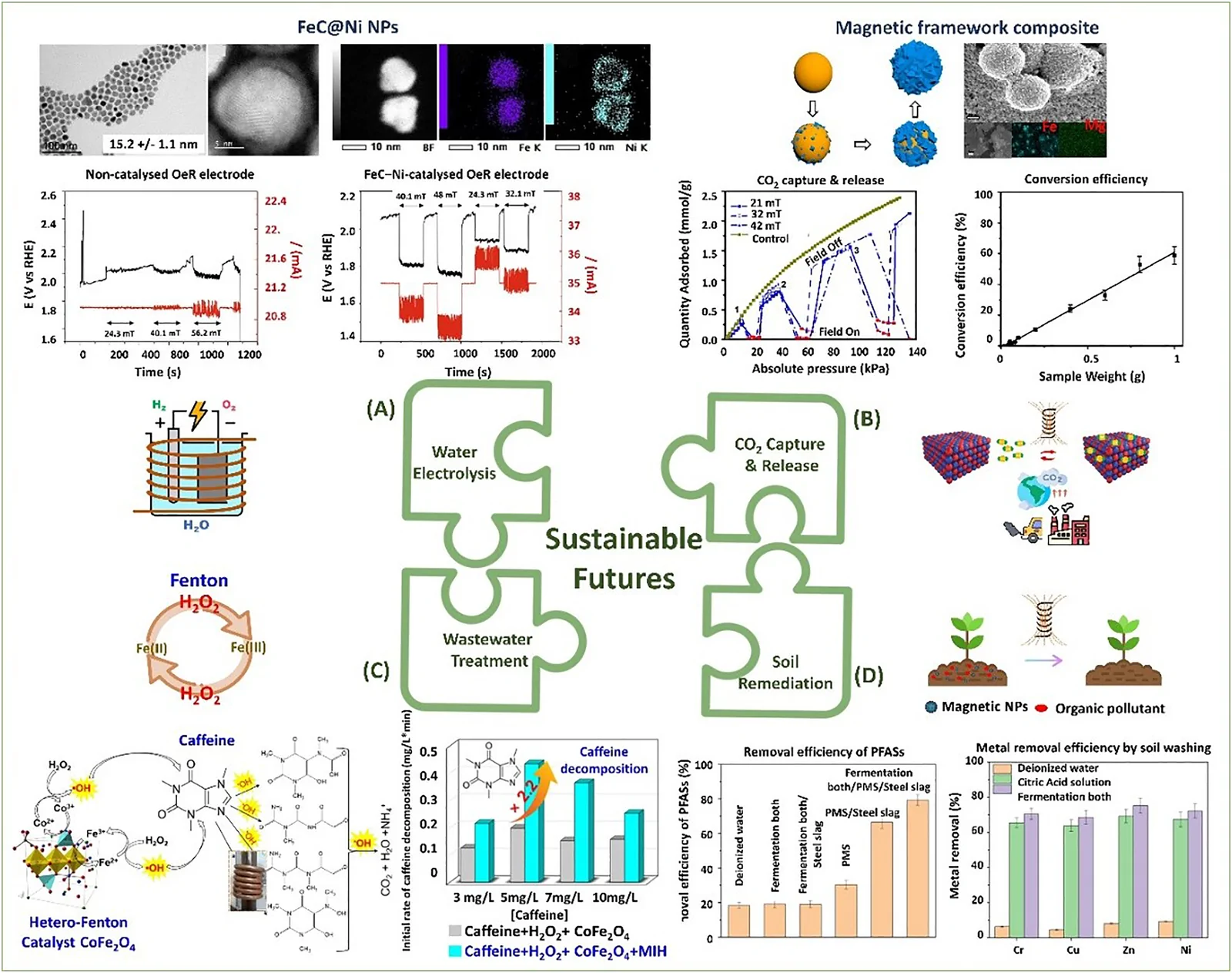
MIH application for sustainable future to (A) improve water electrolysis through MIH of FeC@Ni core-shell NPs [adapted/reproduced from ref. 199 with permission from Springer Nature,^199^ Copyright 2018]. (B) Fast thermal regeneration of magnetic framework composite (UiO-66 MOF) sorbents under induction heating used for temperature swing adsorption (TSA) process to enhance CO_2_ capture applications [adapted/reproduced from ref. 200 with permission from American Chemical Society,^200^ Copyright 2016]. (C) Cobalt ferrite used as a hetero-Fenton catalyst for wastewater treatment [adapted/reproduced from ref. 201 with permission from Elsevier,^201^ Copyright 2023]. (D) Electrokinetic *in situ* chemical oxidation system to restore contaminated soil and remove high concentrations of heavy metals (Cr, Cu, Zn and Ni) and fluorinated compounds (PFAS) through MI [adapted/reproduced from ref. 202 with permission from Elsevier,^202^ Copyright 2023].

Magnetothermal heating can also enhance catalysis through magnetic-state transitions rather than external AMF excitation. Lu *et al.*^204^ developed ferro–antiferromagnetic core–shell NiFeN@NiFeOOH electrocatalysts in which temperature-driven magnetic transitions reduce spin-dependent electron-transfer barriers during OER. Crystalline NiFeN cores (∼50 nm) formed by coprecipitation and nitridation were coated *in situ* with 5 nm amorphous NiFeOOH during electrochemical activation. The NiFeN core exhibited a ferromagnetic–paramagnetic transition at 55 °C, while the NiFeOOH shell showed an antiferromagnetic–paramagnetic transition near 70 °C, confirmed by SQUID and ZFC/FC analysis. Crossing these transition temperatures introduced spin disorder that lowered barriers for Ni^2+^/Ni^3+^ redox cycling and OER intermediate oxidation. Heating from 40 to 90 °C reduced OER overpotential from 1.49 to 1.39 V at 10 mA cm^−2^ and decreased Ni^2+^/Ni^3+^ activation energy from 12.7 to 4.2 kJ mol^−1^. Above 70 °C, the paramagnetic NiFeOOH shell additionally enabled thermochemical water oxidation by Ni^3+^ species, further accelerating oxygen evolution. These results show that magnetic-state-controlled heating and spin reconfiguration provide an alternative to bulk thermal management, enabling high-temperature kinetics without globally heating the electrolyte. The synergy between MIH and electrocatalysis offers a pathway towards next-generation water-splitting technologies.

### 4.2 CO_2_ capture

In 2023, global CO_2_ emissions were estimated at 36 billion tons, posing a significant threat to our planet's health.^205^ The scientific community, along with governments and industries, is striving to find solutions to this challenge. Carbon capture and storage (CCS) is increasingly recognized as a viable approach to significantly reduce global greenhouse gas (GHG) emissions. To address this issue, the electrification of chemical processes is being explored to decarbonize and lower GHG emissions.^206^ By utilizing electricity to drive chemical processes, it becomes possible to implement either direct electrochemical transformations or convert electrical energy into heat.^207^ Electricity can supply the thermal energy required in many chemical reactions through various electromagnetic sources such as microwave, induction, and resistive heating. Specifically, CO_2_ produced in a chemical process can be removed *via* an electrified carbon capture process.

Common CCS processes rely on porous adsorbent materials or chemical absorption. However, these solutions are not yet feasible for large power plants because regenerating the porous adsorbent *via* pressure swing adsorption and temperature swing adsorption (TSA) are energy-intensive processes, as is the regeneration of amine-based solvents. This makes their application impractical, as the benefits achieved would lead to an increase in CO_2_ emissions due to the significant rise in energy demand.^200,208,209^ Therefore, it is crucial to pursue an efficient solution with minimal energy impact. Among recent studies, magnetic induction swing adsorption (MISA) consists of a temperature-swing adsorption process in which an AMF directly heats a magnetically responsive adsorbent, enabling rapid *in situ* desorption and adsorbent regeneration, has emerged as a promising technique to enhance the energy efficiency of gas separation.^200^

MISA employs composite materials consisting of an adsorptive component combined with MNPs. These NPs generate heat locally when exposed to AMFs, enabling rapid and efficient regeneration of the sorbent. This localized heat release, directly in contact with the adsorbent material, eliminates thermal transport limitations and thermal losses, resulting in faster regeneration cycles and increased system productivity compared to external heating methods. In this scenario, two main categories of composites for MISA can be identified: porous types, used as dry filtering materials, and those colloidally dispersed in a liquid matrix.^210^ In the first category, MNPs are embedded in a porous matrix that traps CO_2_ molecules, and the heat generated by the NPs under induction is used to release the gas. Gholami, Mohsen, *et al.* recently demonstrated the capabilities of a composite material made of commercially available Fe_3_O_4_ NPs embedded in a 13X Zeolite as a sorbent matrix for MISA. They found that the heat generated in the sorbent is directly proportional to the power of the applied AMF and the concentration of MNPs. For example, a SAR value of 150 W g_Fe_^−1^ was obtained for the magnetic composite when exposed to the AMF. This results in a significant improvement in the regeneration of the adsorbent material by controlling the energy required for gas release; however, it also leads to a reduction in the active surface area of the zeolite as the concentration of NPs increases, thereby decreasing its adsorption performance.^207^

Similar findings were previously described by Lin, Xiaoqing, *et al.*, who utilized Fe_3_O_4_ NPs (average size: 50 nm, amorphous) embedded in N-doped porous carbon for the same application. When exposed to an AMF (30 kHz and 0.15 kA m^−1^), a SAR value of 72.5 W g_Fe_^−1^ was obtained.^211^

After optimization, *in situ* electromagnetic induction heating for TSA at 110 °C exhibited an excellent performance with the desorption rate of 3.27 mg g^−1^ s^−1^ and the energy efficiency of 79.2%, consuming 18% less energy than conventional TSA under identical conditions. Therefore, the highly efficient *in situ* EMIH technology provided a promising alternative way for CO_2_ capture and could be applied in a wide variety of traditional combustion-related areas. In this two-case study, they demonstrated the advantages of using MISA for CCS. Notably, aside from the reduction of sorbent surface relative to NP concentration, both systems exhibited a regeneration speed one order faster than standard TSA processes and superior desorption performance, with a desorption rate exceeding 3.27 mg g^−1^ s^−1^ and energy efficiencies above 70%.^207,211^

The second class of composite materials is represented by MNPs, on which surfaces, in most cases, a metal-organic framework (MOF) shell or other sorbent material has been grown. Sadiq *et al.* reported that MgFe_2_O_4_-based MOF, when subjected to an AMF, produced heat (SAR = 55.4 W g_Fe_^−1^) (Fig. 13B). Impressively, this method resulted in the complete liberation of 100% of the adsorbed CO_2_ when exposed to a 33 kA m^−1^ field.^200^ In another study, Bellusci *et al.* concluded that a SAR value of 1.6 W g^−1^ of composite can be obtained when the Fe_3_O_4_@HKUST-1 composite is exposed to a frequency of 190 kHz and a field of 10 kA m^−1^. These types of composites are overcoming the issue related to the decrease of available sorbent surface. Indeed, MNPs serve as the cores of the composite, and the surface of the sorbent material is fully available to bind gas molecules.^210^

Moreover, they have been utilized as a colloidal solution, with the diffusion of gases through the aqueous matrix facilitating CO_2_ absorption. The thermal performance of the composite's magnetic core demonstrated a swift and rapid temperature change, ensuring complete CO_2_ removal with an energy conversion efficiency exceeding 60%. For this type of composite, the thermal performance of the NPs is crucial to achieving the desired outcome. The MNPs used in these studies are mostly commercially available and do not reflect recent advancements in heating and magnetic performance (SAR values of these MNPs are below 200 W g_Fe_^−1^), a threshold that the new generation of MNPs, at the same concentrations and AMF conditions, has surpassed. This suggests that the performance of the MISA could be even higher, achieving more impressive energy efficiency values.

### 4.3 Wastewater treatment

MNPs have also emerged as effective materials for removing heavy metals from contaminated water. Their high surface area and magnetic responsiveness enable efficient adsorption of metal ions and rapid magnetic separation from solution. The process involves dispersing functionalized MNPs into contaminated water, where they bind heavy metal ions through different types of interactions such as electrostatic attraction or ion exchange. Once adsorption occurs, a static external MF can aggregate and remove the MNPs together with the bound contaminants, delivering purified water.^212^ In perhaps the most compelling example for this approach, a study conducted by Almomani *et al.*^213^ the application of MNPs was examined for the removal of heavy metals (Ni, Cu, and Al) from secondary effluent industrial wastewater. The authors synthesized hyperbranched polyglycerol-grafted iron oxide magnetic NPs (HPG-MNPs) and applied them to remove Cu^2+^, Ni^2+^, and Al^3+^ from real wastewater samples. Using magnetic separation, 99.8% of particles were recovered in 35 s using an external magnet. The adsorption performance reached high capacities (Langmuir *q*_max_ ≈ 0.700, 0.451, and 0.790 mg for Cu, Ni, Al, respectively), with best removal at pH 9, and the sorbent remained reusable for 12–15 adsorption/desorption cycles depending on the metal. A similar approach was demonstrated by Yavuz *et al.*,^214^ who used monodisperse Fe_3_O_4_ nanocrystals (4 and 12 nm) to achieve rapid arsenic removal under low magnetic field gradients (<100 T m^−1^). In contrast to the previous paper, this study focused on trace-contaminant purification in natural water sources, further confirming the versatility of magnetically recoverable adsorbents for diverse water-treatment scenarios.

In 2019, Rivera *et al.* showed that MIH can intensify heavy-metal removal more effectively than conventional bulk heating by creating nanoscale ‘hot spots’.^215^ SPIONs (12 ± 4 nm, flow-synthesized) were heated MIH (222 kHz, 350 A), raising a 2 g L^−1^ suspension to 60 °C. Under these conditions, adsorption capacity increased from 6.6–6.9 mg g^−1^ at room temperature to 11.9–12.4 mg g^−1^, whereas conventional water-bath heating at 60 °C yielded only 7.8–8.2 mg g^−1^. Induction heating also reduced processing time dramatically, with 5 min under MIH exceeding the 2 h performance of conventional heating. XPS analysis revealed enhanced Cr(vi) → Cr(iii) surface reduction, indicating synergistic adsorption–redox behaviour driven by localized magnetothermal heating. Furthermore, advanced oxidation processes, particularly Fenton and Fenton-like reactions, were proven to be effective in degrading organic pollutant in wastewater, yet their industrial implementation remains hindered by mass-transfer limitations, slow reaction kinetics, and difficulty in catalyst recovery. Han Chen's group employed MIH to increase degradation efficiency of organic pollutant in aqueous solution and proved that the process was energy-saving, including catalytic degradation, catalyst regeneration, and Fischer–Tropsch synthesis.^216^ They extended this technology to a common wastewater treatment process known as catalytic wet peroxide oxidation (CWPO). Energy-balance calculations showed that although the outlet water temperature stabilized near 57 °C, the catalyst surface temperature reached 230 °C (more than 170 °C hotter than the bulk liquid). This localized magnetothermal activation dramatically enhanced reaction kinetics such that CWPO of azo dyes achieved more than 92–98% removal efficiencies within 30 minutes, compared with only 20% removal under conventional bulk heating at the same liquid temperature of 63 °C.

Another work performed by Rivera's group^217^ published in 2020 demonstrated MIH-intensified Fenton-like degradation of organic dyes for wastewater treatment. Conventional heating systems are limited by slow heat transfer from the bulk liquid to the catalyst surface, which delays radical generation and increases energy demand. To address this, the group used SPIONs (21 ± 7 nm, produced by continuous electrochemical flow synthesis) as self-heating heterogeneous Fenton catalysts. Under AMF exposure (222 kHz, 14–24 kA m^−1^), 2 g L^−1^ suspensions rapidly reached 70–90 °C, while the MNP surface temperature exceeded the bulk liquid, forming interfacial magnetothermal hot spots. This localized heating enabled complete discoloration of 100 ppm methylene blue within 90 min, compared with only 67% conversion under conventional heating at the same bulk temperature. Total organic carbon mineralization increased from 47.8% (water bath) to 52.3% (induction heating), and the apparent pseudo-first-order rate constant rose by nearly two orders of magnitude. These results provide direct experimental evidence that magnetothermal nanocatalysts create high-temperature interfacial microreactors that overcome kinetic and energy limitations of conventional advanced oxidation processes while also enabling magnetic recovery and reuse of the catalyst.

Tatarchuk *et al.*^201^ prepared CoFe_2_O_4_ hetero-Fenton catalysts by continuous flow co-precipitation and activated them inside a water-cooled 6-turn copper induction coil operated at 100 kHz (31.4 V, 7.86 A) (Fig. 13C). Induction heating accelerated heterogeneous H_2_O_2_ decomposition by up to 2-fold, with the largest acceleration observed at higher catalyst loading (notably 3 g L^−1^), and the apparent kinetics also depended on H_2_O_2_ concentration. In a target-contaminant test of caffeine oxidation (CoFe_2_O_4_ MNPs heated at 400 °C); (3 g L^−1^, pH 6.5, V = 40 mL), caffeine removal increased from 40% to 85%, while initial caffeine and H_2_O_2_ depletion rates increased by 1.6–2.3 fold and 1.1–2 fold, respectively. These results establish field-tunable control of heterogeneous oxidant activation, without changing bulk reactor hardware or relying exclusively on conventional external thermal ramps.

Magnetically driven oxidation has also been demonstrated at reactor-relevant scales using structured inductive catalysts and engineered multicore nanoheaters. Gallo-Cordova A, and co-workers^218^ developed a self-heating magnetic nanocatalyst (MNC) specifically engineered for MIH-enhanced advanced oxidation processes of real landfill leachate and textile wastewater. They used uniform multicore iron-oxide NPs synthesized by solvothermal polyol method. TEM showed aggregates of ∼10 nm maghemite/magnetite cores assembled into 40 nm multicore particles, providing high magnetic moment per particle and colloidal stability. SAR values reached 850 W g_Fe_^−1^ at 200 kHz and 17 kA m^−1^ such that the NPs heated water to 90 °C within 5 min at a suspension concentration of 1 mg mL^−1^, demonstrating promising self-heating catalytic behaviour. When applied to Fenton-like degradation of real wastewaters, AMF-driven magnetothermal activation produced faster reaction kinetics than conventional heating at the same bulk temperature. Degradation and mineralization yield of the organic pollutants for landfill leachate were measured by chemical oxygen demand COD and TOC and reached 90% and 75%, respectively, under AMF compared with conventional heating at 90 °C. Because heat is generated at the catalyst surface, a temperature gradient exists between MNP interfaces and the bulk solution, boosting reactive oxygen species production and accelerating pollutant oxidation. Additionally, the multicore particles exhibited strong magnetophoretic mobility, enabling rapid magnetic recovery and reuse, making this study a benchmark demonstration of synthetic iron-oxide nanocatalysts functioning simultaneously as adsorbents, Fenton catalysts, and magnetothermal nanoheaters for scalable wastewater treatment.

The same team^219^ engineered two architectures including single-core particles (NP15) (mean diameter of 15 ± 3 nm), and multicore nanoflower clusters (NF60) (mean diameter of 60 ± 8 nm composed of 7 nm crystalline cores) as self-heating nanocatalysts for wastewater remediation through MIH-enhanced Fenton-like reaction. Cubic spinel iron oxides MNPs (Fe_3_O_4_ and γ-Fe_2_O_3_) were stabilized by residual polyol surface ligands. Under an AMF, their heating efficiency was quantified by SAR measurements such that NP15 reached the SAR value of 326 W g_Fe_^−1^ at 200 kHz and 24 kA m^−1^, while NF60 reached 144 W g_Fe_^−1^ under the same conditions, reflecting weaker magnetic coupling in multicore structures. For catalytic degradation of organic dyes (acid orange 8 and methylene blue), an AMF of 200 kHz and 16 kA m^−1^ was selected, enabling rapid local surface heating. The solution temperatures rose to 50 °C (NP15) and 35 °C (NF60) within 2 h at 1 g L^−1^ iron concentration, while higher concentrations (2–3 g L^−1^) allowed heating to 90 °C, achieving complete dye decolorization. These results demonstrate that synthetically engineered MNP nanoheaters produced by microwave polyol synthesis can serve as efficient MIH-activated catalysts for wastewater treatment, with performance governed by NP size, architecture, and AMF parameters.

Recently published work by this group reported magnetothermally active nanocatalyst system for the magnetic harvesting and degradation of polyethylene microplastics using iron oxide nanoflowers produced by a scaled-up solvothermal polyol process.^220^ Nanoflowers were multicore particles (33 ± 4 nm overall diameter) composed of 8 nm magnetite/maghemite cores, with inverse-spinel structure. The SAR value of 380 W g^−1^ obtained under an AMF (at 100 kHz and 48 kA m^−1^). In the degradation stage, microplastics were first hydrolyzed at 150 °C, then subjected to Fenton-like oxidation catalysed by magnetic particles in the presence of H_2_O_2_. Under conventional heating, mineralization increased with bulk temperature, reaching 75% at 90 °C; however, when an AMF of 100 kHz and 48 kA m^−1^ was applied, the bulk liquid remained at only 60 °C, yet mineralization rose to 78%, evidencing strong magnetothermal hot-spot formation at the NP surface. These interfacial temperatures enhanced ROS generation and reaction kinetics without heating the entire reactor volume. In parallel, the high magnetic moment of the NFs enables rapid magnetic harvesting of microplastics with capacities up to 1000 mg_microplastic_ g_nanoflower_^−1^ and full reusability over multiple cycles.

Filter-membrane processes are widely employed for drinking-water purification and wastewater treatment because semi-permeable membranes can efficiently separate suspended solids, microorganisms, and dissolved contaminants. However, a persistent limitation of these systems is biofouling, arising from the accumulation and growth of bacteria and organic matter on membrane surfaces and within pores. Biofouling progressively blocks permeation pathways, lowers flux, degrades separation efficiency, and ultimately renders membranes unusable. Current mitigation strategies rely on periodic chemical cleaning or physical replacement of membranes, both of which interrupt operation, reduce throughput, shorten membrane lifetime, and significantly increase operational costs.

Anvari *et al.*^221^ introduced an “self-heating” vacuum membrane distillation platform to address a core bottleneck in water desalination, temperature polarization and heat loss due to low permeate flux in conventional vacuum membrane distillation. Their solution was a dual-layer composite membrane where the heating agents are embedded directly at the membrane surface. A hydrophilic, thermally conducting Fe-CNT layer was spray-coated onto a commercial hydrophobic PTFE membrane (0.2 µm pores). Induction heating was provided by an UltraFlex SH-2 system at a fixed 283 kHz. The static tests showed that a 30 µm Fe-CNT layer (23 g m^−2^ loading) can produce rapid interfacial heating (water droplet evaporation in ∼20 s; heating rate 7 °C s^−1^), while in flow setup achieved 4–5 kg m^−2^ h^−1^ flux with 99% salt removal. Results indicate that the MIH-VMD system has a lower specific energy (197 Wh kg^−1^) in comparison to the conventional VMD (1202 Wh kg^−1^). The attained specific heating energy of the MIH-VMD system is lower than some values reported in the literature on conventional MD systems. Moreover, the calculated values of gain output ratio (GOR) which defines the ratio of the water evaporation energy to the energy input of the system, show a high energy efficiency of MIH-VMD (3.45) respect to solar irradiation (0.78) and ohmic heating (0.54).

In addition, Nguyen H, *et al.*^222^ reported work done to remove biofoulants through MIH. To mitigate membrane fouling, various strategies have been implemented, including feed water pretreatment, optimization of operating parameters, membrane cleaning procedures, and the development of antifouling membrane coatings. Nevertheless, these approaches are unable to fully eliminate the issue. In particular, feed water pretreatment necessitates additional treatment units, such as flotation and activated sludge tanks, which require extra space and energy consumption while also increasing operational costs. In this work, the commercial glass-fiber microfiltration membranes were coated with magnetite (Fe_3_O_4_) MNPs of 50 nm diameter, which were further functionalized with dextran (DX6) to improve dispersion and hydrophilicity before deposition onto the membrane surface. Using a DM100 AMF system operating at 604 kHz and 24 kA m^−1^, a 15 mg DX6-functionalized Fe_3_O_4_ achieved rapid magnetothermal heating. The Box–Lucas simulations and thermal measurements showed that surface temperatures reached 180 °C within 300 s, with a heating rate of 1.03 °C s^−1^, while maintaining uniform temperature distribution across the entire membrane area. This localized high-temperature induction heating thermally decomposed bacterial biofilms (Bacillus subtilis and environmental biofoulants) within less than 3 minutes, restoring membrane permeability without damaging the underlying polymer or glass substrate. This study suggests a rapid, chemical-free, and remotely controllable strategy for membrane revival, offering a scalable solution to biofouling in wastewater treatment systems.

### 4.4 Soil remediation

Soil pollution is a significant environmental and health issue, with global implications for the health of humans and ecosystems.^223^ The accumulation of toxic metals and metalloids in soil is attributed to a wide range of causes led by human activity such intensive agriculture, industrial manufacturing, mining and landfill sites. From a health perspective, heavy metals such as cadmium present a significant risk in high doses and even at lower exposures have serious implications, with links such as cardiovascular disease.^224^ For food supply, it is estimated that 78% of the average per capita calorie consumption worldwide comes from crops grown directly in soil.^225^ Techniques to remove contaminants, known as soil remediation, can therefore be considered as an critical research priority, with progress aiming to focus on net-sustainable avenues.

Within this context, thermal remediation approaches are a key focus in this contest.^226^ Soil remediation of organic contaminants commonly relies on thermal desorption, chemical oxidation, or reductive treatments to mobilize or destroy pollutants. In heterogeneous soils, externally applied heat spreads unevenly and rarely reach contaminant-rich microenvironments, making treatment slow and high in energy consumption. Embedding magnetically responsive materials within the soil instead creates internal heat sources under an AMF, delivering thermal energy directly at contaminant sites. This enables faster volumetric heating and more effective desorption or reaction without large bulk temperature gradients.

In a work by Chen *et al.*,^202^ they developed a MIH-assisted electrokinetic *in situ* chemical oxidation system by coupling persulfate oxidation with inductive heating of iron-rich steel slag distributed within the soil matrix (Fig. 13D). The steel slag composed mainly of bulk materials Fe_3_O_4_ (31 wt%), Fe_2_O_3_ (22 wt%), CaO, MnO, and graphite and can be used simultaneously as a low-cost heterogeneous catalyst and magnetic susceptor under AMF. Under an AMF (200 kHz, 15 kA m^−1^, 0.6 kW), inductive heating raised soil temperatures locally from 26 °C to 55–56 °C within 1 h, accelerating persulfate decomposition into SO_4_˙^−^ and ˙OH radicals, enhancing steel slag corrosion. This magnetically driven activation substantially improved oxidant transport and reaction rates, yielding simultaneous removal of 86.7% of per- and poly-fluoroalkyl substances (PFASs) and 87.2% of heavy metals after 15 days, reaching the final soil meeting residential safety standards. The outcome suggests that integrating MIH with iron-based activators can overcome mass-transfer and kinetic barriers in soil remediation, enabling rapid and uniform treatment of complex contaminated soils.

To deliver uniform heat into heterogeneous soil volumes, Kim *et al.*^227^ proposed a novel soil-heating strategy using MNP-coated geotextiles. Instead of heating the soil bulk, the authors embedded magnetically responsive heat sources directly inside the soil matrix. The chosen MNPs were SPIONs coated *in situ* onto commercial polypropylene geotextiles by chemical co-precipitation (<10 nm) with superparamagnetic behaviour (*M*_s_ = 4.36 emu g^−1^, negligible remanence). Under AMF exposure (332 kHz, 9.8 A, 210 V), the coated textile reached 60 °C within 1 min, confirming efficient magnetothermal conversion. When embedded in dry sand or clay columns, the textile generated immediate internal soil heating, increasing surface temperature by 6–10 °C within 300 s, whereas uncoated controls showed no change. Inverse heat-transfer analysis estimated 40 W local heating power, and simulations indicated the coating could reach 200 °C within 300 s without surrounding soil, demonstrating effective heat transfer into the soil matrix. Rough, non-woven geotextiles retained more MNPs and produced higher heating rates than smooth woven fabrics, revealing textile architecture as a control parameter for soil heating.

Phenrat *et al.*^228^ presented a definitive proof-of-concept study demonstrating MI-assisted remediation of chlorinated contaminants in soil and groundwater. The slow desorption and mass-transfer limitation of sorbed contaminants is a central bottleneck in Fe(0) remediation, which restricts reaction rates in conventional Fe(0) treatments. The authors employed commercial ferromagnetic Fe(0) powders and commercial nanoscale Fe(0) as self-heating reactive media. Under a low-frequency AC EMF (*f* = 150 kHz, current = 15 A) generated by a custom induction coil, Fe(0) and nanoscale Fe(0) suspensions (10 g L^−1^) exhibited rapid magnetothermal heating, reaching 80–123 °C within 15–20 minutes, whereas soil or water without iron particles showed no measurable heating. Heating kinetics constants (*k*_HI_) reached 9.4 × 10^−2^ min^−1^ for ZVI, yielding Δ*T*_max_ = 109 °C, while nanoscale Fe(0) achieved Δ*T*_max_ = 64 °C in deionized water. When test was performed on trichloroethylene (TCE)-contaminated soil-groundwater slurries, the induction-heated nanoscale Fe(0) produced a 5.36-fold enhancement in dechlorination rate compared with no field application. The mass-normalized pseudo-first-order rate constant increased from 2.20 × 10^−3^ to 11.80 × 10^−3^ L g^−1^ h^−1^, enabling complete destruction of sorbed TCE within 31 h, whereas nanoscale Fe(0) treatment without MIH was stopped due to slow desorption. Control experiments using water-bath heating to the same bulk temperature achieved only 1/3 of the reaction rate observed under AMF, confirming that magnetically induced local heating accelerated ZVI corrosion resulted in intensified interfacial reaction kinetics beyond what bulk thermal treatment can deliver.

Similarly, Yu *et al.*^229^ reported a comprehensive mechanistic study demonstrating magneto-thermally enhanced soil remediation by coupling ferromagnetic Fe(0) particles with a low-frequency EMF to drive thermal desorption of volatile organic contaminants. In this system, the commercially available ZVI particles millimetric Fe(0) (1–2 mm) and micrometric Fe(0) powders (100-mesh and 400-mesh) used together with commercial Fe_3_O_4_ and α-Fe_2_O_3_. Soil mixed with Fe(0) was placed inside a custom induction coil and was exposed to an AMF. The temperature of soil sample with Fe(0) particles rapidly raised to 60 °C within 20 min, whereas soil without Fe(0) showed negligible heating. After 60 min of AMF treatment, the residual fraction of *m*-xylene in soil decreased by 86.4% in the presence of 1–2 mm Fe(0) compared with untreated soil. The highest heating capacity occurred at soil pH 5, where preservation of metallic Fe^0^ and Fe(ii) species was maximized. Electrokinetic measurements further showed that lower pH reduces electrostatic repulsion between ZVI and fulvic acid-bound contaminants, improving thermal conduction between heater and pollutant. Collectively, this work established that commercial Fe(0) particles under low-frequency AMF can serve as robust magnetothermal agents, enabling volumetric heating within soil matrices, overcoming the heterogeneous heat-transfer limitations of conventional thermal desorption for soil remediation.

A systematic comparison of the reported articles reveals that the remediation efficiency strongly depends on the magnetic material composition, particle geometry, and coupling mechanism between heating and contaminant degradation. Steel-slag-based systems coupled with persulfate oxidation achieved the highest simultaneous multi-contaminant removal efficiencies (≈87% for PFASs and heavy metals), highlighting the advantage of combining MIH with catalytic radical generation pathways. Fe(0)-based systems primarily excelled in accelerating reductive dichlorination and thermal desorption processes, producing up to 5.36-fold faster degradation kinetics for chlorinated compounds compared with non-heated controls. Meanwhile, MNP-coated geotextiles demonstrated superior heat-distribution capability and operational simplicity for *in situ* deployment, although their remediation performance was evaluated mainly from a thermal-transfer perspective rather than direct contaminant destruction. Collectively, these studies indicate that MIH platforms integrating both efficient magnetothermal conversion and catalytic reactivity provide the promising route toward remediation of heterogeneous contaminated soils.

### 4.5 MI to generate motion

Nowadays, research in different fields such as biomedicine is leaning towards the automation of processes. A focal point for this research is the autonomous movement of objects in hostile environments with limited space, with the human body providing a perfect example of this territory. With this in mind, an interesting field of study is presented by the development of nano- and micro- particles capable of autonomous propulsion, known as “nano- and micro-motors”, which we have discussed in places throughout this review. These particles have created new opportunities, in particular for biomedical applications in which precision and invasiveness are a topic of primary importance.^230^ They can fulfill different tasks, ranging from targeted drug delivery, diagnostic, cancer treatment to microsurgery.^231^ Based on the propulsion mechanism,^232^ they can be divided into two main categories: First, the catalytic particles, in which the movement is obtained *via* chemical reactions. A “fuel” (*i.e.* H_2_O_2_, N_2_H_4_) is consumed to generate propulsion through the formation of chemicals gradients or gas bubbles. Secondly, the physically driven particles, which are propelled by external physical stimuli which include light, MF, electric field and their combinations.^233^ Given the need to orient them autonomously on a path, one of the main problems is the directionality of these objects, often resized using asymmetric particles that in many cases combine chemical reactions and propulsion by physical stimuli. In this scenario, the use of static or rotating MFs in combination with magnetic particles has been extensively reviewed due to the possibility of orienting the particles while providing different drag forces.^234^

However, there is only a single study based on MNPs, which was reported by Baraban *et al.*,^235^ presenting a fuel-free method for driving microscale locomotion by utilizing MI-based thermophoresis rather than other type of field types (Fig. 14). The core of this mechanism relies on the asymmetric structure of Janus motors, which consist of 3 µm silica spheres partially covered with a 100 nm thick Permalloy (Py, Fe_19_Ni_81_ alloys) magnetic cap. Locomotion is triggered when an AMF (4 kHz, 6 kA m^−1^) is applied to the solution, causing the metallic cap to absorb electromagnetic radiation and undergo hysteretic losses. This MIH generates localized heat specifically on the capped side of the particle, while the rest of the silica body remains relatively unaffected. The resulting temperature difference between the two sides of the particle, estimated to be about 2 K, creates a microscopic gradient that drives the motor *via* a self-phoretic slip. This localized heating on one side allows the motors to reach velocities of approximately 0.34 µm s^−1^, which is an order of magnitude faster than the random Brownian motion of non-magnetic reference particles. Furthermore, the 100 nm thickness of the Permalloy film ensures a stable magnetic vortex state, which prevents particle clumping and allows for precise steering through an additional static MF. By focusing the heating on a single side through MIH, this thermophoresis-based approach provides a biocompatible alternative to chemical fuels and offers the unique advantage of working in non-transparent environments where laser-based heating would fail.

**Fig. 14:**
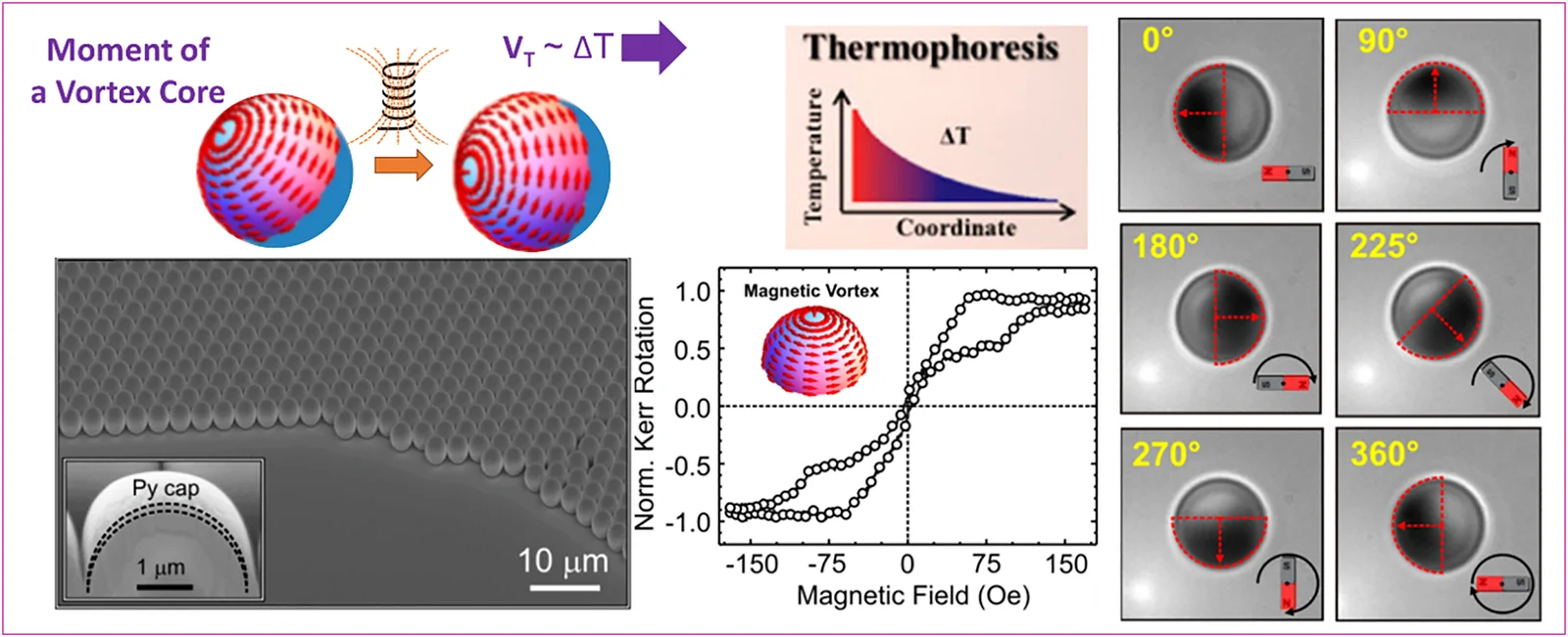
Example of the only original work of MIH application for locomotion. Propulsion of the particles by thermophoretic force when exposed to an AMF [adapted/reproduced from ref. 235 with permission from American Chemical Society,^235^ Copyright 2013].

A different approach to obtain movement is represented by the levitation and wireless manipulation of room-temperature liquid metals (LMs), such as gallium-indium eutectic alloys (EGaIn).^86^ Unlike the asymmetric Janus magnetic motors that rely on heat dissipation through hysteresis losses, LMs dissipate heat through Joule effect. Indeed, when conductive materials such as LMs are exposed to a time varying MF, it leads to the formation of eddy currents within the material which results in the release of heat.^236^ Recent research has demonstrated that bulk LM can be not only heated but also controlled in a non-contact manner using AMF (245 kHz, 250 A m^−1^).^237^ These techniques enable three-dimensional motion, including locomotion and electromagnetic levitation, which significantly surpass the capabilities of traditional two axis electrical or redox-based methods. The high mobilities and the unique physical properties of LMs, specifically their inherent softness, flexibility, workability and heating properties makes them interesting for targeted cancer elimination and the operation of smart microdevices. Given the limited number of published works on these complex and new materials, there is room for rapid development of the topic that can revolutionize the world of nanomedicine.

## 5. Discussion and perspectives

### 5.1 Perspectives in biomedicine

Oncological applications have dominated MIH research for decades, with significant progress reflected in the increasing number of clinical trials employing MNP-mediated MIH, particularly in GBM treatment.^2,238^ All of this knowledge has helped clinically translate at least some of these materials (iron oxide based) in reaching patients. On the other hand, this well-established field of research has pushed forward the magnetic material development in which great efforts have been dedicated to improving the properties of the MNPs in terms of crystallinity, composition, size control, shape definition, magnetism and heating ability.^6,13^ The discussion in this review has shed light on new avenues for the future, notably in areas of unmet medical need. These span direct disease intervention, diagnostic applications and bioengineering. In each, the biological landscape is unique, requiring careful consideration of safety and efficacy. Across the biomedical studies surveyed in this review, a clear preference for iron oxide MNP compositions was noted, particularly magnetite (Fe_3_O_4_). This may be justified given their long-standing and well-documented record of safety in biomedical applications, including their widespread clinical use as contrast agents and drug carriers. Some of the earlier stage papers used commercially available formulations, which further reflects the translational appeal.^27,38^ However, this also highlights a key opportunity, as there is substantial scope for performance gains through more tailored MNP design. In addition to this, a number of studies incorporated mixed ferrite compositions, which often provide enhanced magnetic and thermal properties. However, the incorporation of additional metal ions, such as Co, Mn, Ni, or Zn, strictly depending on the dose may alter biosafety. Use of ferrite compositions therefore should be accompanied by stringent *in vitro* and *in vivo* assessment. It's clear from the studies evaluated that these compositions can be used to great effect, such as in the endometriosis lesion removal,^31^ however safety and efficacy should be considered on a composition-by-composition basis and evaluated accordingly. The safety of mixed ferrite nanoparticles is partly supported by the body's natural metal homeostasis mechanisms. Following cellular uptake, ferrite nanoparticles gradually degrade in lysosomes, releasing iron and other metal ions such as zinc, manganese, or copper. These ions can enter physiological metabolic pathways, where they are regulated through storage, transport, and recycling processes involving proteins such as ferritin (for Fe and Co), transferrin, and metallothionein (for Zn and Cu).^239–241^ As a result, moderate ion release can be efficiently managed by the body. However, the long-term biocompatibility of mixed ferrites remains dependent on their degradation kinetics often related to the surface coating, dose, biodistribution, and composition. Indeed, excessive metal ion release may lead to oxidative stress, inflammation, or organ-specific toxicity, predominantly for liver, spleen, and other reticuloendothelial organs which play a central role in metal recycling and clearance.

The development of tailored MNPs offers a clear route to enhance specificity, therapeutic efficacy, and biodistribution. Studies could opt for non-spherical shapes (*i.e.*, hexagonal shapes, or nanodisk, or Rubik-cube or nanoflowers)^13^ to point to benchmark magneto heating performance ensuring the use of a minimal MNP dose. Such optimization is essential to comply with biological safety thresholds of the AMF parameters, to apply frequency and amplitude field conditions that maximize the magnetic heat losses while avoiding the induction of eddy currents which can cause non-specific heating of healthy tissues and physiological discomfort.^13,15,242,243^ By maximizing the SAR through MNPs parameters (anisotropic shapes, size distribution, size and composition)^31,244^ it is possible to achieve effective magneto hyperthermia at the lowest dose while remaining well within these clinical safety parameters. Moreover, the use of non-spherical shape nanoparticles would offer additional benefits such as changing the clearance time. Some studies indeed are also suggesting a different kinetic of degradation when moving from spherical to edge-like MNPs such as nanocubes, which may affect the body clearance.^245^

Additionally, including targeting ligands would redirect and enhance the MI effect toward the site of action,^246,247^ or to choose stimuli-responsive polymer for MNPs functionalization would enable the controlled release of therapeutics towards their target, to progress the various applications. A good example of this is the described Alzheimer's study, which utilized commercially available iron oxides.^27^ Here we have the advantage of decades of GBM research, giving us AMF applicators and parameters that have been carefully adapted for diseases of the brain. We also have access to MNP formulations functionalized with transferrin, lactoferrin, aptamers, peptides, or antibodies, which can enhance circulation time and promote receptor-mediated transcytosis to cross the blood-brain barrier allowing for safe and effective delivery.^248,249^

Alzheimer's disease and other neurodegenerative disorders are characterized by pathological hallmarks such as amyloid-β (Aβ) plaques, tau aggregates, and chronic neuroinflammation, which represent clinically relevant targets for both diagnosis and therapy. An emerging strategy is to exploit immune cells as biological “Trojan horses” for NP delivery. Rather than administering NPs directly to cross the blood-brain barrier, still a major challenge in nanomedicine, NPs can be loaded into immune cells, such as monocytes or macrophages, that naturally home to sites of neuroinflammation and are capable of passing through the BBB under pathological conditions. Once at the disease site, the NP cargo would enable targeted drug delivery, molecular imaging, or remote MI actuation. In support of this strategy, several studies have demonstrated the ability of peripheral immune cells to infiltrate neuroinflammatory regions in neurodegenerative diseases, supporting their potential use as carriers for NP-based therapies. Naturally, complex targeting specific to the application would be required, and in Alzheimer's may benefit from the notable progress in immunotherapy approaches in recent years.^250^

That said, examples of more complex, multifunctional MNPs are already beginning to emerge, even amongst these early use cases. Among the most compelling demonstrations is the endometriosis study highlighted in this review, in which bespoke MNPs were synthesized in-house and functionalized with a targeting moiety, enabling selective tissue accumulation and *in vivo* therapeutic efficacy.^31^ This work exemplifies the potential of moving beyond standard compositions toward tailored MNP formulations. Additionally, their use of mixed ferrite-based compositions is of note, as these materials have been shown to enhance heating performance, often outperforming their iron oxide counterparts, while maintaining favourable biosafety and stability profiles.

Looking across all the applications, it's worth emphasizing the potential for theranostic approaches, in which imaging and therapy are combined within a formulation, made possible by the careful engineering of their surface properties. This capability could be transformative in conditions such as endometriosis, where diagnosis currently relies on invasive surgical procedures. The prospect of using MNPs for both non-invasive imaging and localized therapeutic intervention represents a major shift with substantial clinical implications. Indeed, in addition to the widespread use of MNPs as contrast agents for MRI-based biodistribution studies and diagnostic imaging, their application as tracers in magnetic particle imaging (MPI) is also gaining increasing attention. MPI is an emerging imaging modality that, besides providing quantitative and highly sensitive detection of MNPs, offers real-time tracking without tissue background signals and without the use of ionizing radiation.^6^ These unique features make MPI particularly attractive to support the development of cell-mediated therapeutic and diagnostic strategies for neurodegenerative diseases, as above mentioned.^251,252^ Moreover, although human MPI scanners are still under development, the potential of MNPs in combination with MPI and localized MI has already been demonstrated in preclinical tumor models.^253^ This study highlights the feasibility of integrating therapeutic magnetic actuation with real-time imaging and tracking. Given the versatility of this approach, it is reasonable to envision its extension to other pathologies, including neurodegenerative and neurological disorders, where image-guided and localized therapeutic interventions could provide significant clinical benefits. This creates a feeling of near-term translational opportunities. With many of the core materials already validated for biomedical use, the field is poised to capitalize on a series of “quick wins,” whereby incremental advances in MNP design and targeting can rapidly yield meaningful improvements in targeted diagnostic and therapeutic outcomes. The use of organ-on-chip models can facilitate further optimization and assessment of new MNPs for specific applications.^38^ This is in particular due to their ability to mimic the complexity of the tumour microenvironment, allowing for inclusion of multiple cell types.

Among those explored, organ devitrification is one of the most promising biomedical engineering applications of MIH, with strong evidence across multiple organ systems from hearts to stem cells, as well as applications in fertility preservation.^63–70,84^ As in other biomedical uses, most studies we reviewed rely on iron oxide compositions because of their established safety, but in this setting optimization of MNP dispersion is far more important than targeting, since the goal is controlled and uniform heating of the entire tissue rather than a specific region. This makes future development likely to focus on improving distribution and stability to optimise thermal homogeneity during rewarming, particularly as this technology has the potential to be combined with emerging strategies to expand the donor pool such as rejuvenation of organs from older donors.^80^

Whenever feasible, particularly in applications requiring strict compositional safety, a spike-like temperature increase can be achieved by leveraging the dual functionality of Fe_3_O_4_ MNPs, which absorb both AMFs and near IR radiation. The combined magnetic and photothermal contributions can synergistically enhance overall heating efficiency and temporal control.^254^

In molecular diagnostics, MIH-driven PCR and biosensing both offer promising yet different routes toward next generation diagnostic platforms, with sensing in particular aligning closely with current trends in multiplexed testing.^255^ The work of Sanz de Diego *et al.* demonstrating protein identification provides a critical proof of concept, especially given the importance of proteins as clinical biomarkers.^115^ This opens the door to linking MNP sensing with other emerging NP based diagnostic strategies, including those that exploit the formation of a protein corona at NP surfaces.^256^ In contrast to therapeutic and warming applications, materials development in this space is guided mainly by binding specificity and surface chemistry rather than by magnetic heating performance and would form the basis of further development. On the other hand, nano-PCR is supported by a more extensive literature base, with MNP development focused primarily on maximising heating efficiency to support rapid and precise thermal cycling. The main goal is the development of compact and portable PCR systems. From a development perspective, biological tissue constraints are removed but strict control over enzyme compatible temperatures remains essential. While this application may not offer the same level of disruption as perhaps some therapeutic or regenerative uses, it is still a technically compelling direction, particularly as it allows exploration of a much wider range of MNP compositions since biocompatibility is not a limiting factor.

### 5.2 Perspectives for non-biomedical applications

Much like in biomedicine, the operational landscape of MIH in non-biomedical contexts is remarkably diverse, encompassing mild thermal processes like food pasteurization^149,159^ to the high-temperature transformations necessary for catalyst activation and NP synthesis.^129^ While toxicity, biocompatibility, and biodegradability of MNPs are crucial for medical-related applications of MIH, they are less significant outside of the biomedical field. In industrial or technical applications, any deficiency in particle efficiency can be easily compensated by increasing the field intensity or frequency of the time varying field, as there is no risk of working above well-defined threshold AMF, except concerns related to the energy-effective costs. Therefore, the development for still efficient MIH materials is still valid with the different aim to obtain the maximum magneto-energy conversion to the minimal energy cost.

The high-temperature applications (usually above 250 °C)^216^ like thermal catalysis clearly embrace high frequencies and fields without significant constraints for poor performing MNPs. However, the choice to work with metal MNPs (such as metal Fe), rather than spinel metal iron oxide structures, is associated with their high magneto heating performance and smaller sizes (superparamagnetic NPs of metal Fe have a diameter around 7 nm), thus providing a higher surface to volume ratio useful in catalysis to have more efficient conversion. The high SAR value (2000 W g^−1^) to reach from 25 °C to 350 °C in 30 s reported for CO_2_ hydrogenation exemplifies the capability of magnetic catalysts,^167^ often based on FeC MNPs, to function under strong hysteresis-driven heating. However, in this case, the need to preserve such performance for multiple catalytic runs, requires the use of shell, such as carbide in the case of FeC. This delays the exposure of iron metal surface to air, and thus its oxidation to iron oxide, with loss of MI heat performance, and at the same time prolonging the life of the catalyst. In addition, for the synthesis, high temperature/time profile (up to 200 °C within few minutes) are required to leverage nanoscale heat localization and facilitate nucleation, crystallization, or growth. For instance, synthesis of semiconductor dots, such as CDs at different emission colors, was performed by MIH reaching 230 °C within 3 min^−1^ while catalytic peroxide oxidation^129^ in wastewater treatment typically employs high temperatures above 250 °C. The temperature of magnetically susceptible hetero-Fenton catalyst (CoFe_2_O_4_) reached 400 °C to increase caffeine oxidation yield.^201^ These conditions emphasize MI as a thermal driving force capable of replacing conventional furnace treatments providing nanoscale localization and rapid cycling heat-up curves for multiple times.

Conversely, fields like food pasteurization and soil remediation illustrate how MIH can be precisely adjusted for low-heating applications (usually less than 100 °C). Food pasteurization operates at just 68 °C but it requires magnetic materials with exceptionally high SAR values (3100 W g^−1^) to reach desired temperature in a very short time interval (*i.e.* 60 s in this cited example). This approach reflects a strategy focused on rapid heating to low temperatures, which preserves food quality while ensuring microbial inactivation.^149^ Much less demanding is the change in temperature required for fluid viscosity modulation which needs generating a SAR of 77 W g^−1^ needed to reach temperature around 60 °C to induce rheological changes.^154^ Similarly for soil remediation, the temperature of soil sample with Fe(0) particles rapidly, but definitely less demanding than previous example was raised to 60 °C within 20 min leading to removal of the residual fraction of m-xylene in soil.^229^ These low-temperature applications highlight the selective, controllable, and non-invasive heating capabilities of MIH that are unattainable through bulk heating methods.

Drug amorphization, water electrolysis and CO_2_ desorption operate within moderate thermal windows (100–250 °C). The temperature for *in situ* amorphization of crystalline drugs needs to reach to 165.2 °C after 15 min.^148^ Water electrolysis was fulfilled using FeC@Ni generating high SAR value (2000 W g^−1^) to reach temperature of 200 °C.^199^ In CO_2_ capture and release, a SAR value of 150 W g_Fe_^−1^ was reported for the magnetic composite (Fe_3_O_4_ NPs embedded in a 13X Zeolite) when exposed to the AMF to reach 250 °C showing a significant improvement in the regeneration of the adsorbent material by controlling the energy required for gas release.^207^

The exhaustion or reduction of the heat generation ability of MNPs in catalysis, often referred to as a decrease in the SAR, is a primary limitation in long-term magnetically induced catalytic processes. This degradation is mainly caused by structural changes in the NPs due to high-temperature sintering, chemical transformation (oxidation), or loss of mobility/heat efficiency in the surrounding matrix. For instance, in catalytic reactions the magnetic Iron metal core can be coated by graphite,^185^ silica^187^ or carbide^186^ shells to avoid oxidation and loss of heat performance when exposed to multiple cycles of high temperature reactions.

Overall, the comparison highlights that MIH is not merely a single technique but a versatile thermal paradigm, capable of functioning across orders of magnitude of SAR and temperatures ranging from RT to hundreds of degrees celsius. This broad operational range positions MIH as a transformative technology for next-generation manufacturing and sustainable engineering. With this review a clear point to make is that beyond initial biomedical uses, MIH is emerging as a universal thermal platform with unparalleled selectivity, scalability, and energy efficiency across diverse scientific and industrial sectors.

### 5.3 Devices and applicators for MI

The use of MNPs in MIH is strictly related to the use of suitable equipment for the application of the AMF.^257^ As seen in the previous sections, different applications need different AMF conditions in terms not only of field strength and frequency applied but also types of coils in terms of size and geometries.^18^ In general, an AMF system consists of four main components: a power supply, a resonant circuit, an induction coil, and a cooling system. To achieve the high-frequency AMFs required for MIH, typically ranging from 50 kHz to 900 kHz, the system must operate at resonance to minimize impedance and maximize the current flowing through the coil.^258,259^ The most important part of the system is represented by the applicator. Its design is crucial as it dictates the spatial distribution and homogeneity of the MF while also complying with the need for specific experiments in terms of free space, accessibility and handling. The most common coil configurations are solenoidal coils, Helmholtz coils and pancake coils.^260–262^ Solenoidal coils are the most frequent choice for *in vitro* and small animal *in vivo* applications. This configuration offers high field homogeneity within the central volume at the cost of limited accessibility. Helmholtz coils consist of two parallel coaxial coils and provide an even larger uniform field region and more accessibility. With respect to limitations, they often require higher power to reach the same field intensities as solenoids. Finally, pancake coils are a flat coil configuration, frequently used for localized treatments or clinical applications where the target is placed on top of the coil, allowing for high intensity in small areas. A significant challenge in designing these instruments is the management of parasitic heating. Due to the Joule effect, the high currents circulating in the copper coils generate substantial heat. Without an efficient water-cooling system, this thermal energy could be transferred to the sample or the patient, leading to artifacts in the SAR measurements or unintended tissue damage. Furthermore, the use of Litz wires or hollow copper tubing is often necessary to mitigate the skin effect, which increases the resistance of the conductors at high frequencies.^263^ Hence the importance of knowing precisely the configuration and parameters of the systems used in this research community.

A systematic analysis of the literature conducted in this review reveals a concerning lack of experimental detail and reporting standards regarding the instrumentation used. Often, critical parameters, such as the field parameters such as *H* or *f* conditions and, in some cases, even the type of coil or device employed, are often omitted. To highlight this issue, we have included, as an Appendix, two tables summarizing all the experimental parameters reported in each of the original studies analyzed. From our analysis of the original papers using MI (149 publications in total), half of the surveyed studies do not provide comprehensive details about the instrument or its physical characteristics. Of those that do provide information, a significant portion of the studies (22%) employ commercial induction heating systems designed for general MI, which often lack integrated cooling or precise field control. Only a small portion (14%) use devices (*e.g.*, MagneTherm – NanoTherics, DM100_nb nanoScale) designed for MIH. The remaining studies (14%) rely on homemade setups, which, while tailored to specific needs, are often difficult to replicate elsewhere. This heterogeneity and lack of technical parameters make it difficult to compare results, especially when the geometry of the coil, the AMF parameters, or the cooling efficiency are not fully disclosed, calculating the SAR or heating profiles becomes prone to significant errors. This topic is already well known to the scientific community active in this field and for an in depth analysis, see some of these suggested works.^264,265^ To bridge this gap, and enable for instance, to make systematic comparisons in terms of energy/cost benefits, as a suggestion to the community, it is essential for future studies to move towards more rigorous instrumental data reporting, ensuring that every MIH experiment is accompanied by a detailed description of the AMF applicator and its operating conditions.

Besides the AMF systems for laboratory use, there is the need to develop instrumentation for clinical applications. The main challenge is the design of an applicator large enough to accomodate a human body.^266,267^ This poses fundamental challenges to control field strength and homogeneity and requires a huge amount of energy. With this goal set, a company and a European consortium are pioneering in this field. Nanotherm Therapeutics (Wilmington, USA) and is developing a closed-loop applicator.^268^ Contrastingly, NoCanTher multi-center Consortium has developed an open loop MF generator (RCL NTT Generator) which is more usable and versatile.^269^ These devices in combination with MNPs are currently in clinical trials for cancer treatment. They represent the next frontier of the AMF technology for humans, and at the same time, they give a clear view of a possible direction of development of MI technology.

## 6. Conclusion and final remarks

This review provides a comprehensive overview of current and emerging directions in MI research, covering a broad range of topics, including areas addressed by only a limited number of studies. We highlight the significant opportunity to leverage established knowledge in MNP design and MI applicator development to accelerate progress in these new areas beyond oncology. To visually summarize the landscape of the developed applications so far, we present a treemap visualization with a clear overview of the number of primary research papers across applications (Fig. 15).

**Fig. 15:**
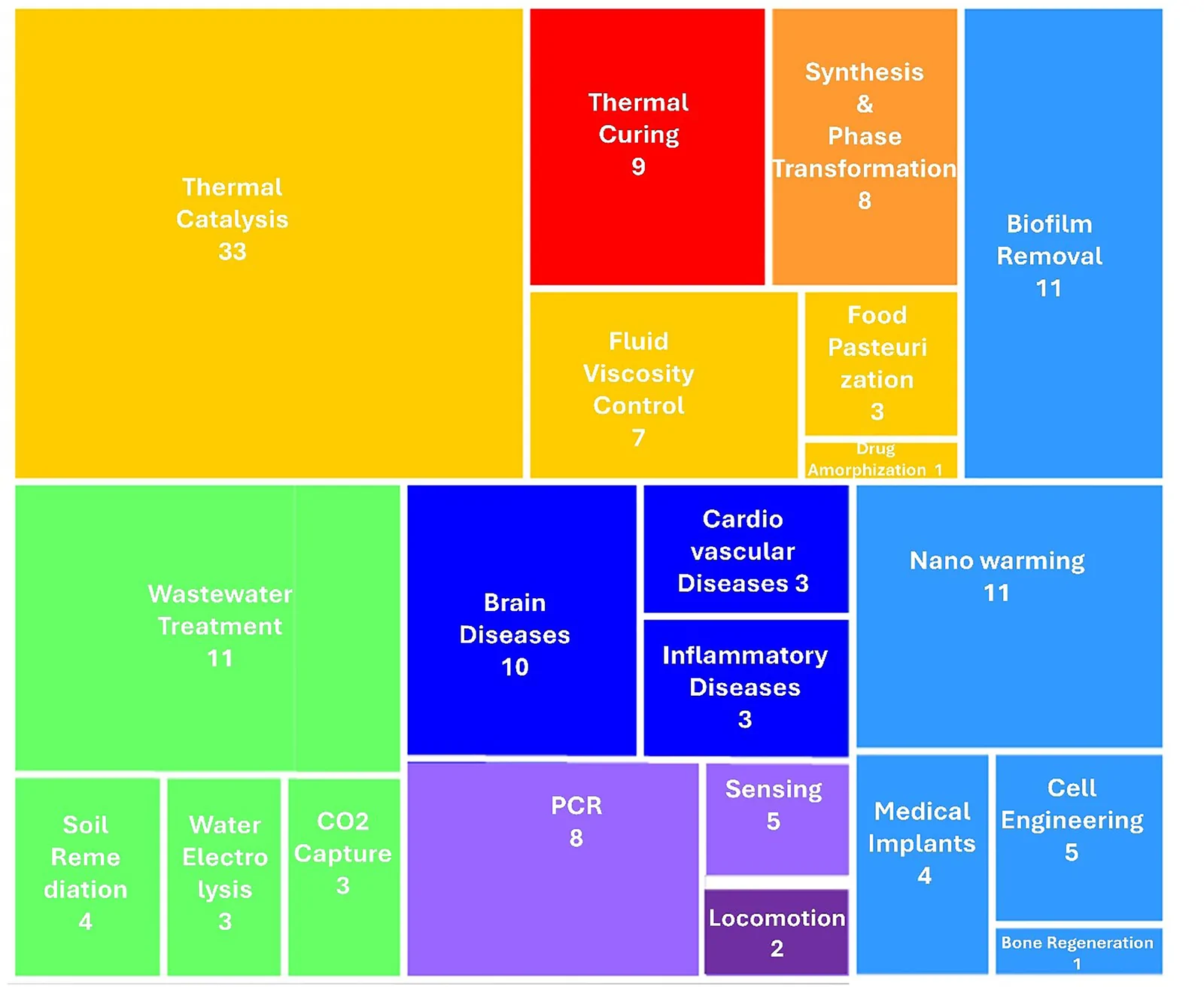
Tree map visualization of MI applications in various research fields. For each application, presented in different colour codes, the box name represents the specific area of development and number refers to the number of original manuscripts published in literature on the specific topic (*i.e.* 33 means the number of original papers published in thermal catalysis, while only 4 papers were published in medical implants, *etc.*), giving a visual representation of the directions of current research.

Besides thermal catalysis, which dominates the research landscape with as many as 33 original papers using MIH, nano-warming (11 papers), biofilm treatment (11 papers), waste treatment (11 papers), and brain diseases (10 papers) also represent major areas of investigation. Furthermore, a multitude of niche applications are emerging, demonstrating how MIH is permeating different research fields from various angles. Notably, within unmet medical needs, brain-related applications dominate, likely reflecting the potential to repurpose applicators and MNPs originally developed for GBM. Biofilm and nanowarming studies are central to bioengineering, with biofilm treatment in particular showing broad applicability throughout the whole body, for example in dental applications and surgical implants. Volumetric heating mediated by MNPs offers a promising approach for rewarming cryopreserved organs (with the highest mean heat rate reported of 321 °C min^−1^ ^63^ when using only AMF but up to 93 °C s^−1^ when used MIH in combination with laser),^63^ enabling the rapid, spatially and uniform temperature increase required to limit ice crystallization and reduce thermal gradients and associated tissue mechanical stresses.

In industrial applications, we see a predominant focus on thermal catalysis. Sustainable futures mainly addresses wastewater treatment, which has global relevance. Overall, this targeted analysis not only maps the current research activity but also highlights key areas where future investigations and advancements are likely to unfold.

Looking forwards, there are two key aspects require attention. Firstly, for medical needs, the clinical translation and commercialization of materials and devices remain hindered by complex regulatory hurdles. A primary bottleneck lies in defining the appropriate certification pathway, as MNPs can theoretically fall under diverse regulatory frameworks, ranging from medical devices and contrast agents to pharmaceutical drugs or even dietary supplements. Moreover, novel applications frequently blur these traditional boundaries. To mitigate these challenges, regulatory agencies such as the FDA (https://www.fda.gov/) and EMA (https://www.ema.europa.eu/en/homepage) are taking proactive steps. By fostering early dialogue and offering tailored advice, these agencies aim to clarify classification ambiguities and streamline the certification process for innovative MNPs-based technologies. The successful approval of pioneering products, such as Magtrace® (Endomag), alongside others currently in the certification pipeline, provides a crucial regulatory precedent. By establishing clear reference frameworks, these early successes are paving the way for future MNPs innovations, potentially accelerating their path to the clinical use.

On the other hand, the omission in reporting of key physical parameters hinders the accurate calculation of heat adsorption rate indicators (such as SAR) and prevents precise comparisons between different studies. Establishing rigorous reporting protocols is essential to ensure experimental reproducibility and data cross-validation among the scientific community and to prevent delays to progress and translation.

This second key aspect affects mostly the industrial and manufacturing sectors. The limited availability of SAR and ILP data in the literature (with ILP defined as ILP = SAR/*H*^2^*f*, and commonly used to compare the heating efficiency of superparamagnetic NPs independently of the magnetic field conditions), also makes challenging the systematic assessment of the MIH thermal demand for different applications, and consequently of their energy and cost efficiency evaluation.

We sought to examine an alternative parameter, that is the temperature achieved during a specific process. Interestingly, temperature data are reported more consistently across the literature than SAR or ILP values. Therefore, examining the temperatures, when explicitly stated, and comparing them to the nominal target temperature required for that specific application or task provides valuable insight into the actual thermal demands of each process (Fig. 16). To facilitate the data reading, we have grouped these values by type of applications (Fig. 16).

**Fig. 16:**
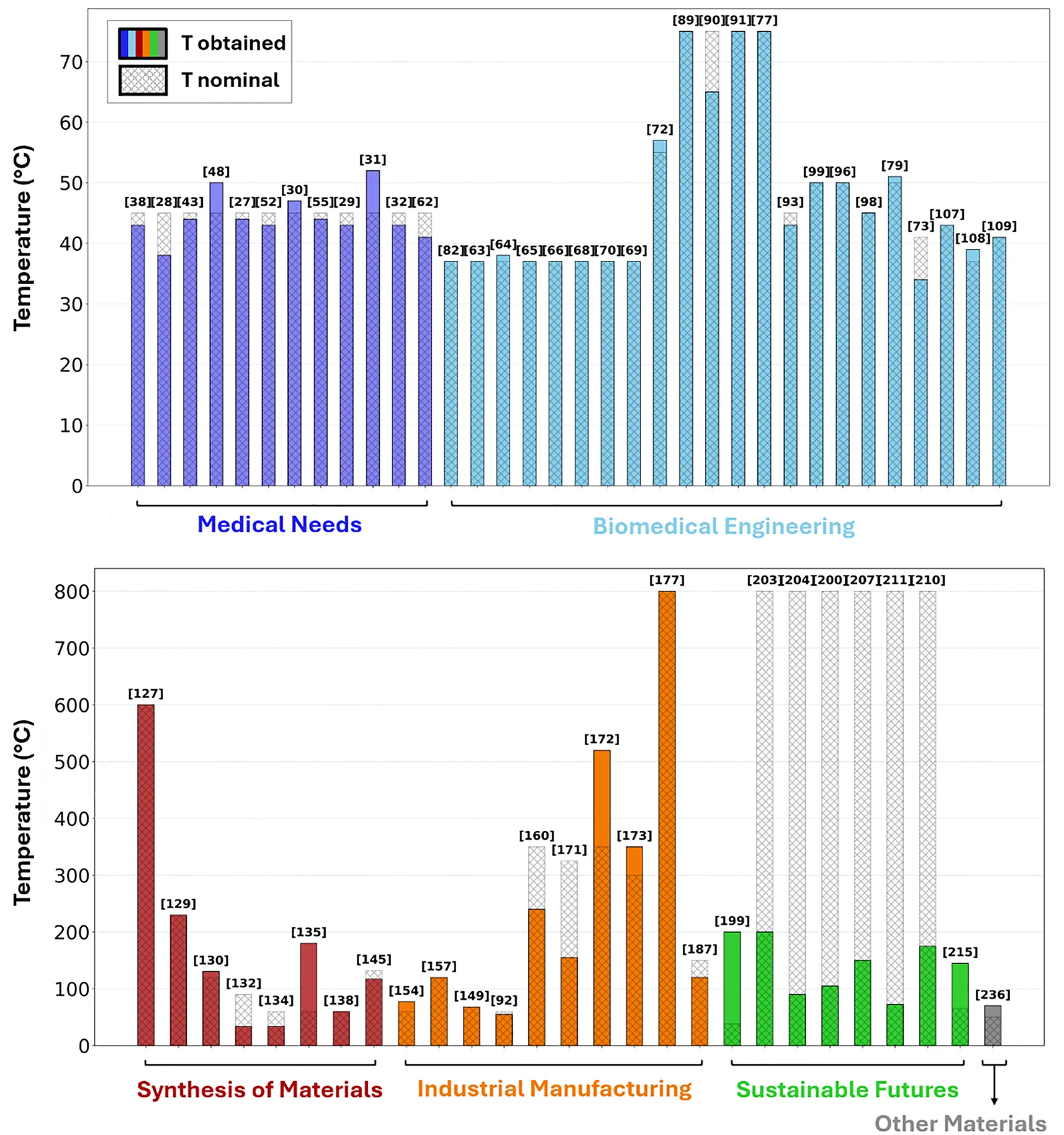
Summary of the available nominal temperatures, shown as the textured grid, *versus* the MIH temperatures achieved, shown by the solid colour bar, based on temperature data reported in original published works. To support comparative analysis, the data were grouped by area of application, while the number indicate the corresponding reference assigned in this review.

In biomedical applications, the target temperature as well as the variation in achieved temperatures is minimal (only few degrees discrepancy), as these systems must operate strictly within safe temperature ranges for human use. With the notable exception in one particular biofilm bioengineering application, where higher temperatures are targeted to disrupt biofilms on the surface of surgical implants.^91^ It is evident that, in some applications, further improvement is still needed to reach the nominal temperature levels. This gap could potentially be addressed through the development of bespoke MNPs, as discussed previously.

Furthermore, it is noteworthy that for many industrial manufacturing applications (such as viscosity control,^147^ thermal catalysis,^160^ phase transformation of materials^132^) the temperatures required to achieve the objectives of these studies are significantly lower than the standard temperatures used with conventional heating methods (Fig. 16). In fact, these transformations are made possible by a localised heat at the surface of the MNPs under an AMF, which leads to conditions involving temperatures well below the macroscopic ones.

In environmentally sustainable fields, a large discrepancy is observed between nominal and achieved temperatures. The use of localized heat foci may account for the ability to perform CO_2_ capture/release or waste treatment at temperatures significantly lower than those required with external heat sources, thereby improving the energy efficiency of gas separation processes, particularly during the regeneration phase of adsorbent materials.

This evidence, together with numerous indirect observations in biological fields where significant indirect biological phenomena have been reported despite bulk temperatures remaining below the nominal threshold required for the process (*e.g.*, thermal activation of ion channels, enhanced cellular uptake, and modulation of cellular pathways),^73–75,105–108^ highlight one of the most intriguing and unresolved questions in the field: how can the effective temperature at the MNP surface be accurately measured?

This challenge goes beyond MIH and is common to many nanothermal technologies such as near- infrared (NIR), photo-irradiation, microwave heating *etc.* Conventional thermometry methods generally provide only the average temperature of the surrounding medium, whereas the relevant biological or chemical events may be governed by highly localized thermal gradients generated at the nanoparticle interface. As a result, the existence, magnitude, and functional relevance of nanoscale overheating remain the subject of ongoing debate.

Few strategies have been proposed to address this issue. Among the most promising are luminescent nanothermometers based on lanthanide-doped upconverting nanoparticles (UCNPs), which exploit ratiometric temperature-dependent emission ratios or lifetimes to provide local thermal information with high spatial resolution.^270,271^ Also, more recently, silver sulfide NP-based materials have been mentioned.^272,273^ Other approaches rely on fluorescent probes tethered to the nanoparticle surface through thermolabile linkers.^270,274^ Additionally, the underlying physics of MPI has been proposed as a potential strategy for real-time and non-invasive magnetic nanothermometry, although its ability to directly probe the local temperature at the nanoparticle surface remains to be demonstrated.^275^

Despite experimental studies, including Riedinger *et al.*,^274^ which reported a molecular thermometer based on azo bond cleavage and inferred local temperature increases up to ∼45 °C near the iron oxide surface and the temperature profile appeared to decay exponentially, their interpretation remains controversial. Theoretical analyses based on continuum heat-transfer models have questioned whether temperature differences of such magnitude can be sustained in aqueous environments.^276^ Conversely, it has been argued that the applicability of classical heat diffusion theory at molecular and subnanometer length scales may be limited, particularly in the presence of interfacial thermal resistance and non-equilibrium energy dissipation processes.^274^ Consequently, the extent to which nanoparticle-mediated biological and chemical effects originate from genuine local overheating, nanoscale thermal gradients, or alternative interfacial phenomena remains an open question that warrants further investigation.

Overall, the analysis of temperature comparison across various fields, highlights how MIH is not merely a single technique but a versatile thermal tool, capable of functioning across orders of magnitude of SAR and temperatures ranging from RT to hundreds of degrees celsius. This broad operational temperature range positions MIH as a transformative technology for next-generation manufacturing and sustainable engineering. With this review a clear point to make is that beyond initial biomedical uses MIH is evolving into a universal thermal platform with unparalleled selectivity, scalability, and energy efficiency across diverse scientific and industrial sectors.

As a further remark, although most MIH studies rely on MNPs and heat generation through dynamic hysteresis losses, the efficiency of these systems remains highly dependent on particle size, composition, intrinsic magnetic properties, and, importantly, the surrounding microenvironment. Immobilization or aggregation, as frequently occurs following substrate immobilization or cellular internalization, often leads to a substantial reduction in SAR, highlighting the sensitivity of relaxation-based heating mechanisms to local constraints. By contrast, a smaller but growing body of work has explored heating based on Joule losses generated by eddy current heating with liquid metals,^277,278^ such as Fields metal^72^ and Ga-based liquid metal^87^ and example of applications in implant removal, but also locomotion based on levitation or more recently onco-thermal therapy.^236,279^

While eddy current losses are fundamentally negligible in discrete nanometre-scale particles, as the dissipated volume power scales with the second power of the particle radius (*R*^2^) at low frequencies, they become increasingly significant as particle dimensions approach the micrometre scale.^25^ This behaviour is particularly evident in liquid metal microspheres (LMMSs) with diameters exceeding 100 µm, which exhibit strong size-dependent thermogenesis. Unlike MNPs, eddy-current-mediated heating is largely insensitive to the viscous and spatial constraints, providing a robust and predictable source of heat generation even under conditions of particle immobilization or intracellular confinement. Together these classes of materials and types of heating approaches account for the majority of reports but they may not exhaust the possible physical pathways for magnetically induced heating. Depending on the development of the material, other under-explored routes such as magneto-mechanical heating (*i.e.* magnetostriction) could be used.^280–282^ This heating mechanism arises from magnetic torque and/or force on a magnetically anisotropic object, leading to conversion of field energy into mechanical work and subsequent dissipation through viscous drag and internal friction. This opens further doors for development using other materials and mechanisms to optimize transfer to clinical applications.

We conclude that the MI field has vast untapped avenues outside of oncology and would benefit from further exploration in so many different branches.

## Conflicts of interest

There are no conflicts to declare.

## Abbreviations

AMF: Alternating magnetic field
CCS: Carbon capture and storage
CDs: Carbon dots
EMF: Electromagnetic field
GHG: Greenhouse gas
GBM: Glioblastoma multiforme
HSPs: Heat shock proteins
MF: Magnetic field
MI: Magnetic induction
MIH: Magnetic induction heating
MIP: Molecularly imprinted polymer
MISA: Magnetic induction swing adsorption
MNPs: Magnetic nanoparticles
MOF: Metal-organic framework
MSCs: Mesenchymal stem cells
NIR: Near-infrared
NPs: Nanoparticles
PCR: Polymerase chain reaction
PEMF: Pulsed electromagnetic field
SAR: Specific absorption rate
SPIONS: Super paramagnetic iron oxide nanoparticles
tPA: Tissue plasminogen activator
TRPV1: Transient receptor potential vanilloid 1
TSA: Temperature swing adsorption

## Supplementary Material

CS-055-D5CS01155H-s001

CS-055-D5CS01155H-s002

## Data Availability

No primary research results, software or code have been included, and no new data were generated or analysed as part of this review. Supplementary information (SI): includes comparative overview of the structural properties, magnetic induction heating parameters, and thermal performance of various magnetic nanomaterials exploited in MIH for ‘innovation across biomedicine’ (Table S1) and for the ‘synthesis of materials and industrial manufacturing’ (Table S2). See DOI: https://doi.org/10.1039/d5cs01155h.
