# Supplementary material for: Magnetic induction heating: where will the field go next?
Source: Chem Soc Rev. 2026 Oct 14. Online ahead of print. doi: 10.1039/d5cs01155h (PMC13666889; doi:10.1039/d5cs01155h)
Supplement: Supplementary file 1 [file CS-OLF-D5CS01155H-s001.pdf]

Supplementary Information (SI) for Chemical Society Reviews.
This journal is © The Royal Society of Chemistry

BIOMEDICAL UNMET NEEDS

Composition	Size [nm]	SAR [W/g$_{Fe}$]	Temperature nominal [°C]	Temperature obtained [°C]	Frequency [kHz]	filed [kA/m]	H×f [A/m*s]	DOI	Ref num
Fe_3O_4	100	115.4 ± 6.0	45	43	305	23.9	7.29*10^9	https://doi.org/10.31744/einstein_journal/2020AO495	38
Fe_3O_4	180	1300 ± 0.1	45	38	306	23.9	7.31*10^9	https://doi.org/10.1111/jth.15846	28
γ-Fe_2O_3	25 ± 3	\	45	44	\	\	\	https://doi.org/10.1038/s41598-023-31979-w	43
γ-Fe_2O_3	25 ± 3	139	\	\	595	5.6	3.33*10^9	https://doi.org/10.1016/j.brs.2024.08.011	44
Fe_3O_4	13 ± 5	\	\	\	150	7.6	1.1*10^9	https://doi.org/10.1016/j.jconrel.2015.02.027	45
Fe_3O_4	99.8 ± 21.5	\	\	\	\	\	\	https://doi.org/10.1088/1361-6528/abb392	47
Fe_3O_4	205	\	45	50	100	30	3*10^9	https://doi.org/10.1002/smll.202400408	48
Fe_3O_4	20	\	45	44	375	68	25.5*10^9	https://doi.org/10.1016/j.nano.2021.102397	27
Fe_3O_4	50	\	45	43	375	68	25.5*10^9	https://doi.org/10.1038/s41598-025-10441-z	52
\	20	\	\	\	375	60	25.5*10^9	https://doi.org/10.1002/alz.088468	54
\	\	\	45	47	\	\	\	https://doi.org/10.1186/s12951-024-02716-w	30
Fe_3O_4	69.2 ± 5.4	\	45	44	375	26	9.7*10^9	https://doi.org/10.1021/acsnano.4c04331	55
Fe_3S_4	13	\	45	43	595	14.5	8.62*10^9	https://doi.org/10.1016/j.apmt.2019.100457	29
Fe_3S_4	18.9 ± 1.8	8057	45	52	420	26.9	11.29*10^9	https://doi.org/10.1002/smll.202107808	31
$Mn_{0.5}Zn_{0.5}Fe_2O_4$	5.5	256.4	45	43	378	11.1	4.19*10^9	https://doi.org/10.1016/j.biomaterials.2010.09.016	32
$Mn_{0.5}Zn_{0.5}Fe_2O_4$	7.20 ± 0.85	\	45	41	140	11.1	1.55*10^9	https://doi.org/10.1155/2015/142387	62

BIO ENGEENERING

Composition	Size [nm]	SAR [W/g$_{Fe}$]	Temperature nominal [°C]	Temperature obtained [°C]	Frequency [kHz]	filed [kA/m]	H×f [A/m*s]	DOI	Ref num
Fe_3O_4	10.02 ± 1.56	\	37	37	375	\	\	https://doi.org/10.1016/j.actbio.2016.01.026	82
Fe_3O_4	10	\	\	\	175	60	10.5*10^9	https://doi.org/10.1126/scitranslmed.aah4586	83
IONPs	12.6	\	37	37	278	42.5	11.81*10^9	https://doi.org/10.1126/sciadv.abe3005	63
Fe_3O_4	length of 530.59 ± 58.32 width of 45.84 ± 5.51	\	\	\	358	35	12.53*10^9	https://doi.org/10.1021/acs.nanolett.4c03081	84
Fe_3O_4	10	\	37	38	208	31.9	6.63*10^9	https://doi.org/10.1002/btm2.10416	64
IONPs	\	\	37	37	\	\	\	https://doi.org/10.1038/s42003-023-04577-9	65
IONPs	\	\	37	37	185	63	11.6*10^9	https://doi.org/10.1007/s10439-022-03064-2	66
Fe_3O_4	10		37	37	180	63	10.5*10^9	https://doi.org/10.1038/s41467-023-38824-8	68
IONPs	\	\	\	\	\	\	\	https://doi.org/10.1002/advs.202101691	67
Fe_3O_4	\	\	37	37	\	\	\	https://doi.org/10.1038/s41467-022-34549-2	70
Fe_3O_4	\	\	37	37	400	26	10.4*10^9	https://doi.org/10.1016/j.heliyon.2023.e18828	69
\	\	\	\	70	\	\	\	https://doi.org/10.3892/etm.2013.1177	71
\	\	\	\	\	\	\	\	https://doi.org/10.1080/02656736.2021.1909758	85
LM	\	\	55	57	\	\	\	https://doi.org/10.1016/j.apmt.2022.101459	72
Fe_3O_4	\	\	\	60	60	\	\	https://doi.org/10.1016/j.apmt.2022.101495	88
Fe_3O_4	\	\	75	75	\	\	\	https://doi.org/10.1038/s41598-023-49660-7	89
\	\	\	75	65	\	8.7	\	https://doi.org/10.1038/s41522-021-00239-y	90
\	\	\	75	75	200	8.7	1.7*10^9	https://doi.org/10.1080/02656736.2022.2065038	91
\	\	\	75	75	213.8	8.75	1.9*10^9	https://doi.org/10.1016/j.jinf.2024.106271	77
Fe_3O_4	64	\	45	43	80	6.9	0.5*10^9	https://doi.org/10.1128/AEM.04103-15	93
Fe_3O_4	\	15	50	50	\	\	\	https://doi.org/10.1002/smll.202510260	99
Fe_3O_4-ZnO	800	\	\	55	250	34	\	https://doi.org/10.1016/j.powtec.2014.09.032	94

Fe_3O_4	74	\	\	45	\	\	\	https://doi.org/10.1021/acsami.1c24148	95
$MnFe_2O_4$	151	\	50	50	\	1.3	\	https://doi.org/10.1002/smll.202304836	96
Fe_3O_4	10.43 ± 0.2	\	\	\	\	\	0.5*10^9	https://doi.org/10.1007/s13346-024-01667-x	97
BMNPs	40 ± 20	120	45	45	140	22	3.1*10^9	https://doi.org/10.1016/j.ijbiomac.2021.08.110	98
\	\	\	\	\	\	\	\	https://doi.org/10.1016/j.joen.2011.10.019	100
Fe_3O_4	300	\	\	\	\	\	\	https://doi.org/10.1016/j.identj.2024.07.1219	78
BFTO	\	\	50	51	310	5.3	1.6*10^9	https://doi.org/10.1021/acsnano.4c13562	79
$MnFe_2O_4$	6	\	41	34	40000	\	\	https://doi.org/10.1038/nnano.2010.125	73
Fe_3O_4	22	660 ± 50	43	43	500	15	7.5*10^9	https://doi.org/10.1126/science.1261821	107
$CoFe_2O_4$	10	733.3 ± 2.8	37	39	412.5	37	15*10^9	https://doi.org/10.7554/eLife.27069	108
Fe_3O_4	13	\	\	87	445	16.9	7.52*10^9	https://doi.org/10.1039/D4NR01955E	74
$CoFe_2O_4$–$BaTiO_3$	99.4 ± 4.2	\	\	\	1.18	16	18.8*10^9	https://doi.org/10.1038/s41563-026-02625-3	75
Fe_3O_4	17	200	41	41	182	24	4.37*10^9	https://doi.org/10.1002/smtd.202502416Digital Object Identifier (DOI)	109
**MOLECULAR DIAGNOSTIC**									
**Composition**	**Size [nm]**	**SAR [W/g$_{Fe}$]**	**Temperature nominal [°C]**	**Temperature obtained [°C]**	**Frequency [kHz]**	**filed [kA/m]**	**H×f [A/m*s]**	**DOI**	**Ref num**
Fe_3O_4 - $CoFe_2O_4$	Mixed	\	\	\	100	24	4.77*10^9	https://doi.org/10.1039/D3NR06489A	116
Fe_3O_4	11.8	\	\	\	180	\	\	https://doi.org/10.3390/ s21010185	114
\	116.7 ± 47.1	\	\	\	\	\	\	https://doi.org/10.1002/cbic.202400251	117
Au-Fe_3O_4	\	\	\	\	\	\	\	https://doi.org/10.1002/smll.202104596	118
Fe_3O_4	13 ± 5	\	\	\	\	\	\	https://doi.org/10.1039/D3NR02593D	113
Fe_3O_4	10	\	\	\	\	\	\	https://doi.org/10.3390/app2020303	120
Fe_3O_4	10	\	\	\	\	\	\	https://doi.org/10.1016/j.cplett.2011.03.017	121
Fe_3O_4	10	\	\	\	\	\	\	https://doi.org/10.1016/j.cplett.2015.06.070	122
Fe_3O_4	30	81.4	\	\	\	3	\	https://doi.org/10.1088/0957-4484/21/16/165102	123
Fe_3O_4	11	\	\	\	\	\	\	https://doi.org/10.1038/am.2016.70	124
\	\	\	\	\	\	\	\	https://doi.org/10.1109/BioCAS.2012.6418414	125
Fe_3O_4@Au	\	\	\	\	\	\	\	https://doi.org/10.1016/j.apmt.2021.101054	126
Fe_3O_4	\	\	\	\	\	\	\	https://doi.org/10.1016/j.colsurfb.2016.02.024	127

Table S1 : Comparative overview of the structural properties, magnetic induction heating parameters, and thermal performance of various magnetic nanomaterials and bulk structures reported in the literature for biological applications, as discussed in the chapter *Innovation Across Biomedicine*. The table uses color coding to distinguish the different application areas (e.g., dark blue for medical applications, light blue for biomedical engineering applications and pink for molecular diagnostic). The columns report the material composition, particle size, specific absorption rate (SAR), target and achieved temperatures for the specific process, operating frequency (f), magnetic field intensity (H), the H × f factor, and

the corresponding references. Empty fields indicate that the relevant data could not be retrieved from the original publication.
