# Supplementary material for: Magnetic induction heating: where will the field go next?
Source: Chem Soc Rev. 2026 Oct 14. Online ahead of print. doi: 10.1039/d5cs01155h (PMC13666889; doi:10.1039/d5cs01155h)
Supplement: Supplementary file 2 [file CS-OLF-D5CS01155H-s002.pdf]

Supplementary Information (SI) for Chemical Society Reviews.
This journal is © The Royal Society of Chemistry

Composition	Size [nm]	SAR [W/g_{Fe}]	Temperature nominal [°C]	Temperature obtained [°C]	Frequency [kHz]	filed [kA/m]	H×f [A/m*s]	DOI	ref
**SYNTHESIS OF MATERIALS**									
Iron	Bulk sheets	-	600	600	30	-	-	https://doi.org/10.34133/2022/9756983	127
copper	Bulk foils	-	\	1035	240	-	-	https://doi.org/10.1021/nn4031564	128
Fe_3O_4	20	-	230	230	450	-	-	https://doi.org/10.1002/anie.201914331	129
Fe_3O_4	24	-	120	130.5	-	22	-	https://doi.org/10.1021/acsaenm.4c00048	130
Fe_3O_4	21.5	-	\	96	-	31	-	https://doi.org/10.1021/acssuschemeng.9b02323	131
PEGylated $Fe_{1-x}O/Fe_{3-\delta}O_4$ core-shell nanocubes	17±2	106	90	34	302	20	6.07* 10^9	https://doi.org/10.1038/srep33295	132
SiO2-coated Fe3O4/Fe2O3	<100	-	\	200	25	-	-	https://doi.org/10.1002/anie.200801474	133
Fe_2O_3	10	-	60	34	342	7.2	2.5* 10^9	https://doi.org/10.1021/acsanm.7b00063	134
CoFe2O4@MnFe2O4	15	-	60	180	500	40	20* 10^9	https://doi.org/10.1021/acs.nanolett.1c00770	135
$CoFe_2O_4$	13.7± 1.2	60	60	60	100	40	4*10^9	https://doi.org/10.1021/acsapm.4c00271	138
Ni81Fe19	500	-	-	-	50Hz	<103.5	<0.005*10^9	https://doi.org/10.1038/s41467-022-34235-3	139
Teresa	15-21	-	-	238	930	30	27.9*10^9	https://doi.org/10.5281/zenodo.20704137	140
Fe_3O_4	900	36.78	-	-	142	-	-	https://doi.org/10.1016/j.matchemphys.2015.08.038	141
CrO_2	-	-	-	92-110	390	12	4.6*10^9	https://doi.org/10.1002/adma.200904138	143
Fe_2O_3	12	-	-	43	278	-	-	https://doi.org/10.1002/adma.200901940	144
Fe_2O_3	17	-	132	117	968	12.1	11.7*10^9	https://doi.org/10.1038/s41565-018-0327-4	145
$Mn_xZn_{1-x}Fe_2O_4$	12-15	-	-	150-220	475	4	1.8*10^9	https://doi.org/10.1557/jmr.2015.59	146
**INDUSTRIAL MANUFACTURING**									
**Composition**	**Size [nm]**	**SAR [W/g_{Fe}]**	**Temperature nominal [°C]**	**Temperature obtained [°C]**	**Frequency [kHz]**	**filed [kA/m]**	**H×f [A/m*s]**	**DOI**	**ref**
oleic acid-capped iron oxide NPs	8	55 W/g	-	-	430	16	6.8*10^9	https://doi.org/10.1021/acsanm.1c03061	147
oleic acid-capped iron oxide NPs	9	-	-	-	533	14	7.4*10^9	https://doi.org/10.1021/acsomega.3c02832	152
oleic acid-capped iron oxide NPs	9	-	-	-	780	28	21.8*10^9	https://doi.org/10.1016/j.fuel.2022.125810	153
$Zn_xFe_{3-x}O_4$ NPs	-	-	60	77	570	16	9.1*10^9	https://doi.org/10.1021/acs.jpcc.0c06843	154
OA-capped iron oxide nanocubes	25	-	120	120	274	30	8.2*10^9	https://doi.org/10.1002/adma.202100074	157
$Zn_{0.5}Fe_{2.5}O_4$ and $Mn_{0.5}Fe_{2.5}O_4$	12-20	-	-	165.2	588.5	11.1	6.53*10^9	https://doi.org/10.1021/acsami.2c03556	148
$Fe_3O_4@SiO_2$ core-shell	50 µm	3108.4 ± 30.6	68	68	400	-	-	https://doi.org/10.1016/j.jfoodeng.2020.110079	149
-	-	-	-	62.5	40	-	-	https://doi.org/10.3390/FOODS11233937.	159
PAA-coated Fe_3O_4	10	-	60	55	87	-	-	https://doi.org/10.1109/TMAG.2013.2247384	92
Iron oxide-Au	33 ± 8<br>24 ± 6		-	45	110	-	-	https://doi.org/10.1039/D0CY00439A	150
Fe@FeCo<br>Fe@Ru	12.2 ± 0.9<br>12.4 ± 0.6		350	240	54	39.9	2.15*10^9	https://doi.org/10.1021/acs.nanolett.5b00446	160
FeC NPs<br>FeC @ Ni		3200-2000	-	-	100	37.7	3.77*10^9	https://doi.org/10.1002/anie.201609477	167
Cobalt–Nickel Nanoparticles	35	-	-	-	69	25.5	1.76*10^9	https://doi.org/10.1002/anie.201804832	168
Fe_3O_4	80-120	-	-	250	205	19.1	3.91*10^9	https://doi.org/10.1021/acscatal.3c05989	169
$CoFe_2O_4$ silica coated	25-26	-	-	(Ni)137,(Re)87	-	-	-	https://doi.org/10.1039/D3TA03654E	170
FeCo@Ni<br>FeCo@Ni@C	13.8 ± 5.9	-	325	155	300	39	11.70*10^9	https://doi.org/10.1039/D0GC03495A	171

Fe30Ni70-Ni@SiRAlOx	17.3 ± 2.2	350	350	520	300	15	4.5*10^9	https://doi.org/10.1002/anie.201913865	172
$Fe_{2.2}C$-Ni@$SiRAlO_x$	14-15	2100	300	350	300	12	3.6*10^9	https://doi.org/10.1039/C9CY00437H	173
Co@$MgAl_2O_4$ NiCo@$MgAl_2O_4$	10-24	-	800	800	68	31.8	2.1*10^9	https://doi.org/10.1021/acs.iecr.7b02331	177
MAGSILICA@-Au	20	-	150	120	25	-	-	https://doi.org/10.1002/adsc.201400261	187
Oleic acid-capped iron oxide NPs	17-18	-	-	-	343	47.7	16.3*10^9	https://doi.org/10.1002/cssc.202002775	188

SUSTAINABLE FUTURES

Composition	Size [nm]	SAR [W/g_{Fe}]	Temperature nominal [°C]	Temperature obtained [°C]	Frequency [kHz]	filed [kA/m]	H×f [A/m*s]	DOI	ref
FeC-Ni core-shell	15	2000	38	200	300	38	11.40*10^9	https://doi.org/10.1038/s41560-018-0132-1	199
Iron-coated nickel foam	thin film	-	800	200	-	0.0017	-	https://iopscience.iop.org/article/10.1088/1361-6463/ad816d	203
NiFeN@NiFeOOH core–shell	55	-	800	90	-	-	-	https://doi.org/10.1021/acs.nanolett.2c03634	204
$MgFe_2O_4$	200 - 700	55.4	800	105	-	42	-	https://doi.org/10.1021/acs.chemmater.6b02409	200
Fe_3O_4	-	150	800	150	-	-	-	https://doi.org/10.1016/j.cej.2021.133380	207
Fe3O4	50	-	800	72.5	30	0.12	0.036*10^9	https://doi.org/10.1021/acs.energyfuels.0c02699	211
Fe3O4	28	-	800	175	190	7.9	1.50*10^9	https://doi.org/10.1016/j.matchemphys.2023.128525	210
sponge iron particle (Fe^s) coated by carbon layer	Thin layer	-	65	145	50	-	-	https://doi.org/10.1016/j.cherd.2016.08.020	215
Magnetite	12 ± 4	-	-	60	222	-	-	https://doi.org/10.3390/nano9111508	216
Magnetite/maghemite	21 ± 7	-	-	90	222	23.8	5.2*10^9	https://doi.org/10.1016/j.jelechem.2020.114773	217
$CoFe_2O_4$	12	-	-	400	100	-	-	https://doi.org/10.1016/j.chemosphere.2023.138364	200
Magnetite/maghemite assembled into the multicore particles	10 -45	-	-	90	200	17	3.4*10^9	https://doi.org/10.1016/j.jclepro.2021.127385	218
Single core Multicore	15 ± 3 60 ± 8	144-326	-	50	200	24	4.8*10^9	https://doi.org/10.3390/nano11041052	219
Magnetite/maghemite assembled into the multicore particles	8 and 33 ± 4	-	-	-	100	47.7	4.7*10^9	https://doi.org/10.1016/j.cej.2024.151725	220
Fe-CNT layer on PTFE	Thin layer	-	-	-	283	-	-	https://doi.org/10.1016/j.memsci.2019.117253	221
Dextran-coated Fe3O4	50	-	-	180	604	23.8	14.3*10^9	https://doi.org/10.1021/acsami.9b19641	222
steel slag composed mainly of Fe_3O_4 (31 wt%), Fe_2O_3 (22 wt%), CaO, MnO, and graphite	-	-	-	56	200	15	3*10^9	https://doi.org/10.1016/j.jhazmat.2023.131264	202
Iron oxide nanoparticles	<10	-	-	-	332	-	-	https://doi.org/10.1680/jgele.19.00098	227
ZVI powders nanoscale ZVI (NZVI) Fe^0 with surface Fe_3O_4 shells	-	-	-	123	150	-	-	https://doi.org/10.1021/acs.est.5b04485	228
millimetric ZVI and micrometric ZVI powders Fe_3O_4 and α-Fe_2O_3	-	-	-	60	100	36	3.6*10^9	https://doi.org/10.1016/j.jhazmat.2022.130019	229

OTHER MATERIALS

Gallium-indium liquid metal	-	-	50	>70	100	30	3*10^9	https://doi.org/10.1002/smtd.202000147	236

Table S2 : Comparative overview of the structural properties, magnetic induction heating parameters, and thermal performance of various magnetic nanomaterials and bulk structures reported in the literature for biological applications, for non-biological applications as discussed in the chapter *MI for the synthesis of materials and industrial manufacturing*. The table uses color coding to distinguish the different application areas (e.g., dark red for Synthesis of Materials, red/orange for Thermal Curing, green for sustainable energy and grey for other materials). The

columns report the material composition, particle size, specific absorption rate (SAR), target and achieved temperatures for the specific process, operating frequency (f), magnetic field intensity (H), the H × f factor, and the corresponding references. Empty fields indicate that the relevant data could not be retrieved from the original publication.
